# Adverse outcomes after surgery after a cerebrovascular accident or acute coronary syndrome: a retrospective observational cohort study

**DOI:** 10.1016/j.bja.2024.08.029

**Published:** 2024-10-09

**Authors:** Matthew S. Luney, Christos V. Chalitsios, William Lindsay, Robert D. Sanders, Tricia M. McKeever, Iain K. Moppett

**Affiliations:** 1 Anaesthesia and Critical Care Section, Academic Unit of Injury, Inflammation and Repair, University of Nottingham, Nottingham, UK; 2 Nuffield Department of Orthopaedics, Rheumatology and Musculoskeletal Sciences, University of Oxford, Oxford, UK; 3 Academic Unit of Lifespan and Population Health, University of Nottingham, Nottingham, UK; 4 Nuffield Department of Clinical Neurosciences, University of Oxford, Oxford, UK; 5 Department of Anaesthesia, Nottingham University Hospitals NHS Trust, Nottingham, UK; 6 Speciality of Anaesthetics, Central Clinical School & NHMRC Clinical Trials Centre, University of Sydney, Camperdown, NSW, Australia; 7 Department of Anaesthetics, Royal Prince Alfred Hospital, Camperdown, NSW, Australia; 8 Institute of Academic Surgery, Royal Prince Alfred Hospital, Camperdown, NSW, Australia

**Keywords:** adverse outcomes, length of stay, myocardial infarction, postoperative recovery, readmission, stroke, surgery

## Abstract

**Background:**

Delaying surgery after a major cardiovascular event might reduce adverse postoperative outcomes. The time interval represents a potentially modifiable risk factor but is not well studied.

**Methods:**

This was a longitudinal retrospective population-based cohort study, linking data from Hospital Episode Statistics for NHS England and the Myocardial Ischaemia National Audit Project. Adults undergoing noncardiac, non-neurologic surgery in 2007–2018 were included. The time interval between a preoperative cardiovascular event and surgery was the main exposure. The outcomes of interest were acute coronary syndrome (ACS), acute myocardial infarction (AMI), cerebrovascular accident (CVA) within 1 year of surgery, unplanned readmission (at 30 days and 1 year), and prolonged length of stay. Multivariable logistic regression models with restricted cubic splines were used to estimate adjusted odds ratios (aORs; age, sex, socioeconomic deprivation, and comorbidities).

**Results:**

In total, 877 430 people had a previous cardiovascular event and 20 582 717 were without an event. CVA, ACS, and AMI in the year after elective surgery were more frequent after prior cardiovascular events (adjusted hazard ratio 2.12, 95% confidence interval [CI] 2.08–2.16). Prolonged hospital stay (aOR 1.36, 95% CI 1.35–1.38) and 30-day (aOR 1.28, 95% CI 1.25–1.30) and 1-yr (aOR 1.60, 95% CI 1.58–1.62) unplanned readmission were more common after major operations in those with a prior cardiovascular event. After adjusting for the time interval between preoperative events until surgery, elective operations within 37 months were associated with an increased risk of postoperative ACS or AMI. The risk of postoperative stroke plateaued after a 20-month interval until surgery, irrespective of surgical urgency.

**Conclusions:**

These observational data suggest increased adverse outcomes after a recent cardiovascular event can occur for up to 37 months after a major cardiovascular event.

**Editor's key points**

• The risk of adverse outcomes in noncardiac surgery for patients with previous major cardiovascular pathology is unclear.
• The authors interrogated two large UK outcome databases comprising >20 million people between 2007 and 2018.
• Stroke and acute coronary syndrome/myocardial infarction were more common *in the year after elective surgery* for 877 430 people with a previous cardiovascular event.
• These observational data suggest an increased risk of adverse outcomes for up to 37 months after surgery in people who have had a previous major cardiovascular event.

Prior cardiovascular events, including stroke and myocardial infarction, have long been recognised as factors increasing the risk of perioperative morbidity and mortality.^1^, ^2^, ^3^ However, the original data supporting this assertion regarding cerebrovascular accidents (CVAs; stroke) and acute coronary syndrome (ACS)^3^ have become less relevant in contemporary medical practice. This is attributed to the remarkable improvements in treating stroke and ACS, leading to better prognoses. Regarding myocardial infarction, the widespread availability of primary percutaneous coronary intervention has seen many more people living longer with ischaemic heart disease and reductions in crude 30-day mortality.^4^^,^^5^ Similarly, developments in the acute management of ischaemic stroke, such as thrombolysis and mechanical thrombectomy, have led to considerable advances in outcomes for these patients.^6^^,^^7^ The net result is that significantly comorbid patients are increasingly being put forward for surgery where this was previously not possible.^8^, ^9^, ^10^

In the UK, there is a high burden of ischaemic heart disease, and acute myocardial infarction remains a leading cause of morbidity and mortality.^11^ The cardiovascular stressors associated with surgical insult and anaesthesia are particularly significant for people with cardiovascular disease.^12^ Regarding cerebrovascular disease, more than 2 million people are estimated to be living with a stroke in the UK by 2035.^13^ It has also been recently demonstrated that covert stroke follows major surgery in 7% of individuals.^14^

Providing people with reliable, individualised estimates of their perioperative risks is a vital yet challenging component of preoperative preparation.^15^^,^^16^ Among patients clinically judged to be at high risk of perioperative complications such as stroke, when surveyed, very few recognised themselves to be high risk.^17^ In addition to determining whether to proceed with surgery, the timing of surgery in those with previous cardiovascular events involves balancing the increased perioperative risk closely after the event against the risk of disease progression or delay in the resolution of symptoms that indicate surgery. Previous research has focused only on elective surgery or a few individual procedures,^18^, ^19^, ^20^ particularly on postoperative mortality. As a result, there are limited international recommendations on the timing of noncardiac surgery for individuals with a previous cardiovascular event, particularly a lack of information regarding the risk of postoperative morbidity, specifically for those with a previous stroke or ACS.^21^ No studies have described the relationship between the time interval from preoperative cardiovascular event until surgery and length of stay or readmission rate; instead, they report only on patients who experienced ACSs or strokes after surgery.^12^

Here, our objective was to analyse individual-level data, building upon our prior work describing time-dependent postoperative mortality risks in those with prior cardiovascular events,^22^ to elucidate the dynamic relationship between preoperative cardiovascular events and cardiovascular outcomes, length of hospital stay, and unplanned readmissions. This study encompasses a broad and unselected population who underwent noncardiac, non-neurosurgical procedures with NHS funding in English hospitals between 2007 and 2018.

## Methods

### Study design and population

This is a longitudinal cohort study of all adults (≥18 yr) undergoing publicly funded surgery in England between April 1, 2007, and March 31, 2018, registered in Hospital Episode Statistics Admitted Patient Care (HES APC). The first surgical episode within the study window was identified from the OPCS-4 codes and acted as the index surgical event. People whose most recent cardiovascular event (ACS or stroke) was in the 10 years preceding their index surgery were identified using ICD-10 codes in HES APC. By linking Hospital Episode Statistics to the Myocardial Infarction National Audit Project (MINAP) database, further ACS cases were identified using MINAP-specific codes. More details about the study design can be found in the prospectively published protocol.^23^

### Data sources

HES APC is a national registry database containing details of all admissions to NHS hospitals in England and was formed in 1989^24^; data from 1997 to 2018 were extracted. MINAP is a national cardiac clinical audit that measures the process and outcomes of care of people diagnosed with ACS, including ST elevation and non-ST elevation myocardial infarctions; data from 2003 to 2018 were extracted.^25^ Mortality data were extracted from the Office for National Statistics between 2007 and 2019 (to assess mortality up to 1 year after surgery).

### Categories of surgery

To stratify the risks associated with differing invasiveness of operations, a classification based on OPCS-4 codes was used to define procedures as minor, moderate, or major, as already described by others.^26^ All OPCS-4 codes for hospital procedures were reviewed. Codes that were not surgical (e.g. radiotherapy, diagnostic imaging, or oxygen therapy) were excluded. The minor category comprised procedures that might be considered surgery, including interventional radiology procedures and endoscopies, but excluding noninvasive diagnostic procedures (e.g. cross-sectional imaging). The second category, ‘moderate’, included procedures routinely undertaken in an operating theatre and under general or regional anaesthesia. The third category, ‘major surgery’, included procedures that, because of duration or complexity, may often result in significant tissue injury, such as colectomy or arthroplasty. For subgroup analyses, we also classified common operations by surgical types, such as major lower limb joint replacement; vascular; gastrointestinal; gynaecological; urological; ear, nose, and throat (ENT); ophthalmological; and breast surgery. We excluded the following surgical categories *a priori*: cardiac, neurosurgical, carotid endarterectomy, obstetrics, tracheostomy, and percutaneous gastrostomy. Carotid endarterectomy operations were excluded because of the confounding by indication, as most of these procedures are performed in the days after a stroke and from which our data have insufficient granularity to observe risk in the order of days. We excluded percutaneous gastrostomy insertion and tracheostomy formation because of significant confounding from the bulbar dysfunction arising from some types of stroke. Finally, the surgical urgency was assessed according to the admission method recorded in HES APC.

### Primary exposure

The primary exposure was the time interval between the most recent preoperative cardiovascular event and index surgery.

### Outcomes

The adverse outcomes of interest in this study are ACS, myocardial infarction, or CVA within 1 yr of surgery, 30-day and 1-yr unplanned readmission rates, and prolonged length of stay. Prolonged length of stay is defined as the length of stay above the national upper quartile for the calendar year of index surgery, where>100 cases were performed per annum. Data were censored for operations admissions where the upper quartile was <2 days, as the data from the registry lacks the detail to detect differences in length of stay of only 1 day.

### Confounding

The potential confounders in all people from HES APC included as model covariates were age (continuous), sex, index of multiple deprivation (IMD; quintiles), ethnicity, and comorbidities (hypertension, atrial fibrillation, stable angina, peripheral vascular disease, valvular heart disease, congestive heart failure, respiratory failure, diabetes mellitus, renal failure, cancer, liver disease, and dementia). The Charlson Comorbidity Index was also calculated. For people with MINAP data, additional information on cardiovascular comorbidities was extracted.

### Statistical analysis

To compare those with no history of a cardiovascular event with those with an event in the preceding 10 yr, a Cox regression analysis was performed, and the results were reported as hazard ratio (HR) estimates and 95% confidence intervals (CIs). Incidence rates of adverse postoperative outcomes were calculated by dividing the number of outcomes by the follow-up (surgery to outcome/end of follow-up) person-yr. The Cox model assumption was tested using Schoenfeld residuals. For those with a prior cardiovascular event, multivariable logistic regression models were constructed for the association between the time interval from the most recent preoperative event until index surgery and adverse postoperative outcomes. The primary exposure was fitted as a continuous variable, with restricted cubic splines to account for the nonlinear relationship between the time elapsed and risk of adverse outcomes.^27^ Knots were placed at the 10th, 25th, 50th, 75th, and 90th percentiles, with the 50th (median) as the reference. People with an unrecorded age were excluded, and missing values for the IMD were retained by assigning them to a new category. Any missing data derived from the MINAP dataset were also assigned to a new category, as the percentage of missingness was high and imputation inappropriate. All statistical analyses were performed using R version 4.3.2 (R Foundation for Statistical Computing, Vienna, Austria).^28^ The statistical threshold for significance was set at *P*=0.05 for a two-tailed test.

## Results

### Participant characteristics

Βetween 2007 and 2018, a total of 21 460 147 people underwent surgery (83% elective), of which 877 430 (4.1%) had a history of cardiovascular events (Table 1 and Supplementary Fig. S1). People with a prior cardiovascular event were 19.3 yr older, were more often men, and had a higher prevalence of comorbidities (Table 1). Emergency surgery was more frequently performed for people with a prior cardiovascular event than those without a previous event (29% *vs* 16%, *P*<0.0001).

**Table 1:** Baseline characteristics of all people undergoing surgery between 2007 and 2017. Categorical data are expressed as *n* (%), continuous data are expressed as median [interquartile range].

	Overall (*N*=21 460 147)	No previous cardiovascular (*N*=20 582 717)	Previous cardiovascular (*N*=877 430)
Age (yr)	54 [38**–**69]	53 [37**–**68]	73 [64**–**81]
Sex			
Female	11 577 157 (54)	11 220 826 (55)	356 331 (41)
Male	9 882 990 (46)	9 361 891 (45)	521 099 (59)
Ethnicity			
White	16 367 832 (76)	15 617 378 (76)	750 454 (86)
Asian	3 217 494 (15)	3 150 564 (15)	66 930 (7.6)
Black	929 443 (4.3)	891 350 (4.3)	38 093 (4.3)
Other	475 541 (2.2)	464 016 (2.3)	11 525 (1.3)
Unknown	469 837 (2.2)	459 409 (2.2)	10 428 (1.2)
Index of multiple deprivation			
Least deprived	4 029 196 (19)	3 877 746 (19)	151 450 (17)
—	4 202 019 (20)	4 030 286 (20)	171 733 (20)
—	4 197 716 (20)	4 016 653 (20)	181 063 (21)
—	4 154 682 (19)	3 975 900 (19)	178 782 (20)
Most deprived	4 176 252 (19)	3 988 656 (19)	187 596 (21)
Unknown	700 282 (3)	693 476 (3)	6 806 (1)
Charlson Comorbidity Index			
0	16 401 423 (76)	15 936 777 (77)	464 646 (53)
1	3 936 433 (18)	3 673 547 (18)	262 886 (30)
2	889 944 (4)	782 878 (4)	107 066 (12)
≥3	232 347 (1)	189 515 (1)	42 832 (5)
Comorbidities			
Hypertension	3 804 608 (18)	3 389 416 (16)	415 192 (47)
Atrial fibrillation	594 199 (2.8)	480 216 (2.3)	113 983 (13)
Stable angina	50 786 (0.2)	4 016 (<0.1)	46 770 (5.3)
Dementia	242 844 (1.1)	193 834 (0.9)	49 010 (5.6)
Peripheral vascular disease	108 255 (0.5)	89 306 (0.4)	18 949 (2.2)
Valvular heart disease	212 441 (1.0)	151 632 (0.7)	60 809 (6.9)
Heart failure	1 920 406 (8.9)	1 796 263 (8.7)	124 143 (14)
Respiratory disease	1 455 402 (6.8)	1 295 413 (6.3)	159 989 (18)
Diabetes mellitus	420 301 (2.0)	353 673 (1.7)	66 628 (7.6)
Chronic kidney disease	1 153 917 (5.4)	1 091 083 (5.3)	62 834 (7.2)
Active malignancy	199 401 (0.9)	189 443 (0.9)	9 958 (1.1)
Chronic liver disease	208 294 (1.0)	174 318 (0.8)	33 976 (3.9)
Surgical invasiveness			
Minor	7 493 661 (35)	7 142 867 (35)	350 794 (40)
Moderate	9 413 447 (44)	9 056 811 (44)	356 636 (41)
Major	4 553 039 (21)	4 383 039 (21)	170 000 (19)
Surgical urgency			
Elective	17 833 826 (83)	17 208 304 (84)	625 522 (71)
Emergency	3 626 321 (17)	3 374 413 (16)	251 908 (29)

### Primary outcome: time elapsed from cardiovascular event to surgery and adverse outcomes

**Fig 1:**
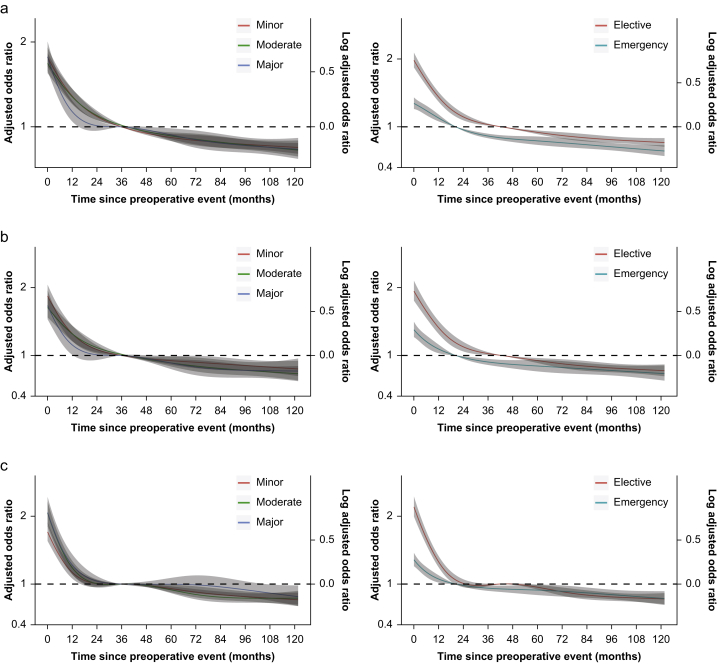
Restricted cubic splines logistic regression for 1-yr risk of (a) acute coronary syndrome, (b) acute myocardial infarction, and (c) cerebrovascular accident after surgery by the time between the most recent cardiovascular event stratified by surgery invasiveness and urgency. The spline was adjusted for age, sex, index of multiple deprivation, hypertension, atrial fibrillation, stable angina, peripheral vascular disease, valvular heart disease, congestive heart failure, respiratory diseases, diabetes mellitus, renal failure, cancer, liver disease, and dementia. The median time between the event and surgery served as the reference.

In people with a previous cardiovascular event, the adjusted odds associated with adverse outcomes decreased as the time interval between the preoperative event and operation increased (Fig. 1). The odds of ACS and myocardial infarction within 1 yr after surgery levelled off for patients whose preoperative event was more than 37 months before minor and elective surgery. Considering elective and emergency operations, the time interval associated with increased risk of ACS and myocardial infarction reached a new baseline at 42 months for elective surgery and 20 months for emergency surgery, respectively. However, the risk of stroke within 1 yr was diminished after 20 months, irrespective of the urgency or severity of the surgery. The time did not change when stratified by sex (Supplementary Figs. S2 and S3), but the time to plateau differed between surgical specialities (Supplementary Figs. S4–S6).

### Adverse outcomes among those with and without a previous cardiovascular event

Adverse cardiovascular outcome rates were higher in people with a cardiovascular event before surgery than those without (Table 2). These findings persisted following adjustment for confounders with CVA within 1 yr after a moderate surgery to have the highest risk when stratified by invasiveness (adjusted HR [aHR] 2.07, 95% CI 2.01–2.13). Considering the surgical urgency, the relative risk was greater in those undergoing elective surgery (ACS: aHR 2.12, 95% CI 2.08–2.16; AMI: aHR 2.12, 95% CI 2.07–2.16; CVA: aHR 2.12, 95% CI 2.07–2.17) than emergency surgery (ACS: aHR 1.76, 95% CI 1.71–1.81; AMI: aHR 1.75, 95% CI 1.71–1.80; CVA: aHR 1.71, 95% CI 1.66–1.76).

**Table 2:** Hazard ratios of adverse postoperative cardiovascular outcomes within 1 yr after surgery comparing people with and without a cardiovascular event before the surgery. ACS, acute coronary syndrome (unstable angina and myocardial infarction); AMI, acute myocardial infarction; CI, confidence interval; CVA, *c*erebrovascular accident (ischaemic, haemorrhagic, transient ischaemic attack, or stroke not otherwise specified); HR, hazard ratio. ∗Adjusted for age, sex, index of multiple deprivation, hypertension, atrial fibrillation, stable angina, peripheral vascular disease, valvular heart disease, congestive heart failure, respiratory diseases, diabetes mellitus, renal failure, cancer, liver disease, and dementia. ^†^All *P*-values <0.0001.

Adverse outcomes within 1 yr of surgery	Previous cardiovascular		No previous cardiovascular		Unadjusted HR (95% CI)	Adjusted HR∗^,†^ (95% CI)
	Events	Incidence per 1000 person-yr	Events	Incidence per 1000 person-yr		
Minor surgery						
ACS	10 514	30.9 (30.3–31.5)	39 586	5.6 (5.5–5.7)	5.47 (5.36–5.59)	2.06 (2.02–2.11)
AMI	5 542	15.9 (15.5–16.4)	27 602	3.9 (3.8–4)	4.11 (3.99–4.23)	2.06 (2.01–2.11)
CVA	6 405	18.6 (18.1–19.1)	26 227	3.7 (3.6–3.8)	5.01 (4.87–5.15)	1.94 (1.88–1.99)
Moderate surgery						
ACS	9 182	26.4 (25.9–27)	39 316	4.4 (4.3–4.5)	5.99 (5.86–6.13)	1.95(1.39–2.00)
AMI	5 219	14.7 (14.3–15.1)	28 781	3.2 (3.1–3.3)	4.63 (4.50–4.77)	1.94 (1.89–1.99)
CVA	6 443	18.4 (17.9–18.9)	29 027	3.2 (3.1–3.3)	5.68 (5.53–5.83)	2.07 (2.01–2.13)
Major surgery						
ACS	4 035	24.3 (23.6–25.1)	18 634	4.3 (4.2–4.4)	5.64 (5.45–5.83)	1.91 (1.54–1.98)
AMI	2 354	14 (13.4–14.6)	13 735	3.1 (3–3.2)	4.44 (4.25–4.64)	1.90 (1.83–1.97)
CVA	3 231	19.4 (18.7–20.1)	15 515	3.6 (3.5–3.7)	5.41 (5.21–5.62)	1.95 (1.88–2.04)
Elective surgery						
ACS	15 275	25 (24.6–25.4)	73 704	4.3 (4.2–4.4)	5.76 (5.66–5.86)	2.12 (2.08–2.16)
AMI	8 049	12.9 (12.7–13.2)	52 336	3.1 (3–3.2)	4.25 (4.15–4.35)	2.12 (2.07–2.16)
CVA	9 413	15.3 (15–15.6)	47 826	2.8 (2.7–2.9)	5.45 (5.33–5.57)	2.12 (2.07–2.17)
Emergency surgery						
ACS	8 456	34.7 (34–35.5)	23 832	7.1 (7–7.2)	4.82 (4.70–4.94)	1.76 (1.71–1.81)
AMI	5 066	20.4 (19.8–20.9)	17 782	5.3 (5.2–5.4)	3.84 (3.73–3.97)	1.75 (1.71–1.80)
CVA	6 666	27.2 (26.5–27.8)	22 943	6.8 (6.7–6.9)	3.93 (3.82–4.04)	1.71 (1.66–1.76)

### Length of stay and readmission

**Fig 2:**
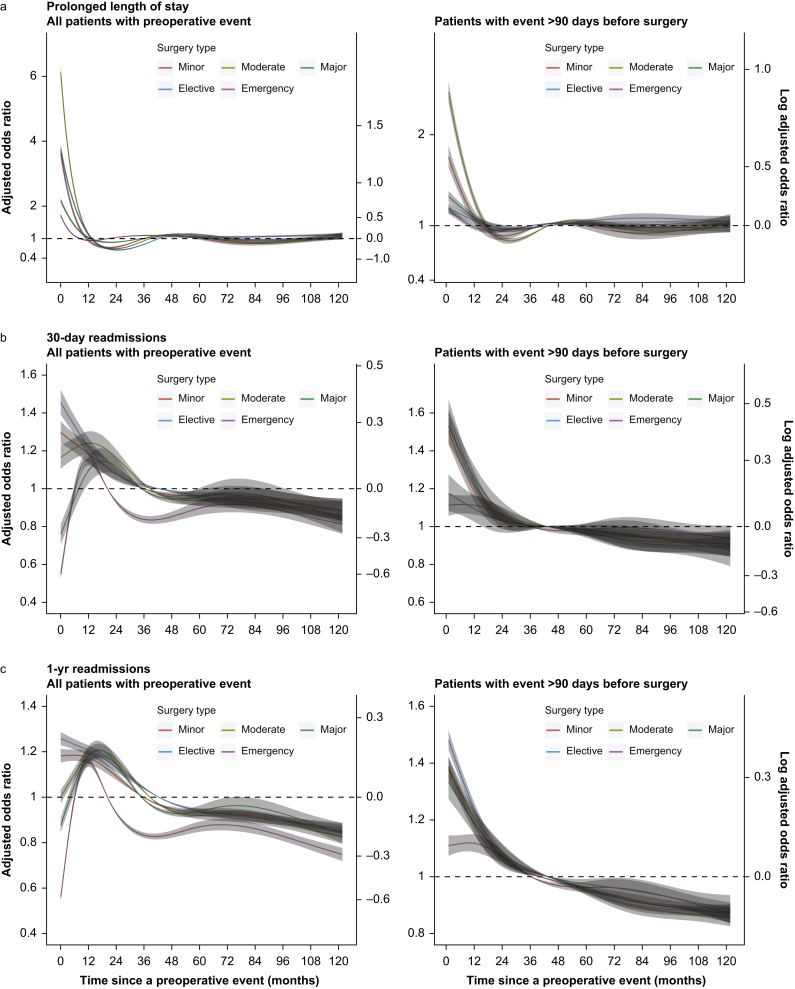
Restricted cubic splines logistic regression for (a) risk of prolonged length of stay after surgery, (b) 30-day readmission, and (c) 1-yr readmission by the time between the most recent cardiovascular event stratified by surgery invasiveness and urgency. The spline was adjusted for age, sex, index of multiple deprivation, hypertension, atrial fibrillation, stable angina, peripheral vascular disease, valvular heart disease, congestive heart failure, respiratory diseases, diabetes mellitus, renal failure, cancer, liver disease, and dementia. The median time between the event and surgery served as the reference.

People with a cardiovascular event before surgery, compared with those without an event, had higher odds of prolonged length of stay and readmission (Table 3). Considering the surgical invasiveness, prolonged length of stay and readmission had higher odds in moderate (adjusted odds ratio [aOR] 1.69, 95% CI 1.67–1.72) and minor surgery (within 1 yr: aOR 1.84, 95% CI 1.82–1.86; within 30 days: aOR 1.57, 95% CI 1.54–1.59), respectively. The odds of prolonged length of stay were similar in elective and emergency surgery; however, elective surgery had higher odds of unplanned readmission (within 1 yr: aOR 1.94, 95% CI 1.93–1.96; within 30 days: aOR 1.59, 95% CI 1.57–1.61). The rate of unplanned readmissions decreased as the time interval between patients' previous cardiovascular event before surgery increased (Fig. 2), across all levels of operative severity and urgency. However, patients undergoing major, emergency, or both operations within 90 days of their cardiovascular event had a lower readmission rate than the rest of the cardiovascular disease cohort (Fig. 2). This paradoxical effect was not replicated when only patients with greater than 90 days between their event and surgery were modelled.

**Table 3:** Prolonged length of stay and unplanned readmission rates comparing people with and without a cardiovascular event before the surgery. ∗Adjusted for age, sex, index of multiple deprivation, hypertension, atrial fibrillation, stable angina, peripheral vascular disease, valvular heart disease, congestive heart failure, respiratory diseases, diabetes mellitus, renal failure, cancer, liver disease, and dementia. ^†^All *P*-values <0.001.

Adverse outcomes	No previous cardiovascular	Previous cardiovascular	Unadjusted OR (95% CI)	Adjusted OR∗^,†^ (95% CI)
Minor surgery				
Prolonged length of stay	74 209 (1.0%)	14 010 (4.0%)	3.96 (3.89–4.03)	1.64 (1.61–1.68)
Readmission within 30 days	190 485 (2.7%)	21 810 (6.2%)	2.42 (2.38–2.45)	1.57 (1.54–1.59)
Readmission within 1 yr	679 684 (16%)	55 958 (33%)	3.09 (3.07–3.11)	1.84 (1.82–1.86)
Moderate surgery				
Prolonged length of stay	243 169 (2.7%)	36 776 (10%)	4.17 (4.12–4.21)	1.69 (1.67–1.72)
Readmission within 30 days	213 071 (2.4%)	16 714 (4.7%)	2.04 (2.01–2.07)	1.47 (1.45–1.50)
Readmission within 1 yr	154 498 (3.5%)	9 325 (5.5%)	2.99 (2.97–3.02)	1.77 (1.75–1.78)
Major surgery				
Prolonged length of stay	679 684 (16%)	55 958 (33%)	2.11 (2.08–2.13)	1.36 (1.35–1.38)
Readmission within 30 days	769 219 (8.5%)	77 550 (22%)	1.59 (1.55–1.62)	1.28 (1.25–1.30)
Readmission within 1 yr	485 511 (11%)	41 233 (24%)	2.57 (2.54–2.60)	1.60 (1.58–1.62)
Elective surgery				
Prolonged length of stay	404 306 (2.4%)	30 185 (4.8%)	2.11 (2.08–2.13)	1.09 (1.07–1.10)
Readmission within 30 days	333 717 (1.9%)	25 661 (4.1%)	2.16 (2.14–2.19)	1.59 (1.57–1.61)
Readmission within 1 yr	1 419 211 (8.2%)	133 548 (21%)	3.02 (3.00–3.04)	1.94 (1.93–1.96)
Emergency surgery				
Prolonged length of stay	592 756 (18%)	76 559 (31%)	2.04 (2.02–2.06)	1.12 (1.11–1.13)
Readmission within 30 days	224 337 (6.6%)	22 188 (8.8%)	1.36 (1.34–1.38)	1.13 (1.11–1.15)
Readmission within 1 yr	685 891 (20%)	88 573 (35%)	2.13 (2.11–2.14)	1.33 (1.32–1.35)

## Discussion

Using routinely collected electronic health records from more than 21 million people undergoing surgery in England between 2007 and 2018, we demonstrated that the risk of postoperative ACS, myocardial infarction, or stroke is two-fold higher in patients with a previous cardiovascular event. The associated risk is highest in the first 3 years after a preoperative cardiovascular event, reaching a plateau thereafter. After adjustments, prolonged lengths of stay and readmissions within 30 days and 1 year after surgery were 1.3-, 1.5-, and 1.8-fold more common in those with a previous stroke or ACS.

Our study reports that in patients presenting for surgery within 3 years of a stroke or ACS, they should be counselled about not only the increased mortality risk,^22^ but also risks of recurrent stroke, ACS, prolonged admission, and readmissions in the year after the proposed surgery. The temporal nature of this risk must be acknowledged, particularly that postponement, where surgery permits, by up to 12 months after an event is associated with large reductions in adverse outcomes. These findings agree with prior large-scale data from North American and Danish healthcare registries.^19^^,^^20^ Unlike this previous registry work, which focused only on elective surgical settings, our analysis of both elective and emergency surgery allowed us to show that the postoperative risks associated with recent cardiovascular events are present for longer in those undergoing elective rather than emergency surgery where the acute surgical pathology predominates the risk profile. Notably, the aORs of adverse events were globally lower in our study than in these previous registry studies. This difference arises from the choice of the reference value. The risks of adverse outcomes in the studies by Glance and colleagues^19^ and Jørgensen and colleagues^20^ were compared with those of participants who had never had a preoperative cardiovascular event, despite this being a risk state that patients can never return to. In contrast, we referenced risk to patients at the median (50th centile) time point since the preoperative event, thereby providing a more interpretable finding for surgical candidates who have a history of ACS or stroke.

Our data challenge the current American Heart Association and European Society of Cardiology (ESC) guidance that elective surgery delayed for only 6–9 months confers an adequate reduction in perioperative risk with respect to recurrent stroke or ACS.^21^^,^^29^ Absent any randomised controlled trials of deferred *vs* routine timing of noncardiac surgery, the authors of both the North American and European guidelines were restricted to the aforementioned observational studies of Jørgensen and colleagues^20^ and Glance and colleagues,^19^ although in the later guideline, authors referred to previous ESC guidelines for justification of their recommendations rather than to primary literature.^30^^,^^31^

The risk of prolonged length of stay in patients with previous stroke or ACS has long been recognised.^32^ However, data regarding the duration of this increased risk are limited. We found that length of stay was prolonged in those who underwent surgery within 1 year of a cardiovascular event, and the risk of unplanned readmissions remained increased for 3 years after a cardiovascular event, which may have implications for resource allocation and counselling potential candidates for surgery.

To the best of our knowledge, this study represents the most extensive investigation into the time-dependent aspect of adverse postoperative outcomes in people with prior cardiovascular events. A particular strength of our study lies in the size of our population, encompassing the whole adult population of England who underwent noncardiac, non-neurological, and nonobstetric surgery between 2007 and 2018. Our study's demographic composition is similar to that of substantial surgical populations in North America^33^ and Western Europe.^20^ Our permissive inclusion criteria ensure the generalisability of our findings to a wide range of populations. Because of the substantial size of our study cohort and the rate of adverse outcomes, we were able to account for many confounding variables and offered dependable estimates for subgroup analyses. This enables clinicians to tailor their approach based on specific surgical specialities. We acknowledge several limitations in our study. It is fundamentally observational and retrospective, warranting caution when attempting to establish causal relationships between surgery timing and postoperative outcomes. Some of the variation in adverse outcomes over time may not be linked to surgery; for instance, risks decrease naturally after a stroke or myocardial infarction. Consequently, unavoidable survivor bias will be present, as those still alive and fit enough to undergo surgery after their cardiovascular event may represent a more robust cohort than those undergoing surgery at a different time point. Nonetheless, it is important to emphasise that our data are presented to provide a reference for the temporal dynamics of preoperative cardiovascular risk factors to facilitate shared decision-making. We do not presume to mandate when or whether an individual is offered surgery. Potential inaccuracies in registry coding could lead to under-representation of comorbidity diagnoses. However, cross-referencing data between HES and MINAP has allowed us to validate our findings across different registries, reaffirming the association between increasing time since the cardiovascular event and declining postoperative adverse outcomes. We are limited by the lack of certain data not held in HES such as patients' medication history and perioperative anticoagulation management. Some people will also choose to undergo surgery shortly after a cardiovascular event despite the high-risk nature and will be represented in the data. Nevertheless, we have stratified the data by surgical severity and urgency to mitigate this effect as far as possible. Conversely, some people may have had surgery without their cardiovascular event history being known. Although not expected to be highly prevalent, it reflects the occasional reality of incomplete medical histories in daily practice.

In conclusion, patients appear to be at increased risk of postoperative complications for 3 years after a cardiovascular event, despite adjustment for age, sex, comorbidities, and social deprivation. This risk is present for longer in those undergoing elective surgery after their event, which represents the challenge between the goals of treating surgical pathology before it worsens and avoiding postoperative adverse events in a significantly co-morbid population.

## Authors’ contributions

Had full access to all the study data and take full responsibility for the integrity of the data and the accuracy of the data analysis: MSL, CVC

Contributed equally to this paper as joint first authors: MSL, CVC

Conception and design: TMM, IM

Acquisition of data: MSL, WL, IM

Analysis of data: MSL, CVC

Interpretation of data: MSL, CVC, ΤΜΜ, IM

Drafting the manuscript: MSL, CVC

Revision for important intellectual content and approval of the version to be published: all authors

## Declaration of interest

RDS is an editor of the *British Journal of Anaesthesia*. The other authors declare that they have no conflicts of interest.

## Funding

BJA/RCoA Project Grant (WKR0-2018-0023); NIAA Health Services Research Centre fellowships funded by Nottingham University Hospitals (to MSL and WL).

## Data availability

The authors are not permitted to share the data publicly. Research ethics committee approval is required to access the data, including an application to the HRA Confidentiality Advisory Group with respect to Section 251 (4) of the NHS Act 2006.
