# Supplementary material for: Adverse outcomes after surgery after a cerebrovascular accident or acute coronary syndrome: a retrospective observational cohort study
Source: Br J Anaesth. 2024 Oct 9;134(1):63–71. doi: 10.1016/j.bja.2024.08.029 (PMC11718364; doi:10.1016/j.bja.2024.08.029)
Supplement: Multimedia component 1 [file mmc1.docx]

**Adverse outcomes after surgery in people with a previous cerebrovascular accident or acute coronary syndrome**

Matthew S. Luney ^#^, Christos V. Chalitsios ^#^, William Lindsay, Robert D. Sanders, Tricia M. McKeever, Iain Moppett

*^#^ M.S.L and C.V.C contributed equally to this paper, joint first authors.*

Table of Contents

[Flow chart 3](#_Toc164418636)

[Adverse outcomes by sex 4](#_Toc164418637)

[Men 4](#_Toc164418638)

[Women 5](#_Toc164418639)

[Adverse outcomes by surgical speciality 6](#_Toc164418640)

[Number of pre-specified operations by specialty 6](#_Toc164418641)

[Urological surgery 7](#_Toc164418642)

[Gynaecological surgery 10](#_Toc164418643)

[Ophthalmological surgery 13](#_Toc164418644)

[Otolaryngology surgery 16](#_Toc164418645)

[Orthopaedic surgery 19](#_Toc164418646)

[Adverse outcomes by subtype of orthopaedic surgery 22](#_Toc164418647)

[Primary hip 22](#_Toc164418648)

[Revision conversion hip 25](#_Toc164418649)

[Vascular surgery 27](#_Toc164418650)

[Adverse outcomes by subtype of vascular surgery 30](#_Toc164418651)

[Abdominal Aortic Aneurysm (AAA) open 30](#_Toc164418652)

[Lower limb bypass 33](#_Toc164418653)

[Gastrointestinal surgery 36](#_Toc164418654)

[Adverse outcomes by subtype of gastrointestinal surgery 39](#_Toc164418655)

[Colorectal major resection 39](#_Toc164418656)

[Cholecystectomy 42](#_Toc164418657)

[Adverse outcomes by subtype of cardiovascular event 45](#_Toc164418658)

[Haemorrhagic stroke 46](#_Toc164418659)

[Ischaemic stroke 49](#_Toc164418660)

[Unspecified stroke 52](#_Toc164418661)

[Myocardial infarction 55](#_Toc164418662)

[Transient ischaemic attack 58](#_Toc164418663)

[Unstable angina 60](#_Toc164418664)

[Readmission – sensitivity analysis 63](#_Toc164418665)

## Flow chart


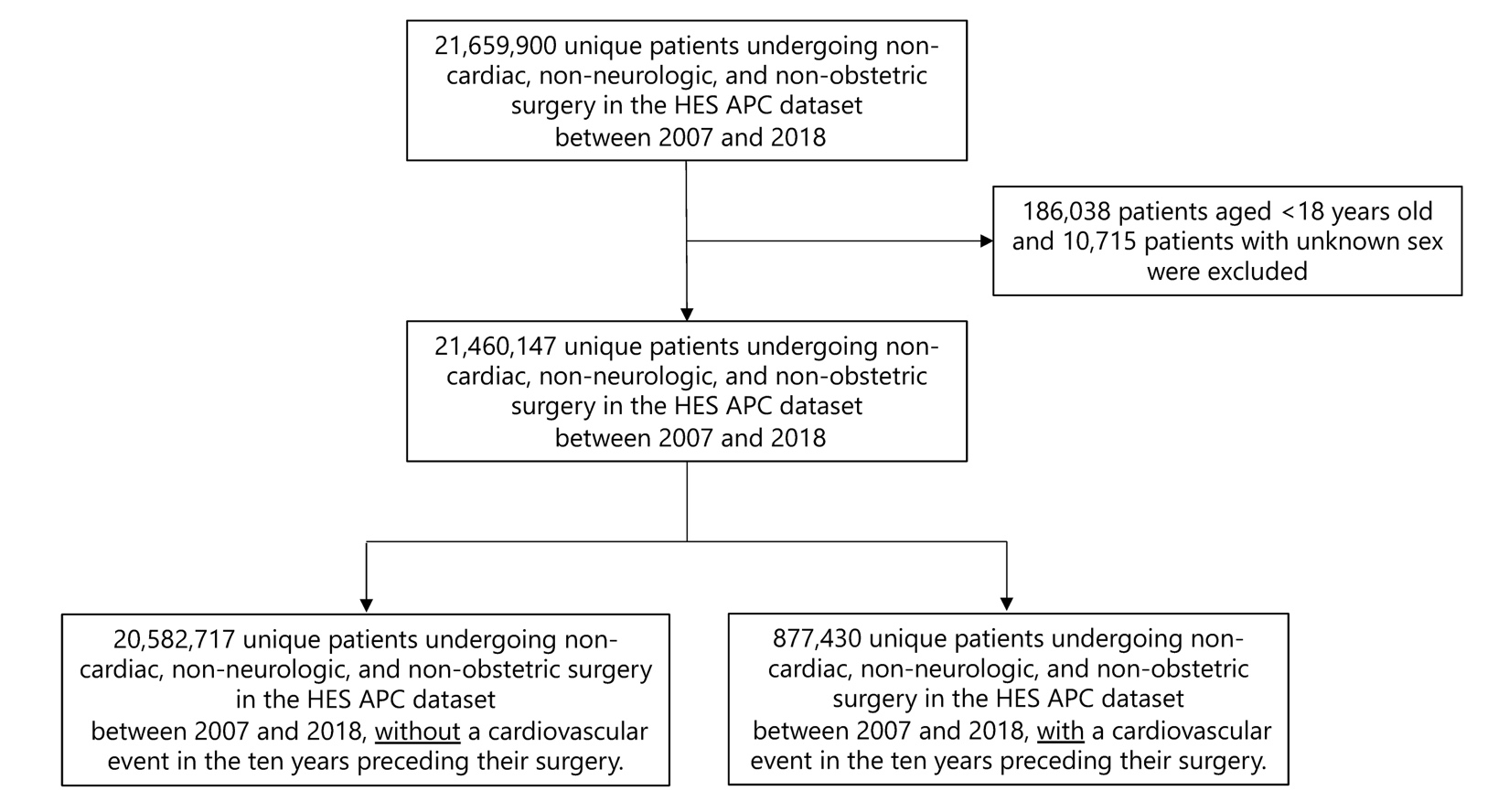


**Figure 1**. Study flowchart

## Adverse outcomes by sex

### Men


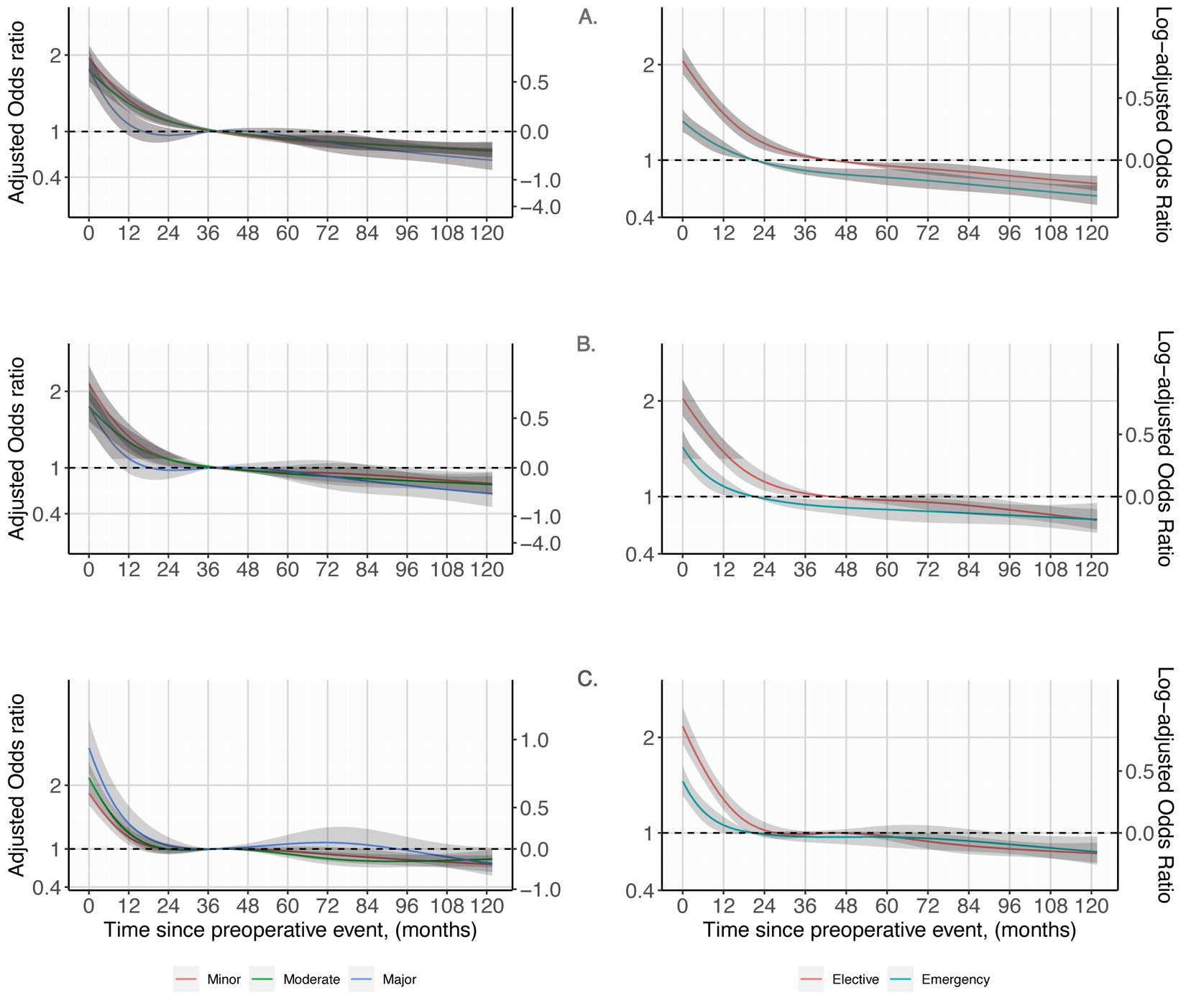


**Figure 2**. Restricted cubic splines logistic regression for 1-year risk of (A) Acute Coronary Syndrome, (B) Acute Myocardial infarction, and (C) Cerebrovascular accident after surgery by the time between the most recent cardiovascular event stratified by surgery invasiveness and urgency among men. The spline was adjusted for age, sex, index of multiple deprivation, hypertension, atrial fibrillation, stable angina, peripheral vascular disease, valvular heart disease, congestive heart failure, respiratory diseases, diabetes mellitus, renal failure, cancer, liver disease, and dementia. The median time between the event and surgery served as the reference.

### Women


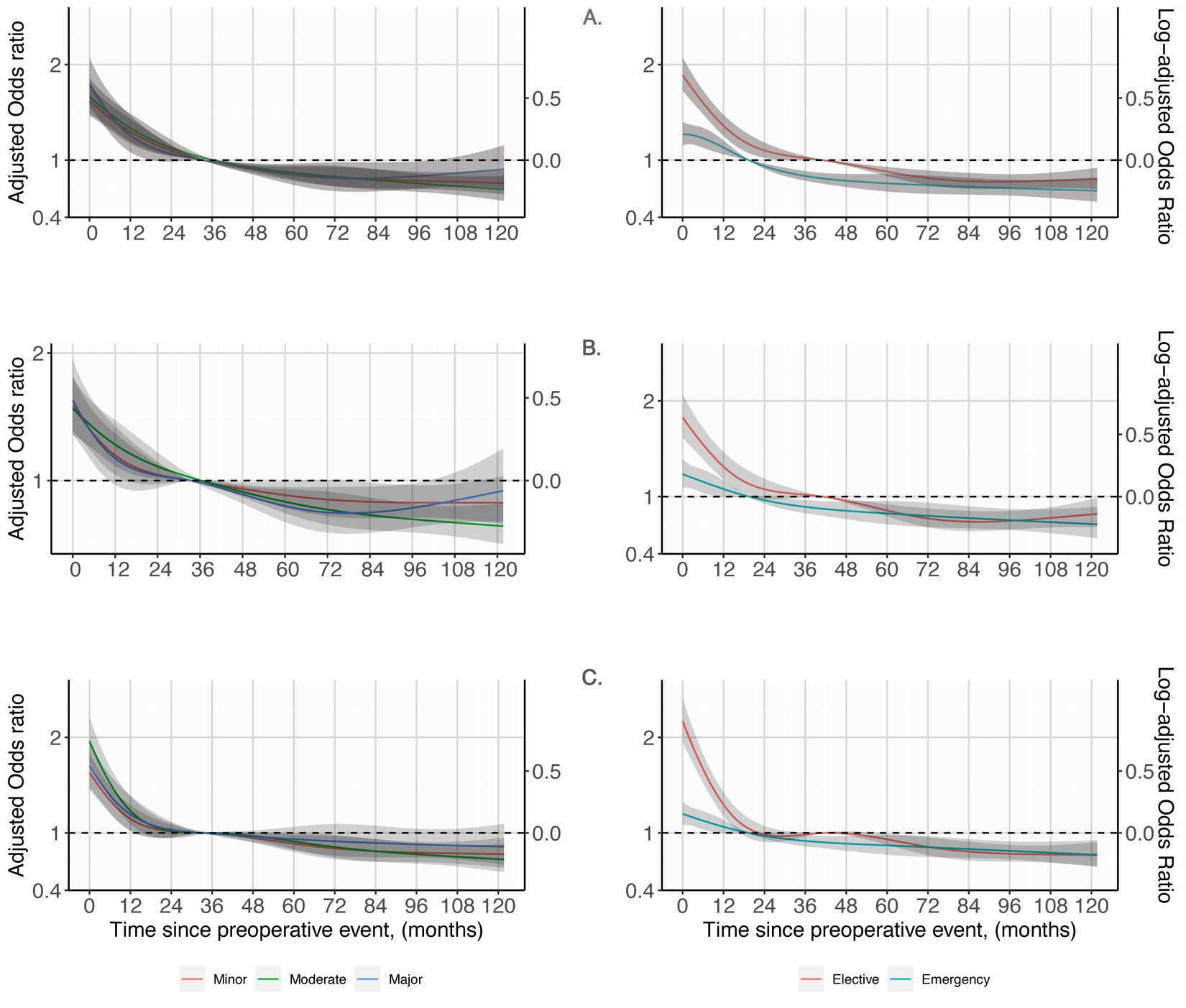


**Figure 3**. Restricted cubic splines logistic regression for 1-year risk of (A) Acute Coronary Syndrome, (B) Acute Myocardial infarction, and (C) Cerebrovascular accident after surgery by the time between the most recent cardiovascular event stratified by surgery invasiveness and urgency among women. The spline was adjusted for age, sex, index of multiple deprivation, hypertension, atrial fibrillation, stable angina, peripheral vascular disease, valvular heart disease, congestive heart failure, respiratory diseases, diabetes mellitus, renal failure, cancer, liver disease, and dementia. The median time between the event and surgery served as the reference.

## Adverse outcomes by surgical speciality

### Number of pre-specified operations by specialty

**Table 1.** Absolute numbers of operations by surgical speciality among those with a previous cardiovascular event.

|  | **Surgery invasiveness** | | | **Surgery urgency** | |
| --- | --- | --- | --- | --- | --- |
| **Surgery specialty** | **Minor** | **Intermediate** | **Major** | **Elective** | **Emergency** |
| **Urological** | 45,670 (49.3) | 39,436 (42.6) | 7,524 (8.1) | 75,590 (78.6) | 17,040 (21.4) |
| **Gynaecological** | 4,281 (30) | 5,736 (40.3) | 4,206 (29.7) | 12,555 (88.3) | 1,668 (11.7) |
| **Ophthalmological** | NA | 118,201 (100) | NA | 116,555 (98.6) | 1,646 (1.4) |
| **Otolaryngology** | 8,257 (28.3) | 19,140 (65.6) | 1,794 (6.1) | 18,468 (63.3) | 10,723 (36.7) |
| **Orthopaedic** | NA | NA | 24,419 (100) | 22,750 (92.4) | 1849 (7.6) |
| **Gastrointestinal** | NA | NA | 9,699 (100) | 5,711 (58.9) | 3,988 (41.1) |
| **Vascular** | NA | NA | 8,483 (100) | 4,631 (54.6) | 3,852 (45.4) |

*Abbreviations: AAA, Abdominal Aortic Aneurysm; EVAR, Endovascular Aneurysm Repair.*

*Data are expressed as No. (%).*

### Urological surgery


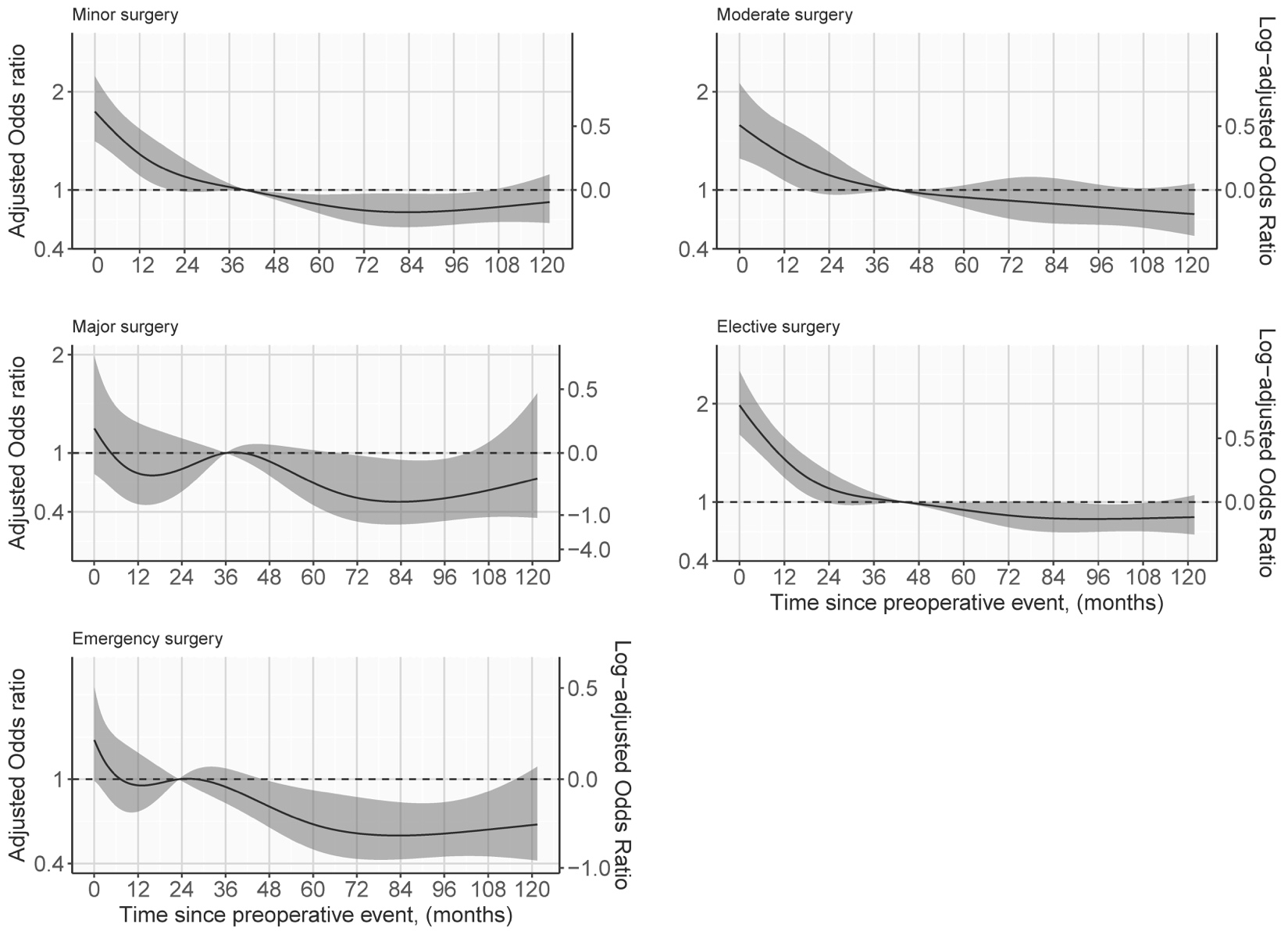


**Figure 4**. Restricted cubic splines logistic regression for 1-year risk of Acute Coronary Syndrome after surgery by the time between the most recent cardiovascular event stratified by surgery invasiveness and urgency. The spline was adjusted for age, sex, index of multiple deprivation, hypertension, atrial fibrillation, stable angina, peripheral vascular disease, valvular heart disease, congestive heart failure, respiratory diseases, diabetes mellitus, renal failure, cancer, liver disease, and dementia. The median time between the event and surgery served as the reference.


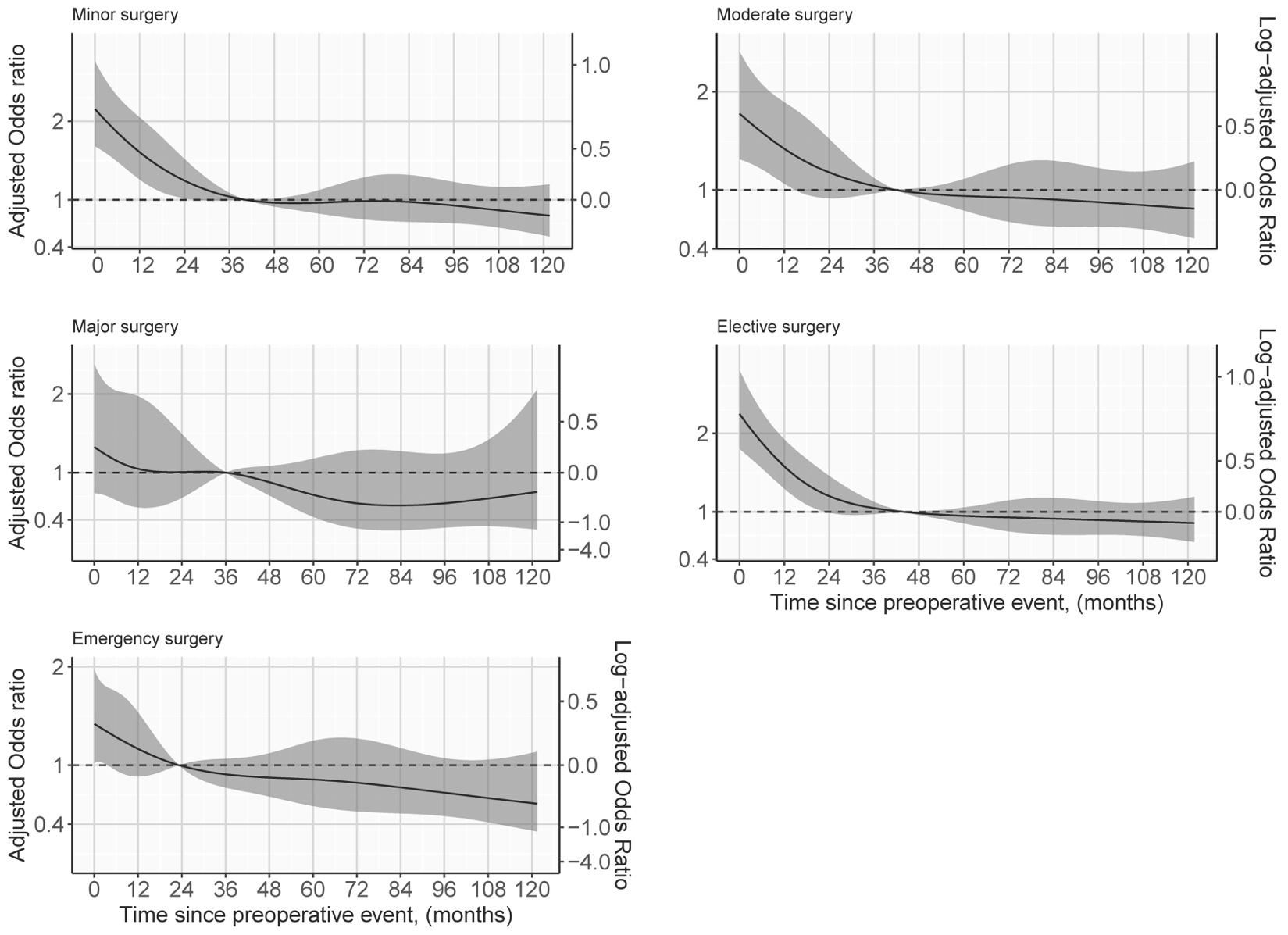


**Figure 5**. Restricted cubic splines logistic regression for 1-year risk of Acute Myocardial infarction after surgery by the time between the most recent cardiovascular event stratified by surgery invasiveness and urgency. The spline was adjusted for age, sex, index of multiple deprivation, hypertension, atrial fibrillation, stable angina, peripheral vascular disease, valvular heart disease, congestive heart failure, respiratory diseases, diabetes mellitus, renal failure, cancer, liver disease, and dementia. The median time between the event and surgery served as the reference.


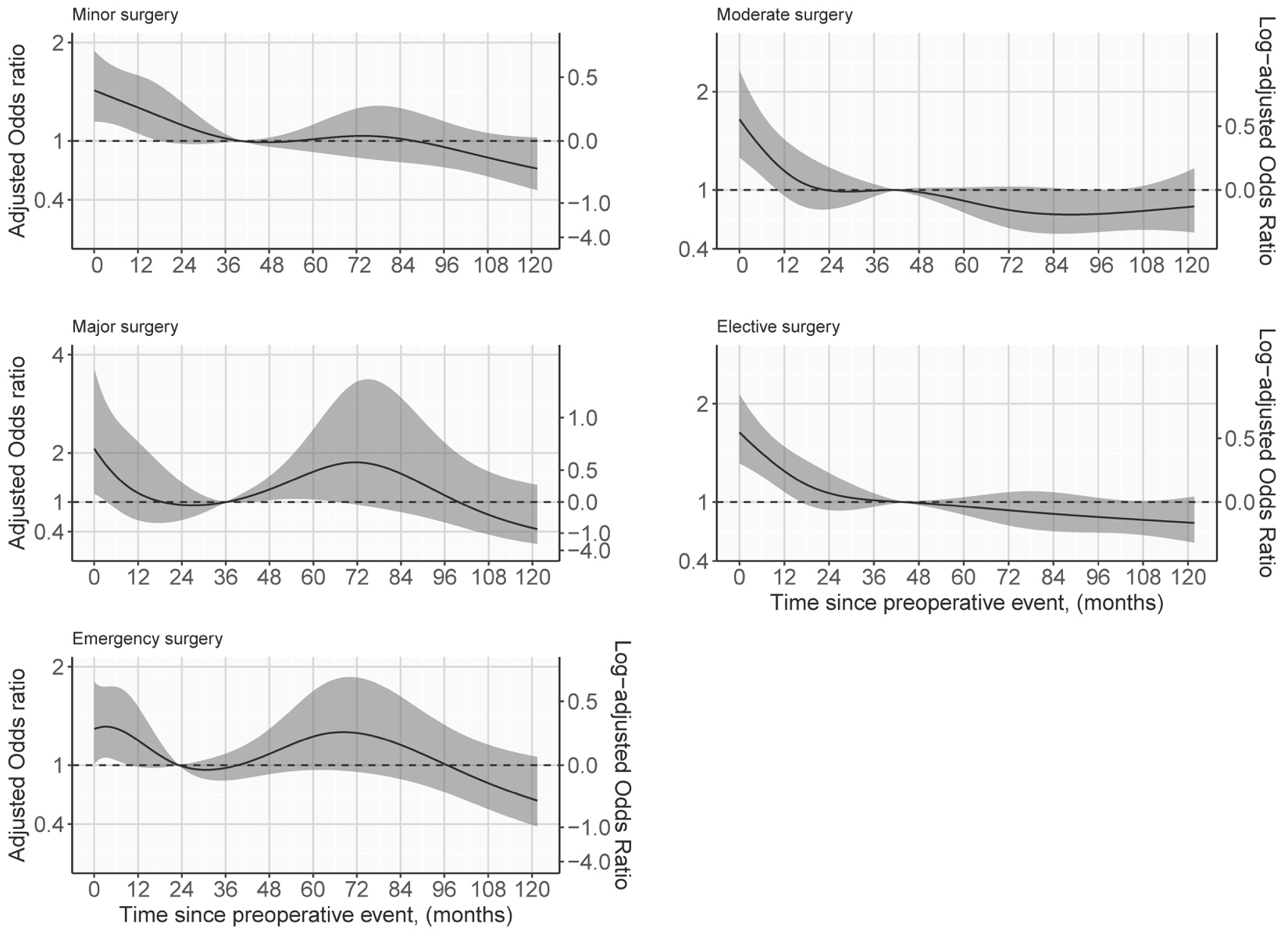


**Figure 6**. Restricted cubic splines logistic regression for 1-year risk of cerebrovascular accident after surgery by the time between the most recent cardiovascular event stratified by surgery invasiveness and urgency. The spline was adjusted for age, sex, index of multiple deprivation, hypertension, atrial fibrillation, stable angina, peripheral vascular disease, valvular heart disease, congestive heart failure, respiratory diseases, diabetes mellitus, renal failure, cancer, liver disease, and dementia. The median time between the event and surgery served as the reference.

### Gynaecological surgery


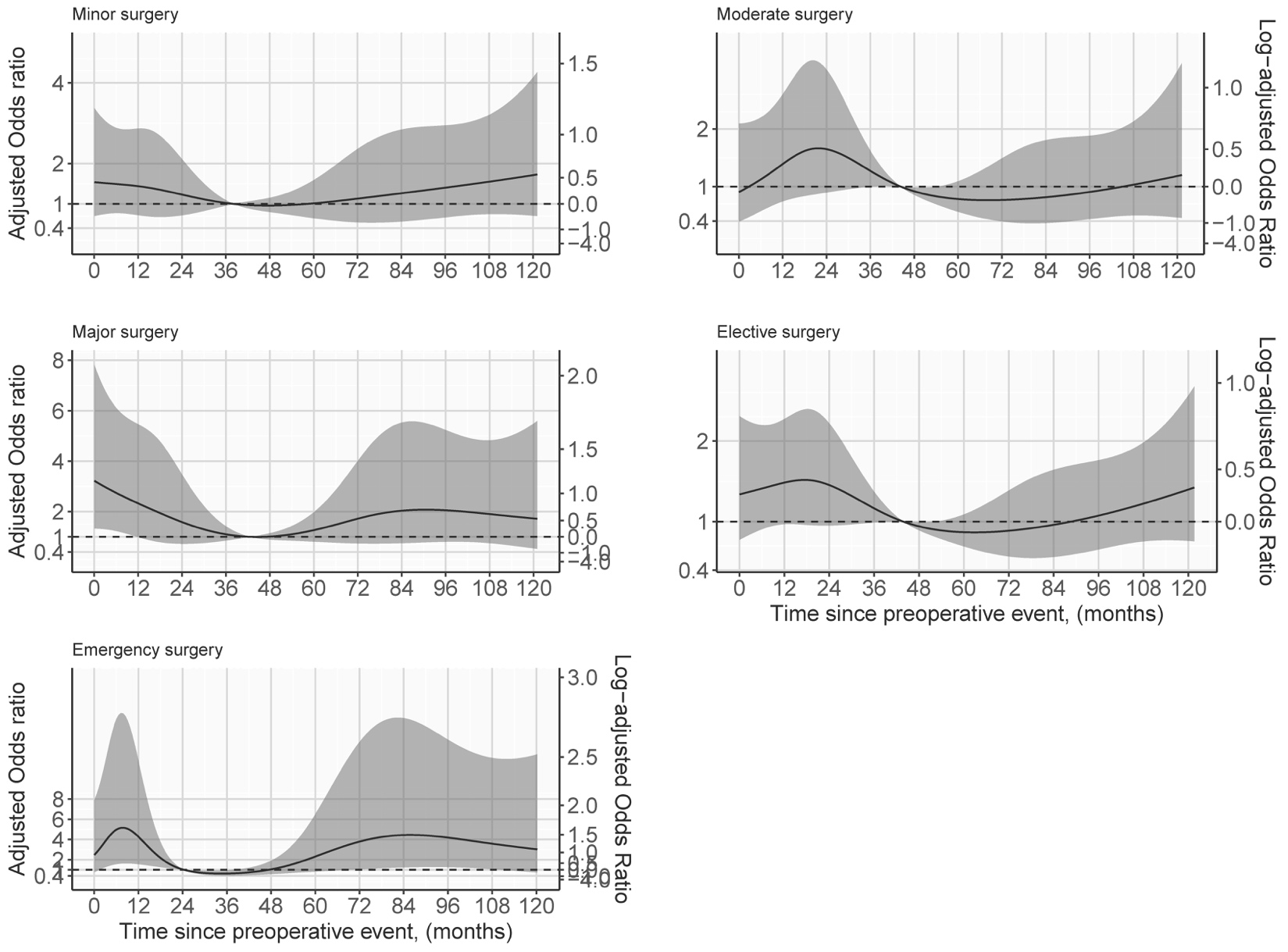


**Figure 7.** Restricted cubic splines logistic regression for 1-year risk of Acute Coronary Syndrome after surgery by the time between the most recent cardiovascular event stratified by surgery invasiveness and urgency. The spline was adjusted for age, sex, index of multiple deprivation, hypertension, atrial fibrillation, stable angina, peripheral vascular disease, valvular heart disease, congestive heart failure, respiratory diseases, diabetes mellitus, renal failure, cancer, liver disease, and dementia. The median time between the event and surgery served as the reference.


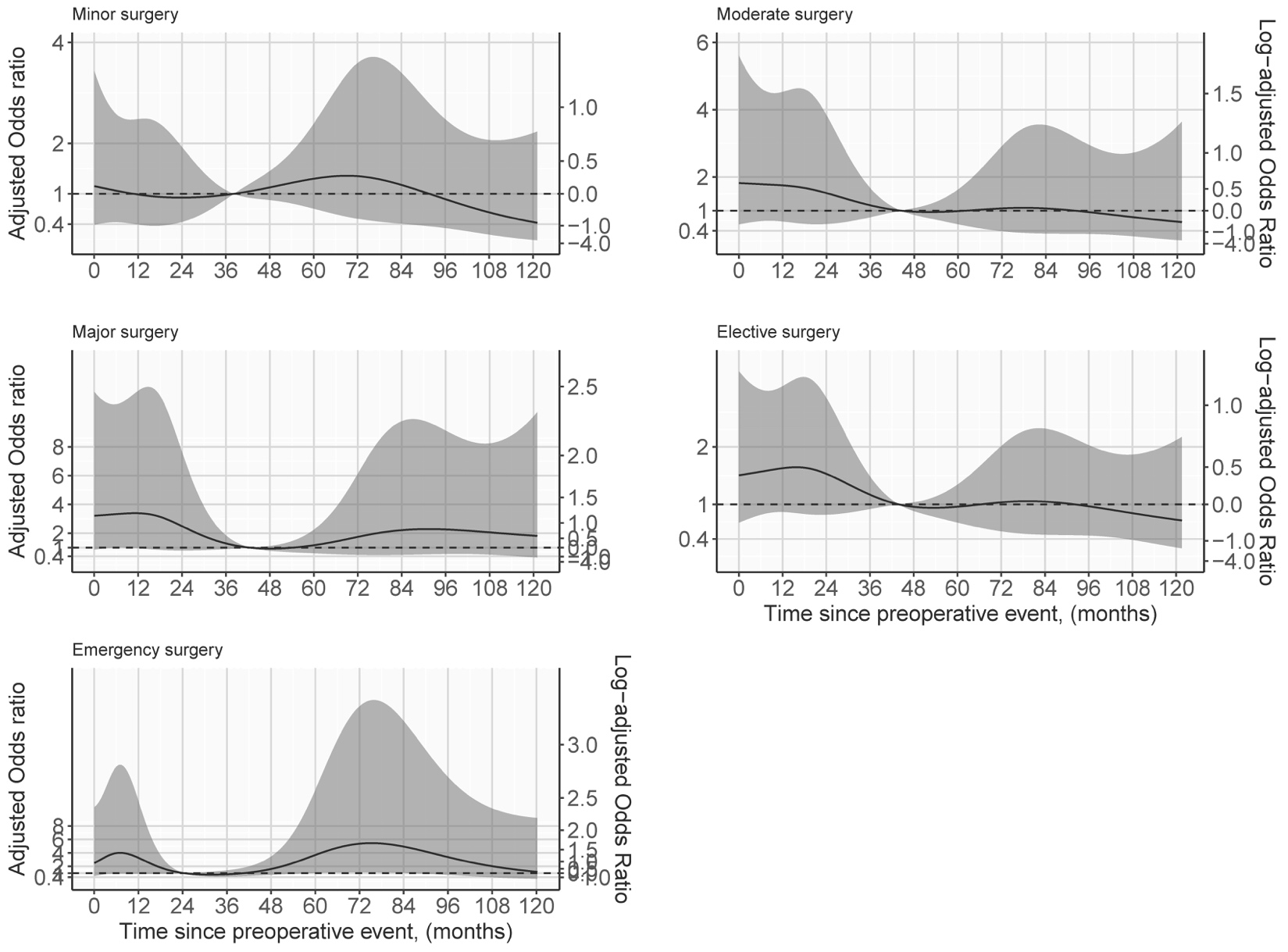


**Figure 8**. Restricted cubic splines logistic regression for 1-year risk of Acute Myocardial infarction after surgery by the time between the most recent cardiovascular event stratified by surgery invasiveness and urgency. The spline was adjusted for age, sex, index of multiple deprivation, hypertension, atrial fibrillation, stable angina, peripheral vascular disease, valvular heart disease, congestive heart failure, respiratory diseases, diabetes mellitus, renal failure, cancer, liver disease, and dementia. The median time between the event and surgery served as the reference.


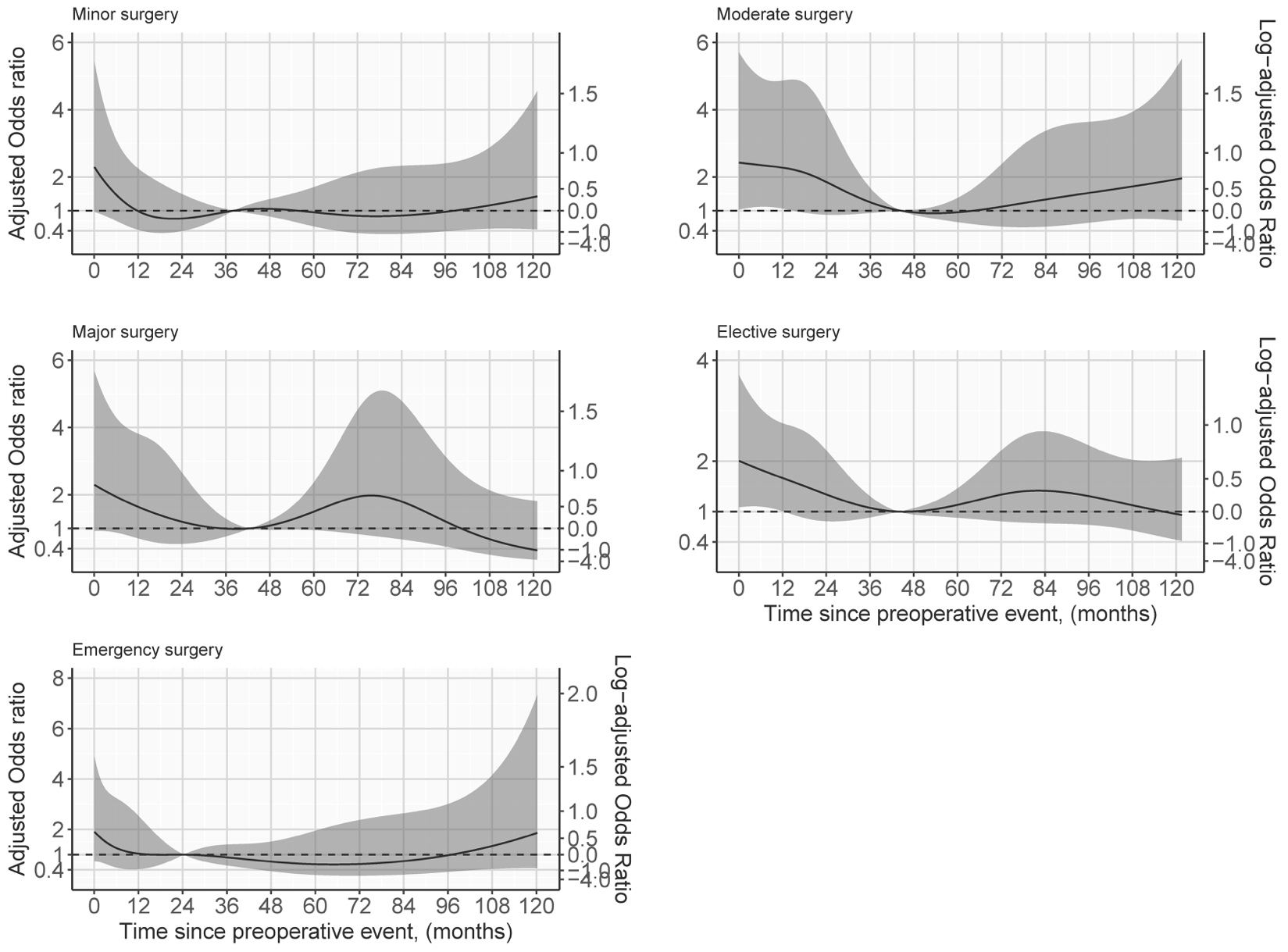


**Figure 9.** Restricted cubic splines logistic regression for 1-year risk of cerebrovascular accident after surgery by the time between the most recent cardiovascular event stratified by surgery invasiveness and urgency. The spline was adjusted for age, sex, index of multiple deprivation, hypertension, atrial fibrillation, stable angina, peripheral vascular disease, valvular heart disease, congestive heart failure, respiratory diseases, diabetes mellitus, renal failure, cancer, liver disease, and dementia. The median time between the event and surgery served as the reference.

### Ophthalmological surgery


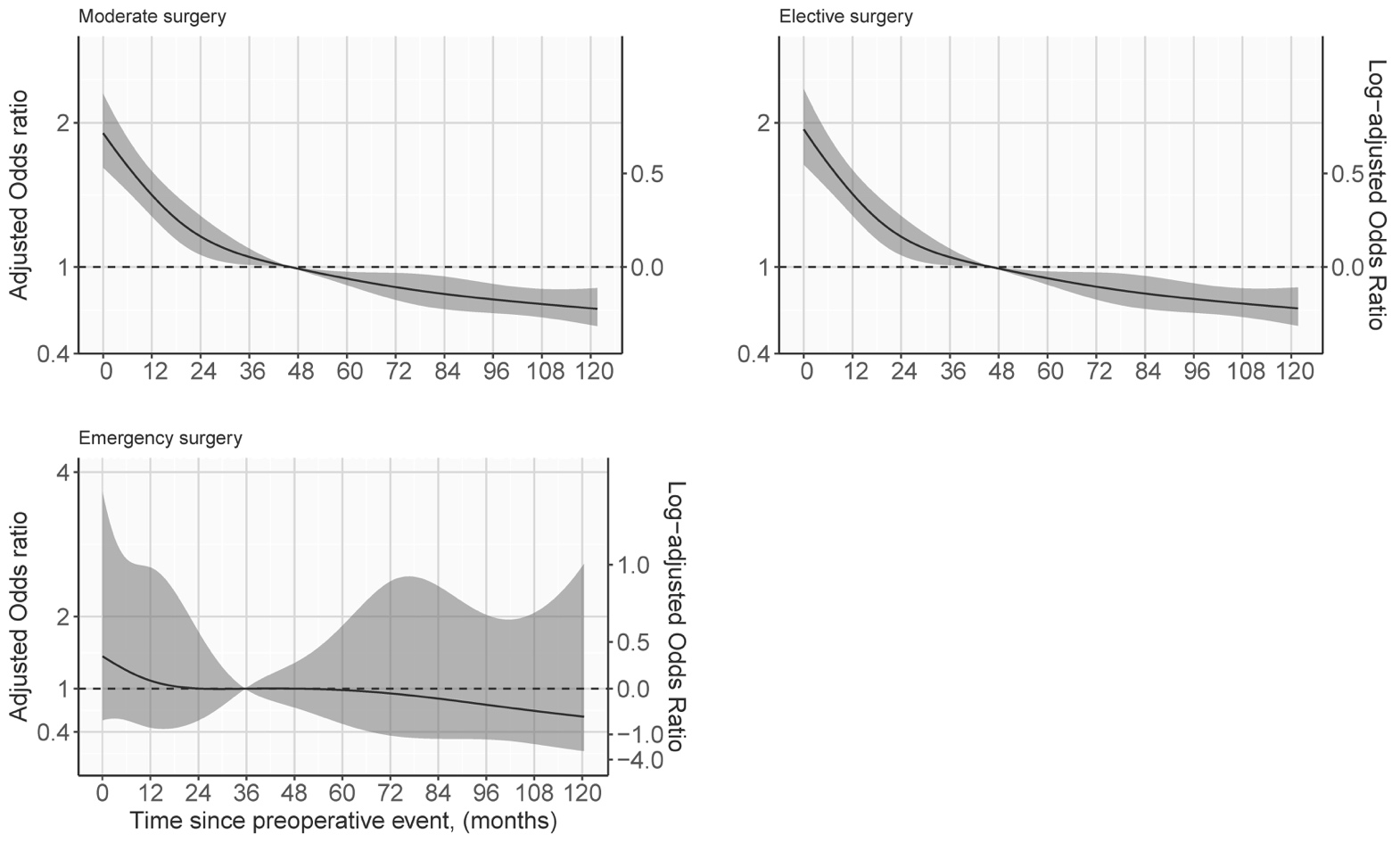


**Figure 10.** Restricted cubic splines logistic regression for 1-year risk of Acute Coronary Syndrome after surgery by the time between the most recent cardiovascular event stratified by surgery invasiveness and urgency. The spline was adjusted for age, sex, index of multiple deprivation, hypertension, atrial fibrillation, stable angina, peripheral vascular disease, valvular heart disease, congestive heart failure, respiratory diseases, diabetes mellitus, renal failure, cancer, liver disease, and dementia. The median time between the event and surgery served as the reference.


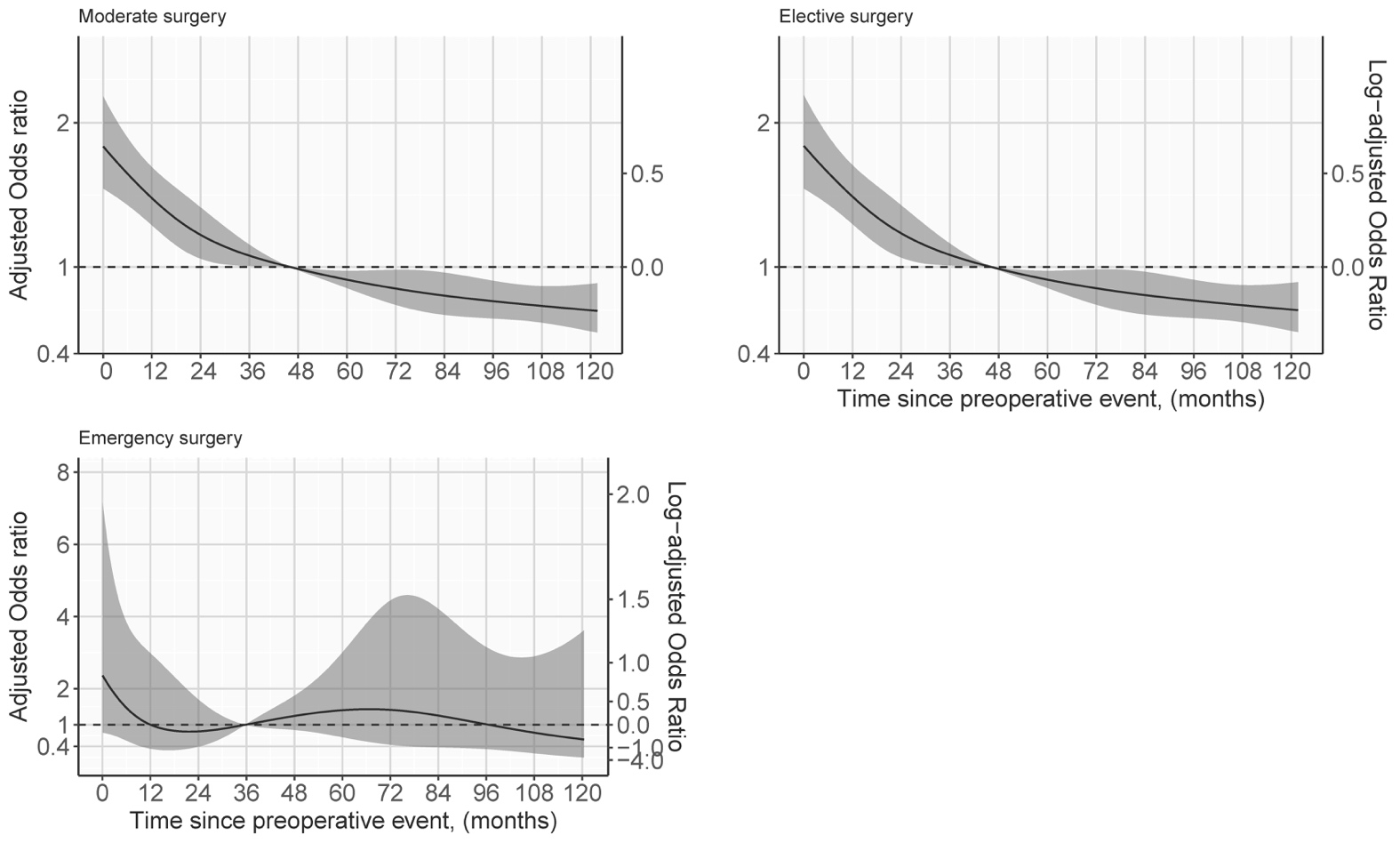


**Figure 11**. Restricted cubic splines logistic regression for 1-year risk of Acute Myocardial infarction after surgery by the time between the most recent cardiovascular event stratified by surgery invasiveness and urgency. The spline was adjusted for age, sex, index of multiple deprivation, hypertension, atrial fibrillation, stable angina, peripheral vascular disease, valvular heart disease, congestive heart failure, respiratory diseases, diabetes mellitus, renal failure, cancer, liver disease, and dementia. The median time between the event and surgery served as the reference.


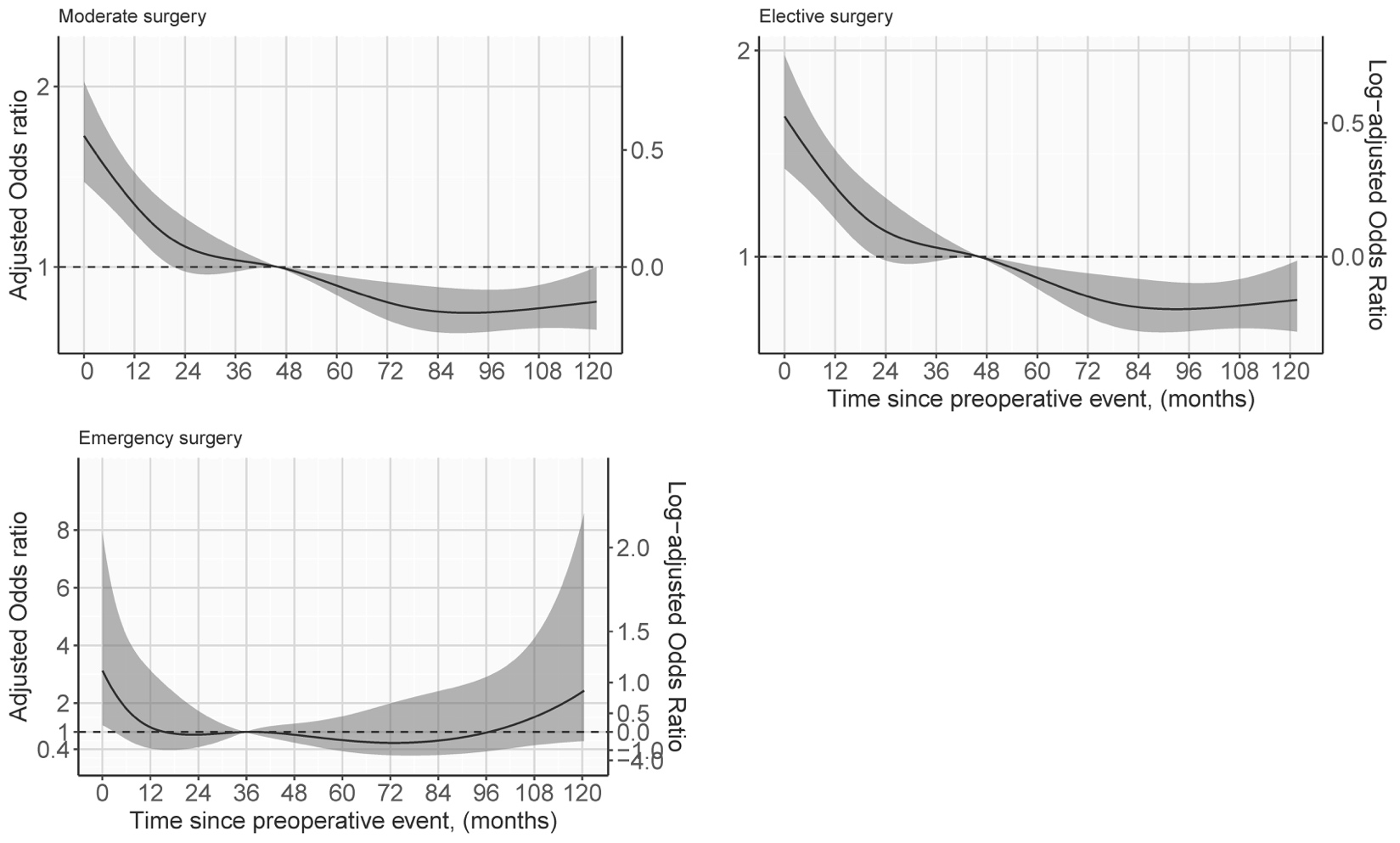


**Figure 12**. Restricted cubic splines logistic regression for 1-year risk of cerebrovascular accident after surgery by the time between the most recent cardiovascular event stratified by surgery invasiveness and urgency. The spline was adjusted for age, sex, index of multiple deprivation, hypertension, atrial fibrillation, stable angina, peripheral vascular disease, valvular heart disease, congestive heart failure, respiratory diseases, diabetes mellitus, renal failure, cancer, liver disease, and dementia. The median time between the event and surgery served as the reference.

### Otolaryngology surgery


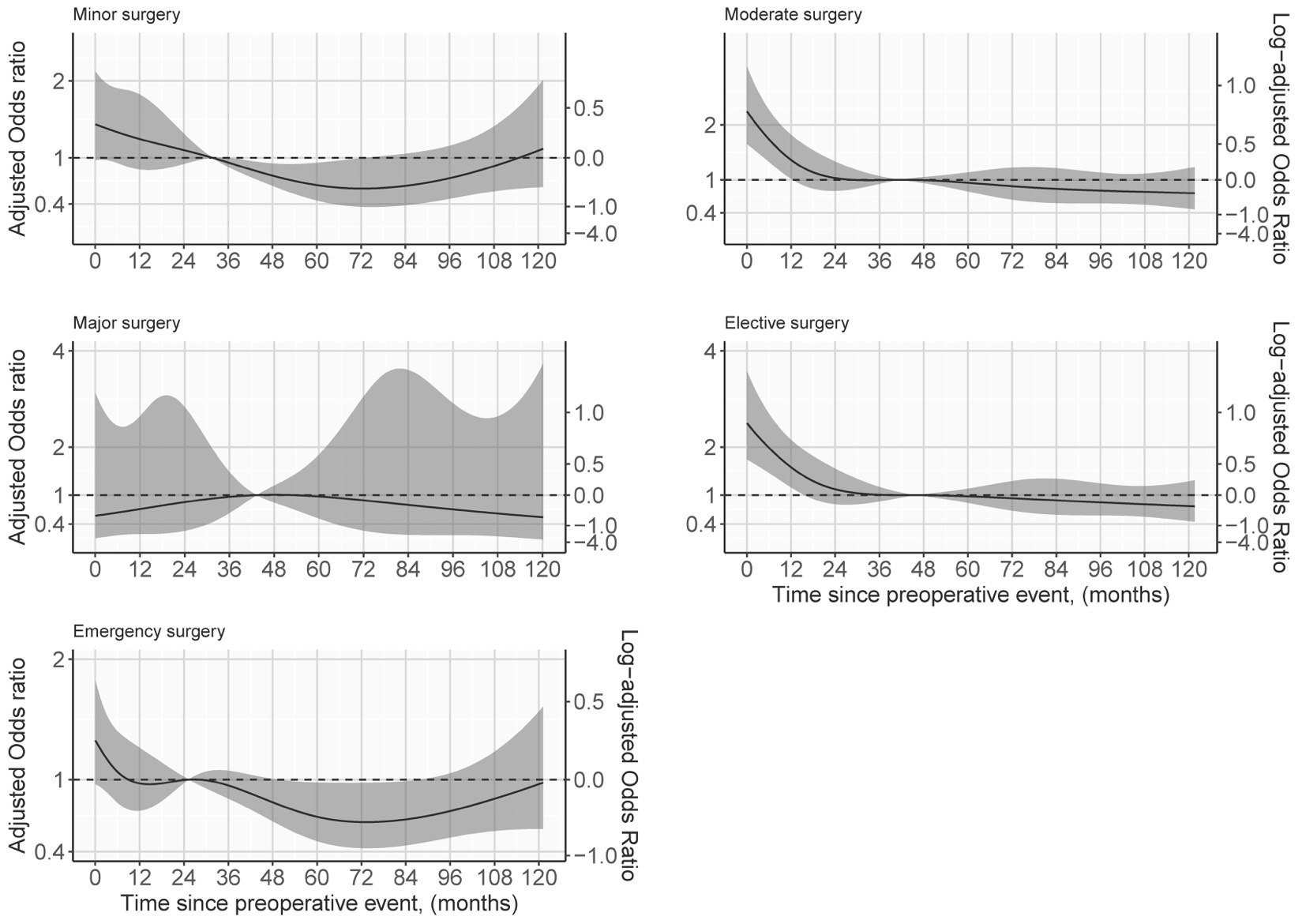


**Figure 13.** Restricted cubic splines logistic regression for 1-year risk of Acute Coronary Syndrome after surgery by the time between the most recent cardiovascular event stratified by surgery invasiveness and urgency. The spline was adjusted for age, sex, index of multiple deprivation, hypertension, atrial fibrillation, stable angina, peripheral vascular disease, valvular heart disease, congestive heart failure, respiratory diseases, diabetes mellitus, renal failure, cancer, liver disease, and dementia. The median time between the event and surgery served as the reference.


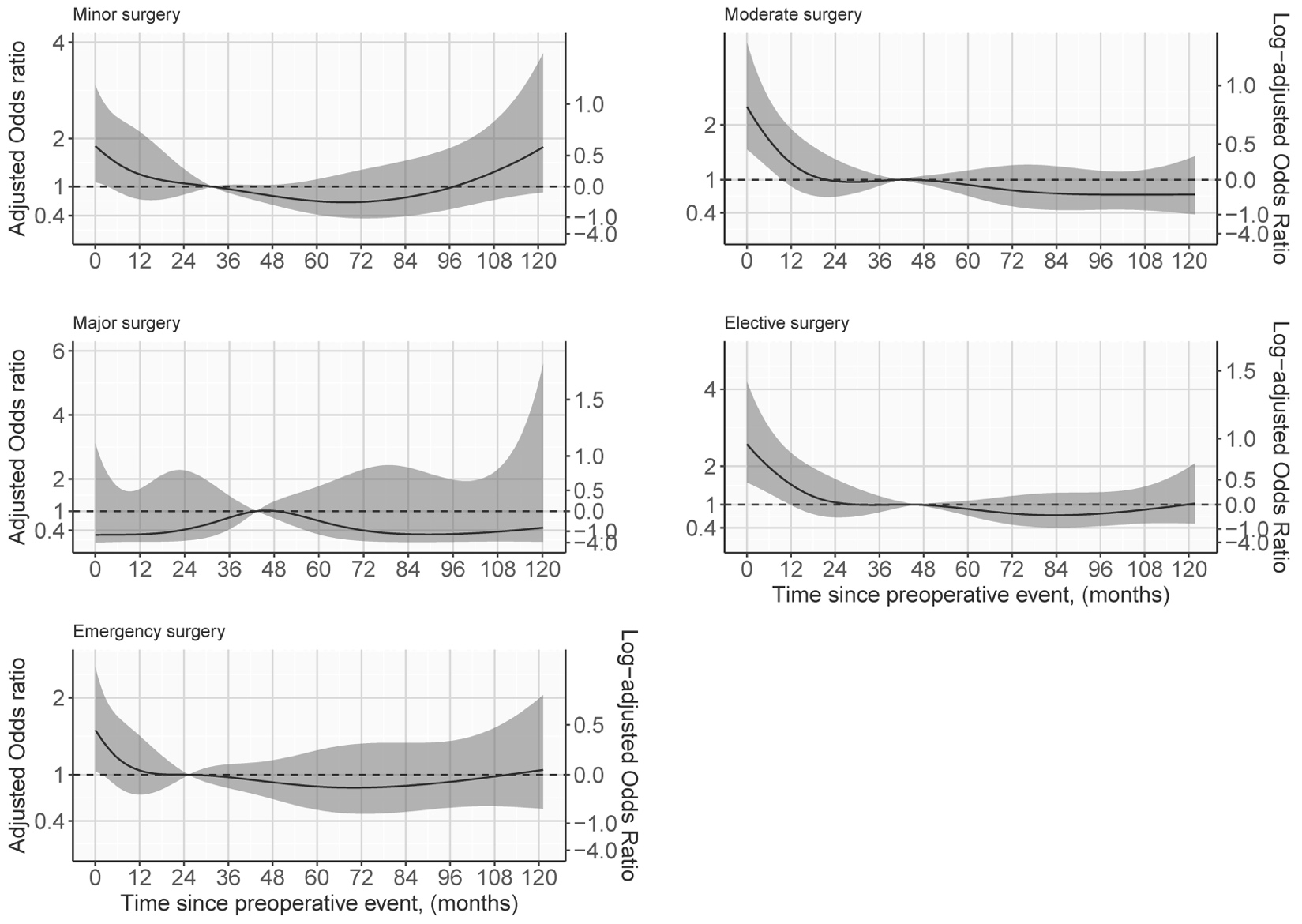


**Figure 14**. Restricted cubic splines logistic regression for 1-year risk of Acute Myocardial infarction after surgery by the time between the most recent cardiovascular event stratified by surgery invasiveness and urgency. The spline was adjusted for age, sex, index of multiple deprivation, hypertension, atrial fibrillation, stable angina, peripheral vascular disease, valvular heart disease, congestive heart failure, respiratory diseases, diabetes mellitus, renal failure, cancer, liver disease, and dementia. The median time between the event and surgery served as the reference.


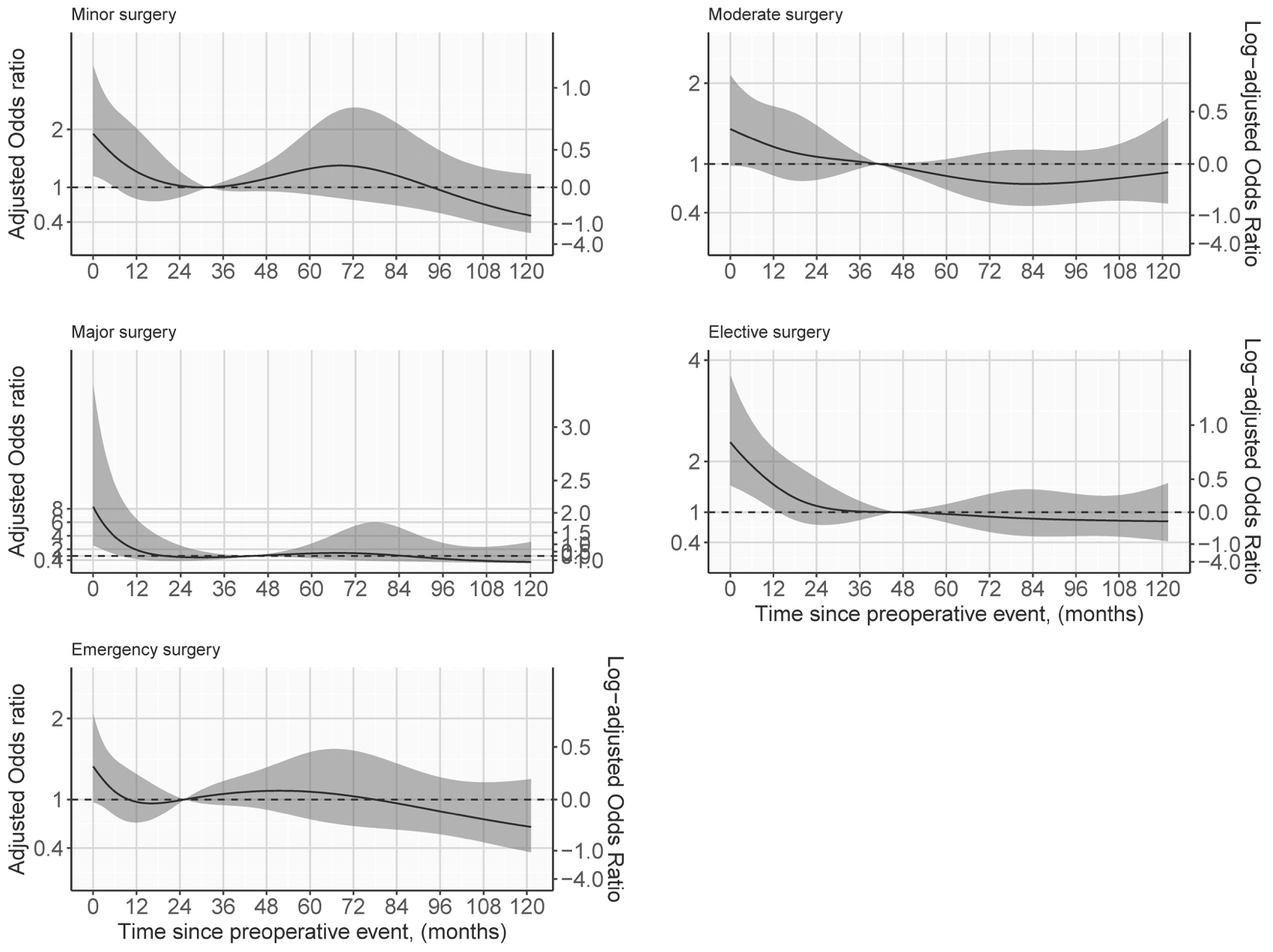


**Figure 15.** Restricted cubic splines logistic regression for 1-year risk of cerebrovascular accident after surgery by the time between the most recent cardiovascular event stratified by surgery invasiveness and urgency. The spline was adjusted for age, sex, index of multiple deprivation, hypertension, atrial fibrillation, stable angina, peripheral vascular disease, valvular heart disease, congestive heart failure, respiratory diseases, diabetes mellitus, renal failure, cancer, liver disease, and dementia. The median time between the event and surgery served as the reference.

### Orthopaedic surgery


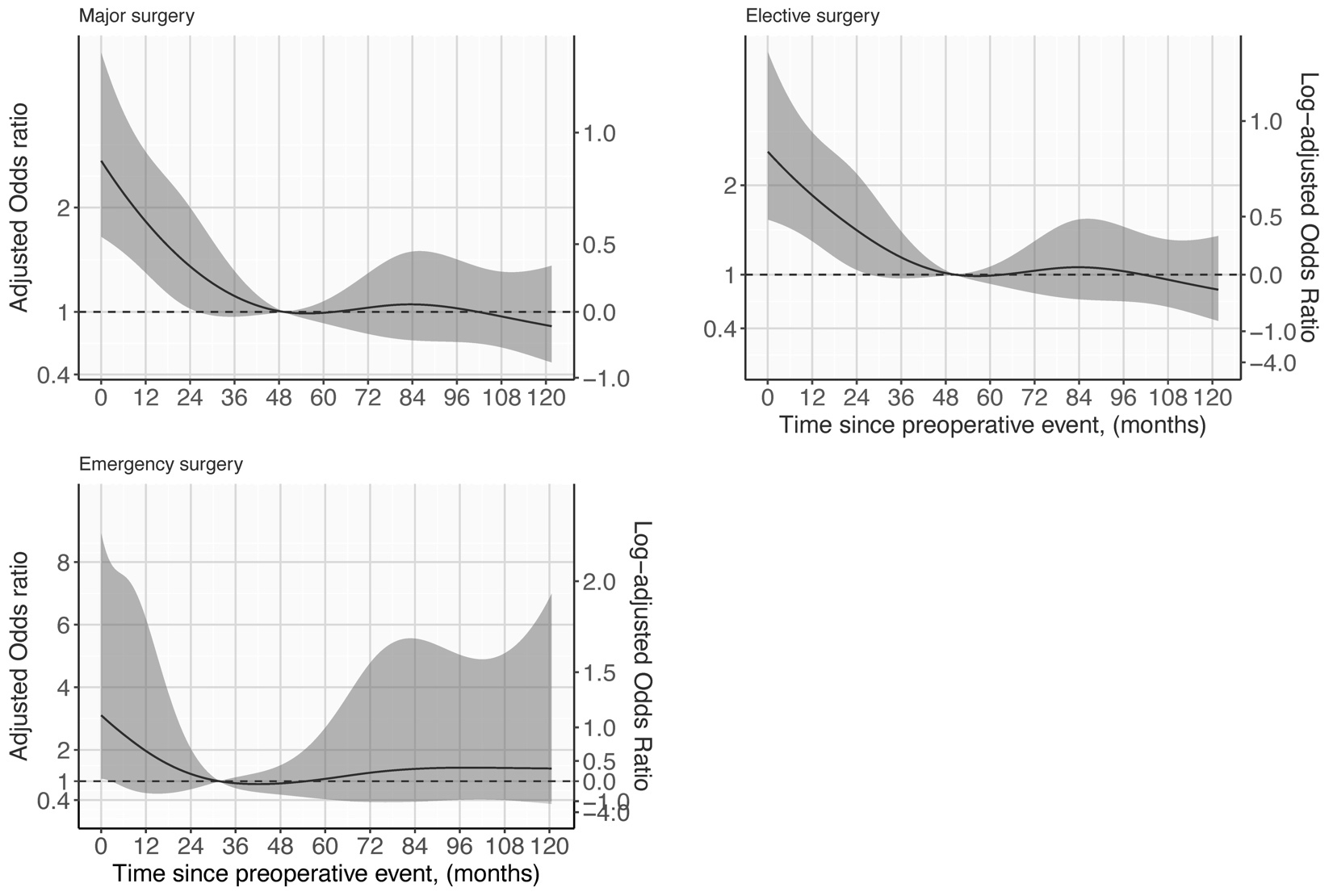


**Figure 16**. Restricted cubic splines logistic regression for 1-year risk of Acute Coronary Syndrome after surgery by the time between the most recent cardiovascular event stratified by surgery invasiveness and urgency. The spline was adjusted for age, sex, index of multiple deprivation, hypertension, atrial fibrillation, stable angina, peripheral vascular disease, valvular heart disease, congestive heart failure, respiratory diseases, diabetes mellitus, renal failure, cancer, liver disease, and dementia. The median time between the event and surgery served as the reference.


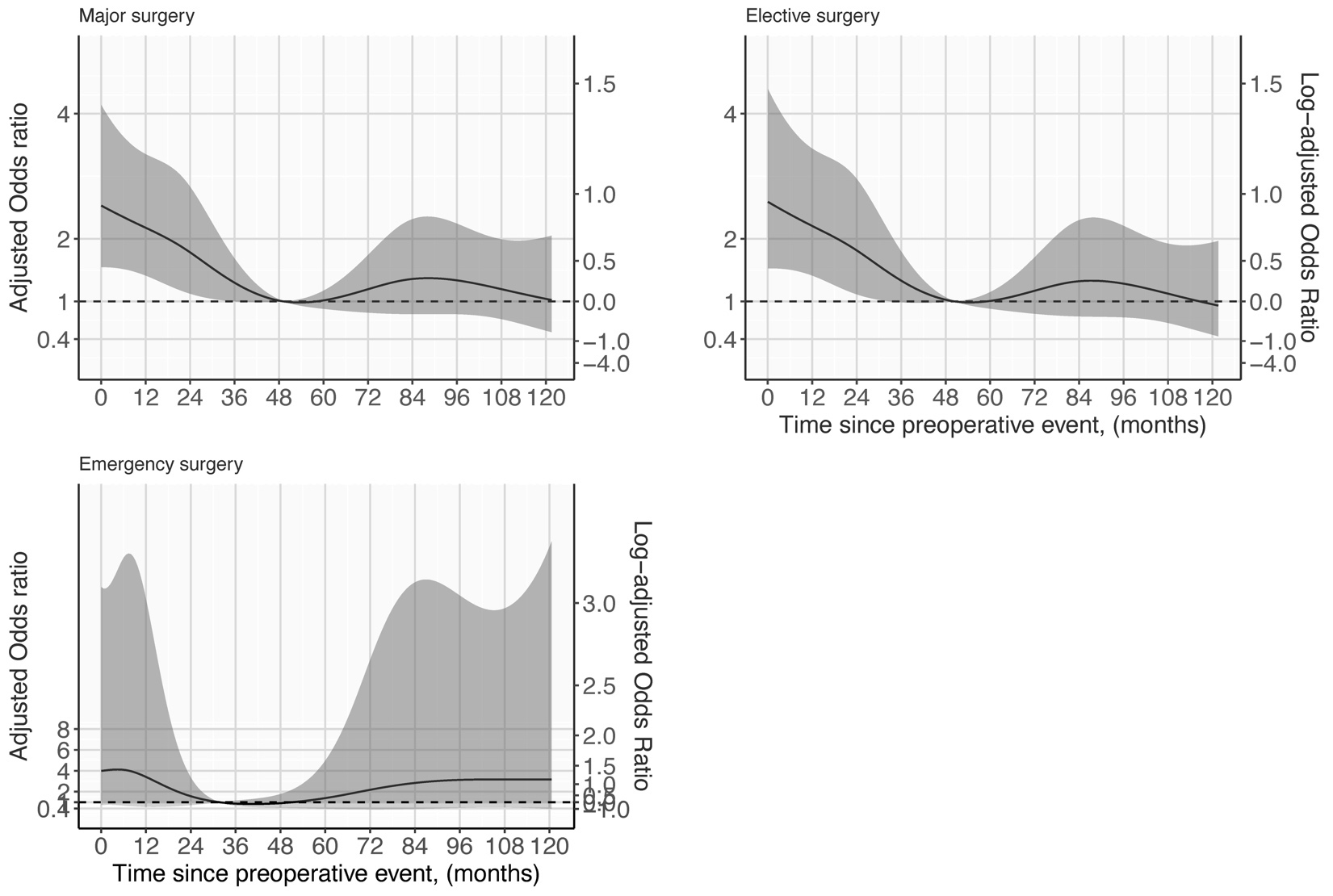


**Figure 17**. Restricted cubic splines logistic regression for 1-year risk of Acute Myocardial infarction after surgery by the time between the most recent cardiovascular event stratified by surgery invasiveness and urgency. The spline was adjusted for age, sex, index of multiple deprivation, hypertension, atrial fibrillation, stable angina, peripheral vascular disease, valvular heart disease, congestive heart failure, respiratory diseases, diabetes mellitus, renal failure, cancer, liver disease, and dementia. The median time between the event and surgery served as the reference.


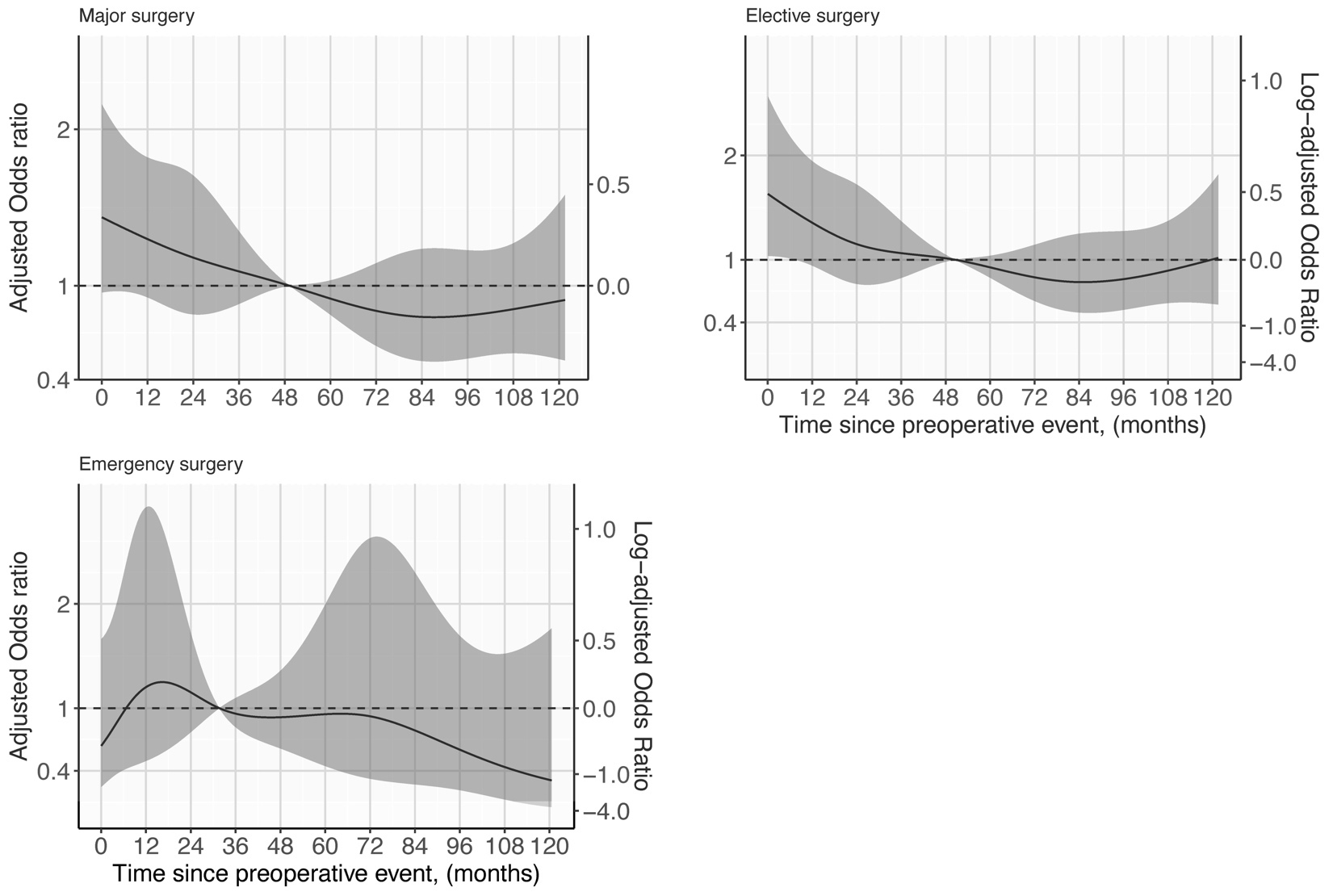


**Figure 18**. Restricted cubic splines logistic regression for 1-year risk of cerebrovascular accident after surgery by the time between the most recent cardiovascular event stratified by surgery invasiveness and urgency. The spline was adjusted for age, sex, index of multiple deprivation, hypertension, atrial fibrillation, stable angina, peripheral vascular disease, valvular heart disease, congestive heart failure, respiratory diseases, diabetes mellitus, renal failure, cancer, liver disease, and dementia. The median time between the event and surgery served as the reference.

#### Adverse outcomes by subtype of orthopaedic surgery

##### Primary hip


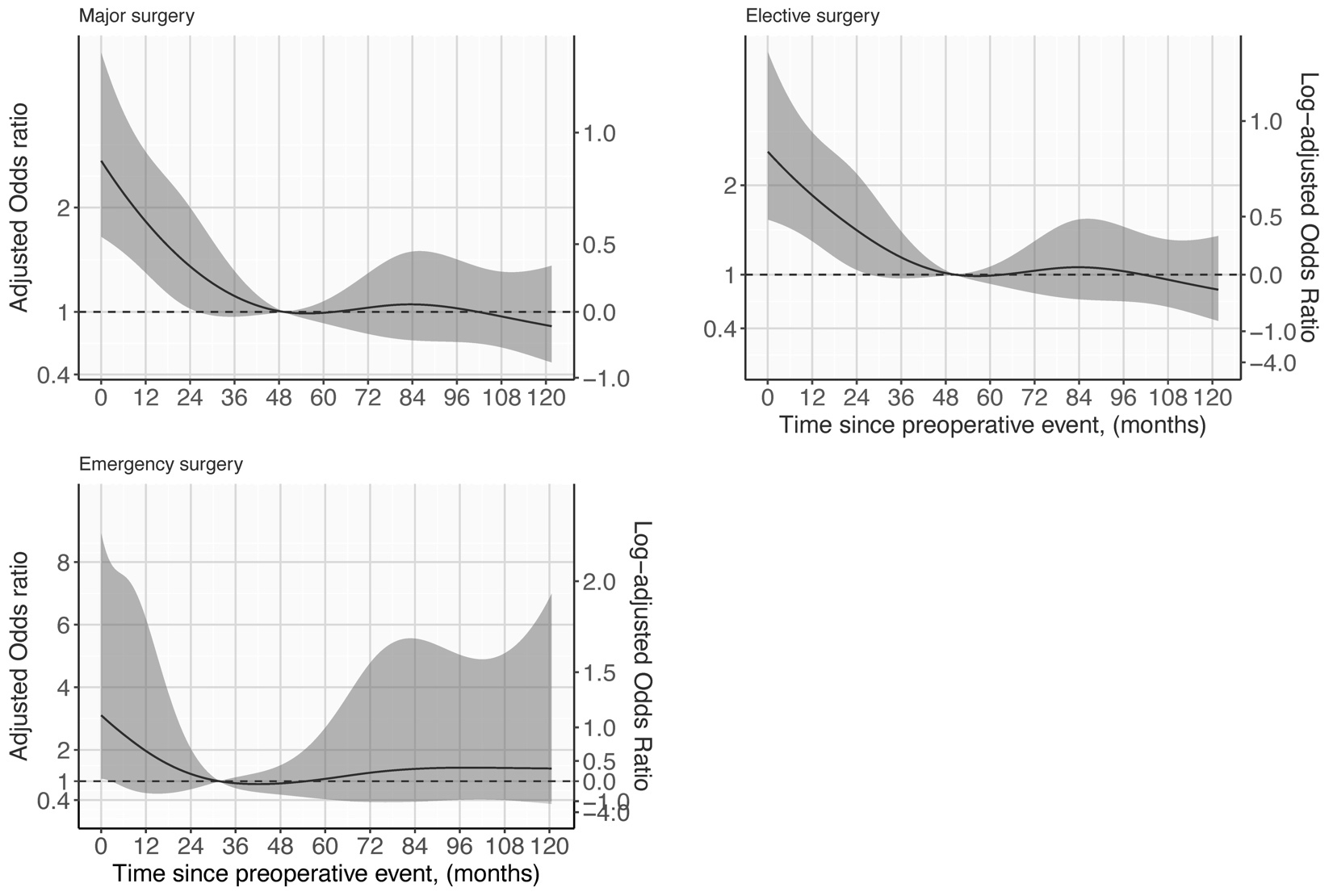


**Figure 19**. Restricted cubic splines logistic regression for 1-year risk of Acute Coronary Syndrome after surgery by the time between the most recent cardiovascular event stratified by surgery invasiveness and urgency. The spline was adjusted for age, sex, index of multiple deprivation, hypertension, atrial fibrillation, stable angina, peripheral vascular disease, valvular heart disease, congestive heart failure, respiratory diseases, diabetes mellitus, renal failure, cancer, liver disease, and dementia. The median time between the event and surgery served as the reference.


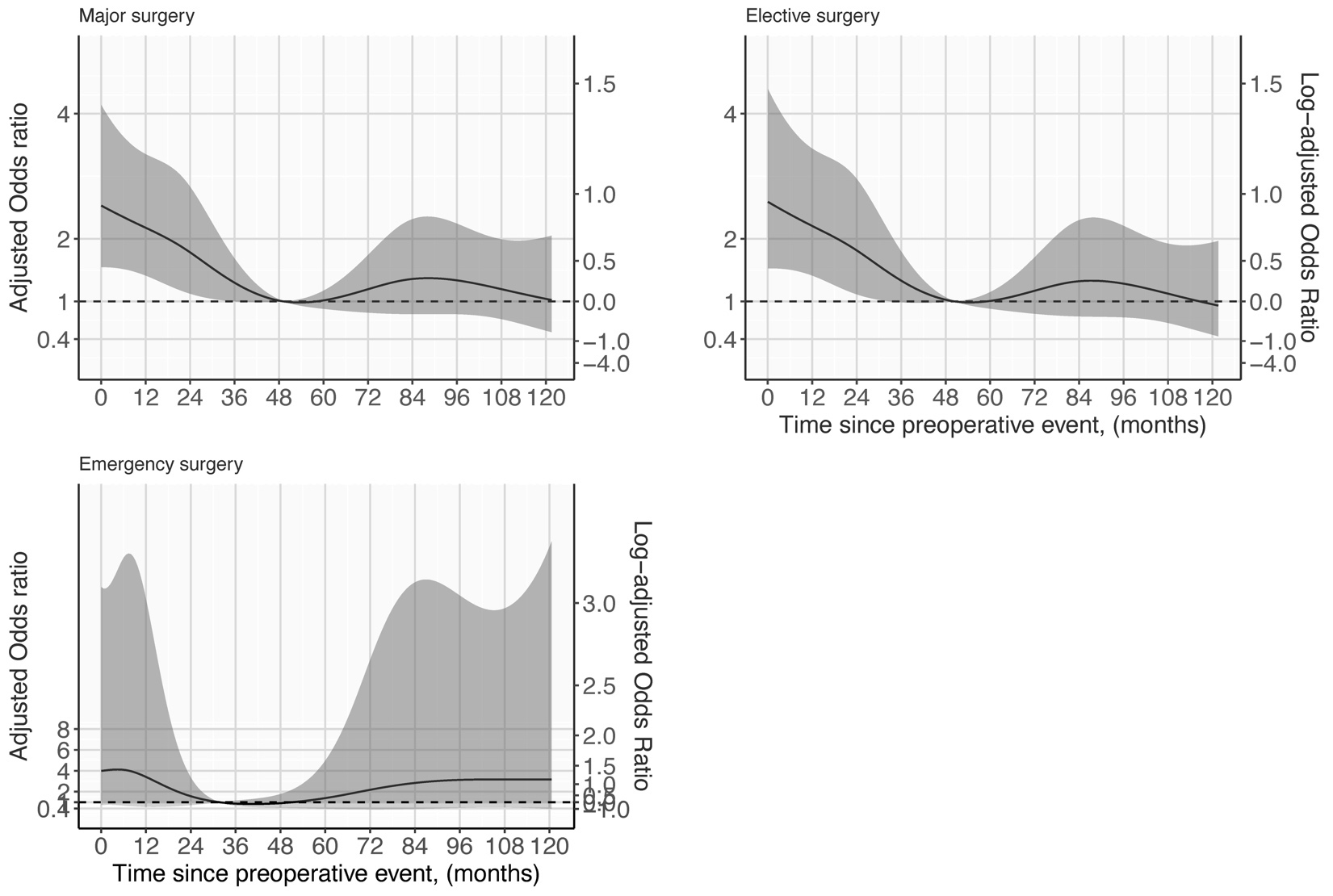


**Figure 20**. Restricted cubic splines logistic regression for 1-year risk of Acute Myocardial infarction after surgery by the time between the most recent cardiovascular event stratified by surgery invasiveness and urgency. The spline was adjusted for age, sex, index of multiple deprivation, hypertension, atrial fibrillation, stable angina, peripheral vascular disease, valvular heart disease, congestive heart failure, respiratory diseases, diabetes mellitus, renal failure, cancer, liver disease, and dementia. The median time between the event and surgery served as the reference.


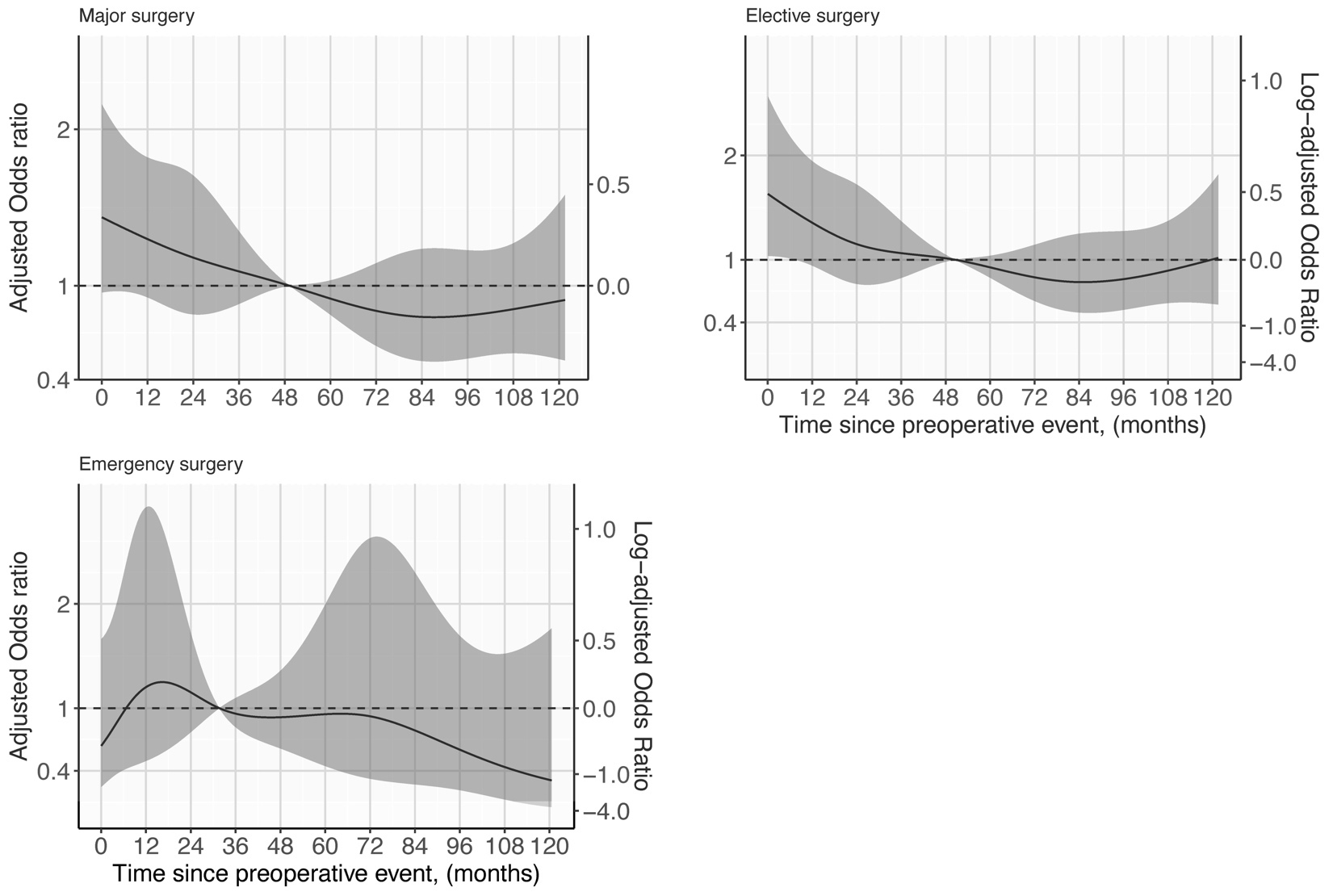


**Figure 21**. Restricted cubic splines logistic regression for 1-year risk of cerebrovascular accident after surgery by the time between the most recent cardiovascular event stratified by surgery invasiveness and urgency. The spline was adjusted for age, sex, index of multiple deprivation, hypertension, atrial fibrillation, stable angina, peripheral vascular disease, valvular heart disease, congestive heart failure, respiratory diseases, diabetes mellitus, renal failure, cancer, liver disease, and dementia. The median time between the event and surgery served as the reference.

##### Revision conversion hip


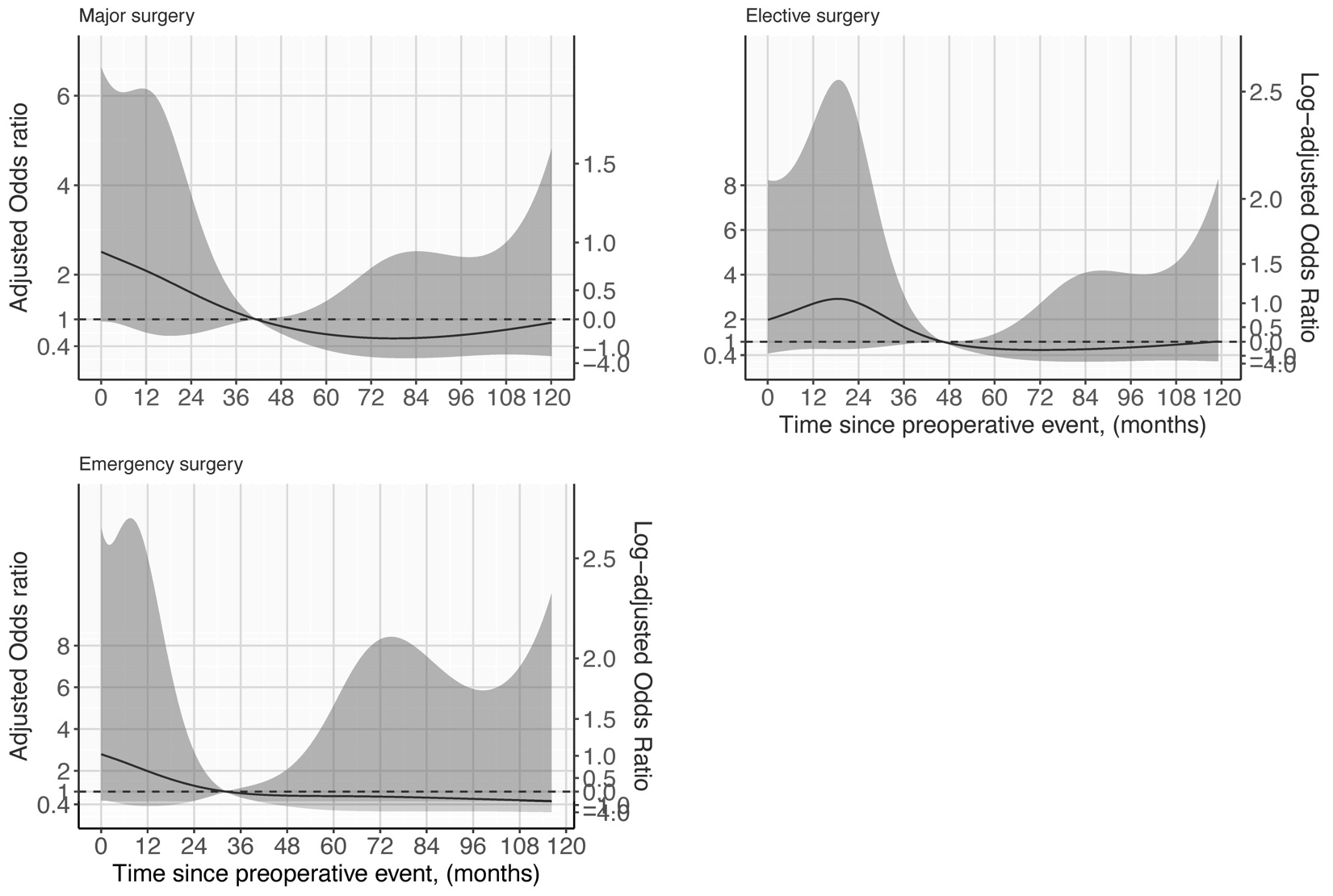


**Figure 22**. Restricted cubic splines logistic regression for 1-year risk of Acute Coronary Syndrome after surgery by the time between the most recent cardiovascular event stratified by surgery invasiveness and urgency. The spline was adjusted for age, sex, index of multiple deprivation, hypertension, atrial fibrillation, stable angina, peripheral vascular disease, valvular heart disease, congestive heart failure, respiratory diseases, diabetes mellitus, renal failure, cancer, liver disease, and dementia. The median time between the event and surgery served as the reference.


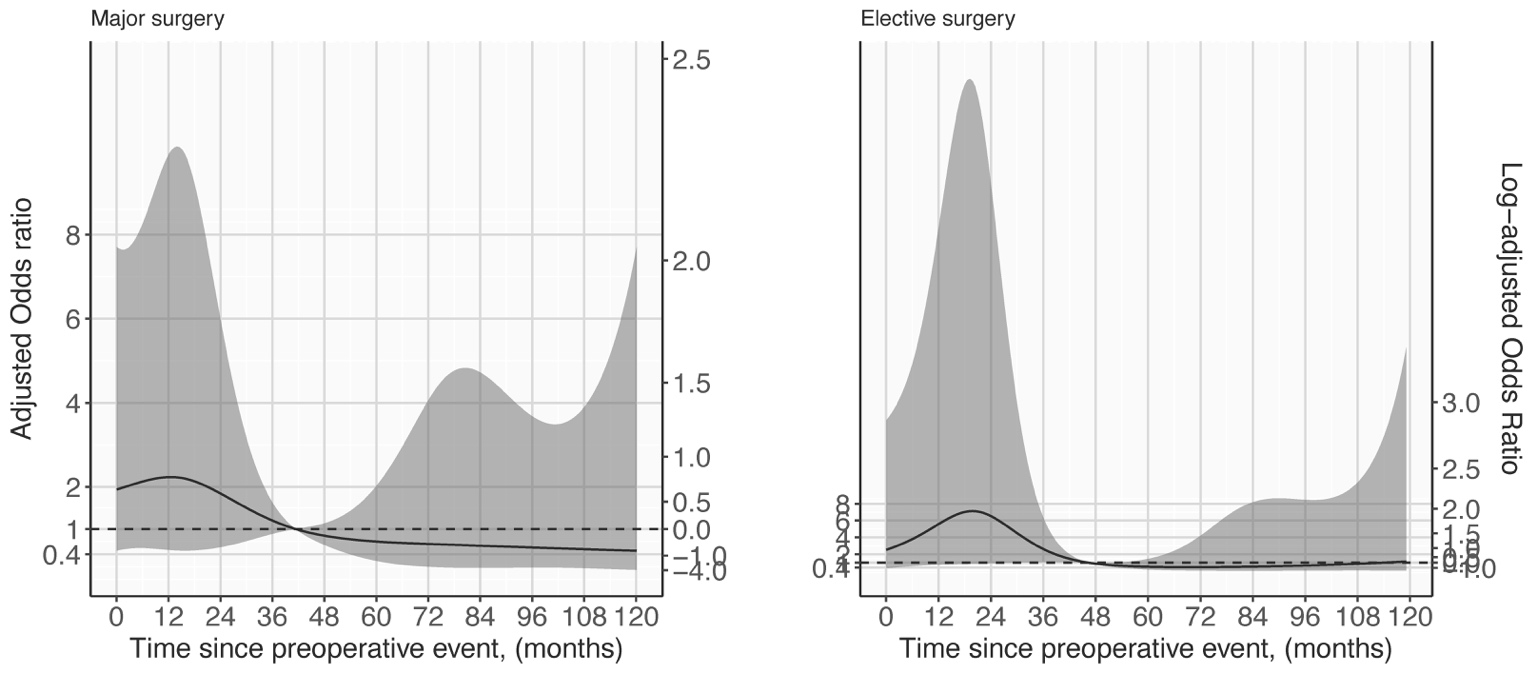


**Figure 23**. Restricted cubic splines logistic regression for 1-year risk of Acute Myocardial infarction after surgery by the time between the most recent cardiovascular event stratified by surgery invasiveness and urgency. The spline was adjusted for age, sex, index of multiple deprivation, hypertension, atrial fibrillation, stable angina, peripheral vascular disease, valvular heart disease, congestive heart failure, respiratory diseases, diabetes mellitus, renal failure, cancer, liver disease, and dementia. The median time between the event and surgery served as the reference.


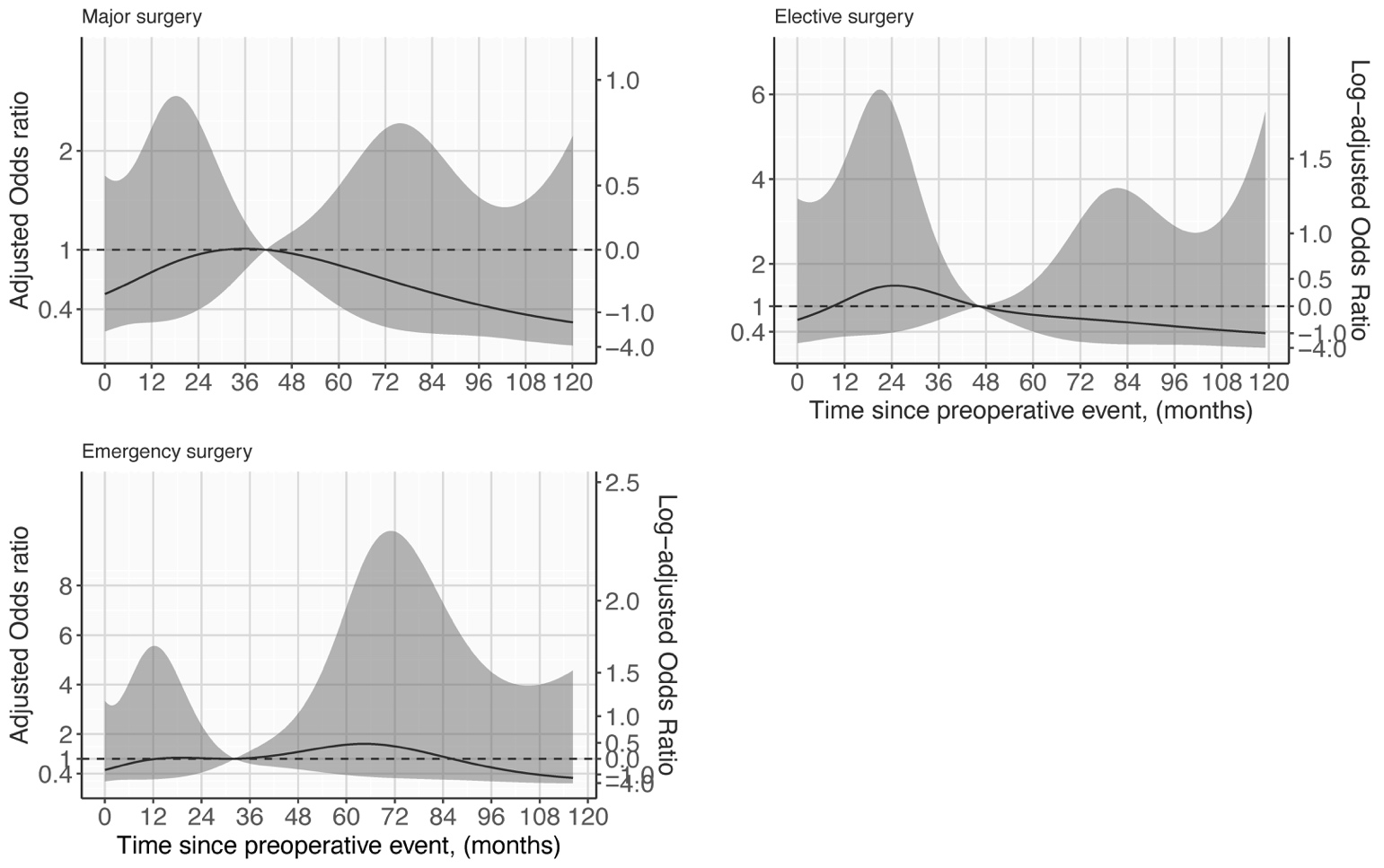


**Figure 24**. Restricted cubic splines logistic regression for 1-year risk of cerebrovascular accident after surgery by the time between the most recent cardiovascular event stratified by surgery invasiveness and urgency. The spline was adjusted for age, sex, index of multiple deprivation, hypertension, atrial fibrillation, stable angina, peripheral vascular disease, valvular heart disease, congestive heart failure, respiratory diseases, diabetes mellitus, renal failure, cancer, liver disease, and dementia. The median time between the event and surgery served as the reference.

### Vascular surgery


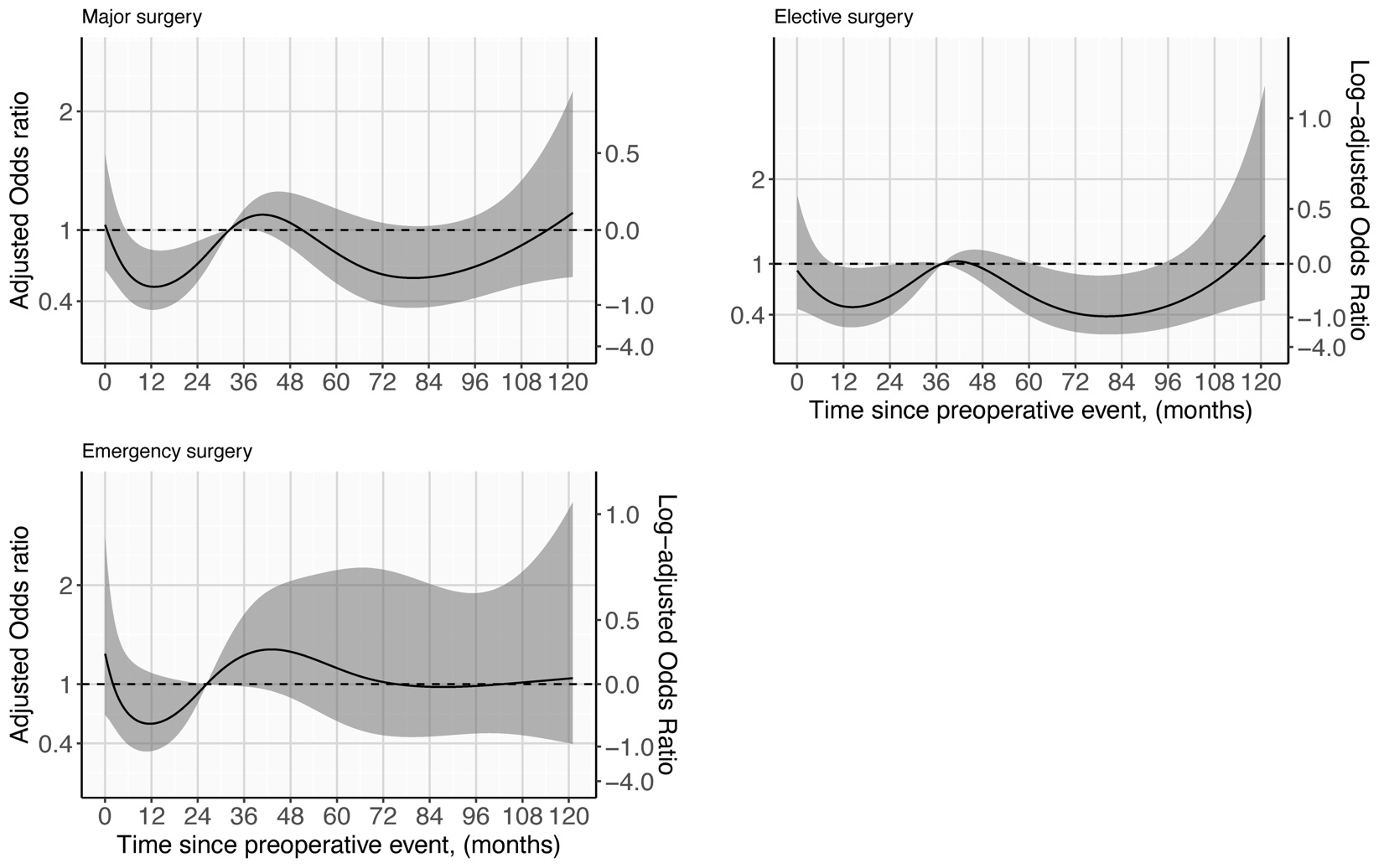


**Figure 25**. Restricted cubic splines logistic regression for 1-year risk of Acute Coronary Syndrome after surgery by the time between the most recent cardiovascular event stratified by surgery invasiveness and urgency. The spline was adjusted for age, sex, index of multiple deprivation, hypertension, atrial fibrillation, stable angina, peripheral vascular disease, valvular heart disease, congestive heart failure, respiratory diseases, diabetes mellitus, renal failure, cancer, liver disease, and dementia. The median time between the event and surgery served as the reference.


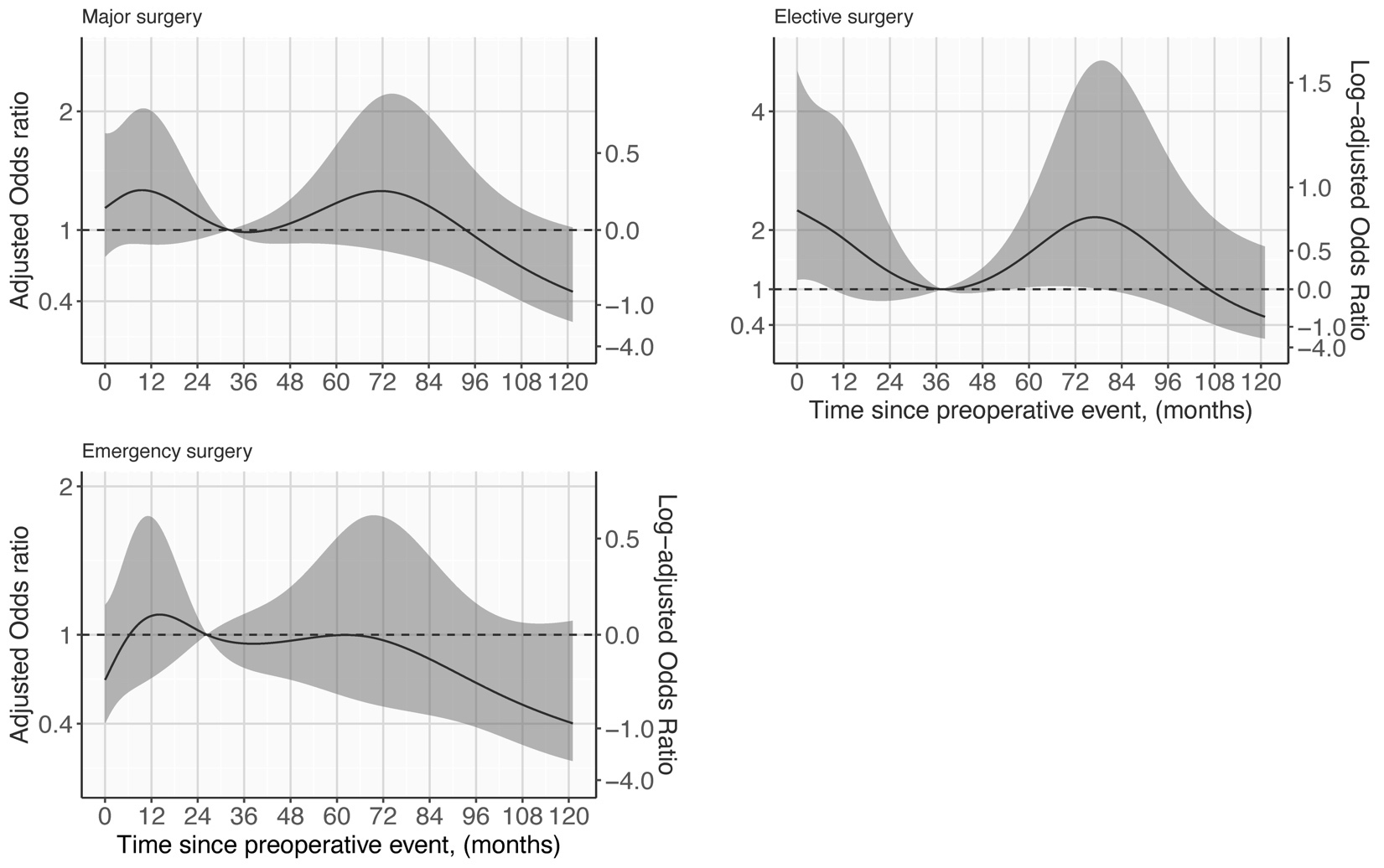


**Figure 26**. Restricted cubic splines logistic regression for 1-year risk of Acute Myocardial infarction after surgery by the time between the most recent cardiovascular event stratified by surgery invasiveness and urgency. The spline was adjusted for age, sex, index of multiple deprivation, hypertension, atrial fibrillation, stable angina, peripheral vascular disease, valvular heart disease, congestive heart failure, respiratory diseases, diabetes mellitus, renal failure, cancer, liver disease, and dementia. The median time between the event and surgery served as the reference.


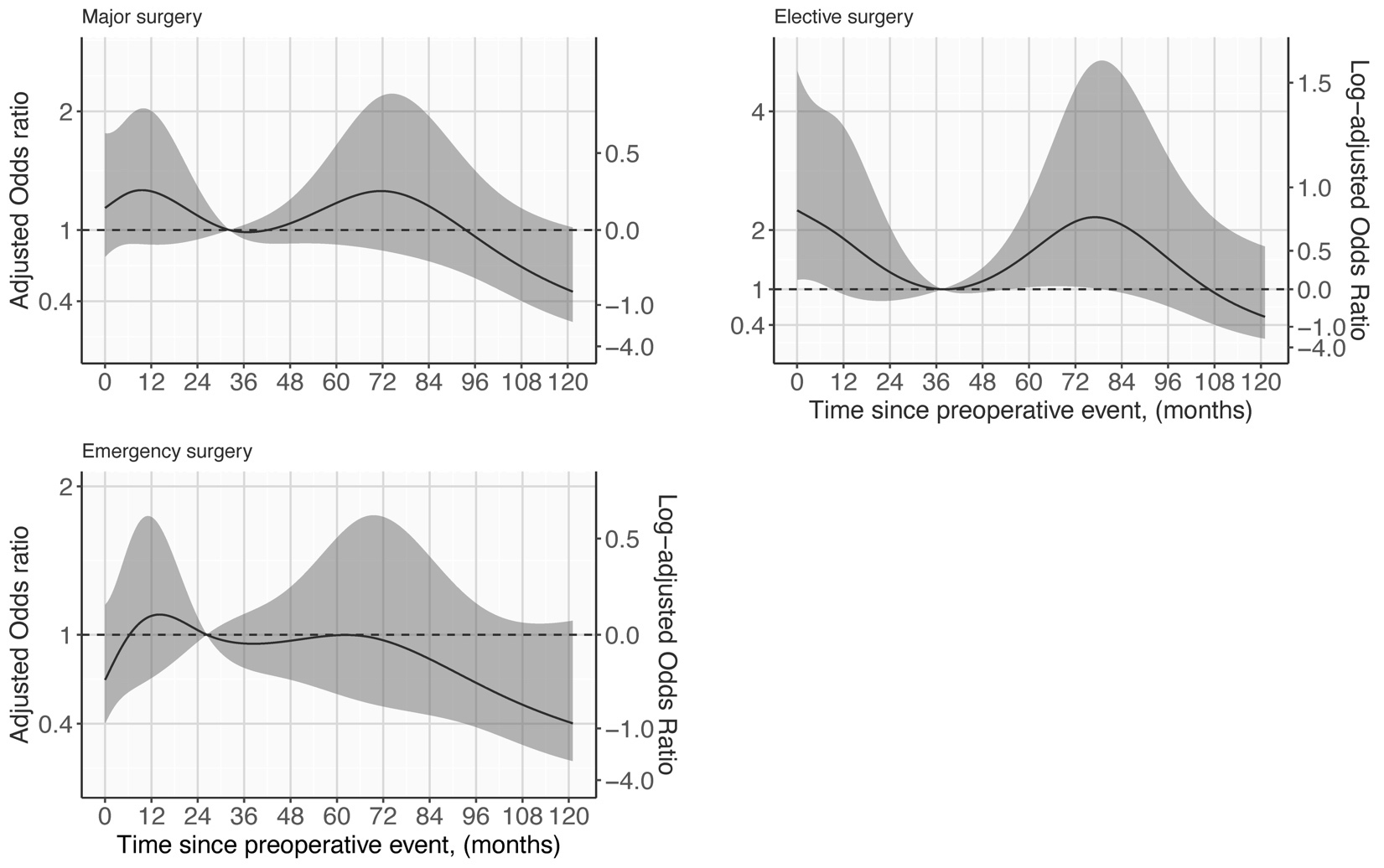


**Figure 27**. Restricted cubic splines logistic regression for 1-year risk of cerebrovascular accident after surgery by the time between the most recent cardiovascular event stratified by surgery invasiveness and urgency. The spline was adjusted for age, sex, index of multiple deprivation, hypertension, atrial fibrillation, stable angina, peripheral vascular disease, valvular heart disease, congestive heart failure, respiratory diseases, diabetes mellitus, renal failure, cancer, liver disease, and dementia. The median time between the event and surgery served as the reference.

#### Adverse outcomes by subtype of vascular surgery

##### Abdominal Aortic Aneurysm (AAA) open


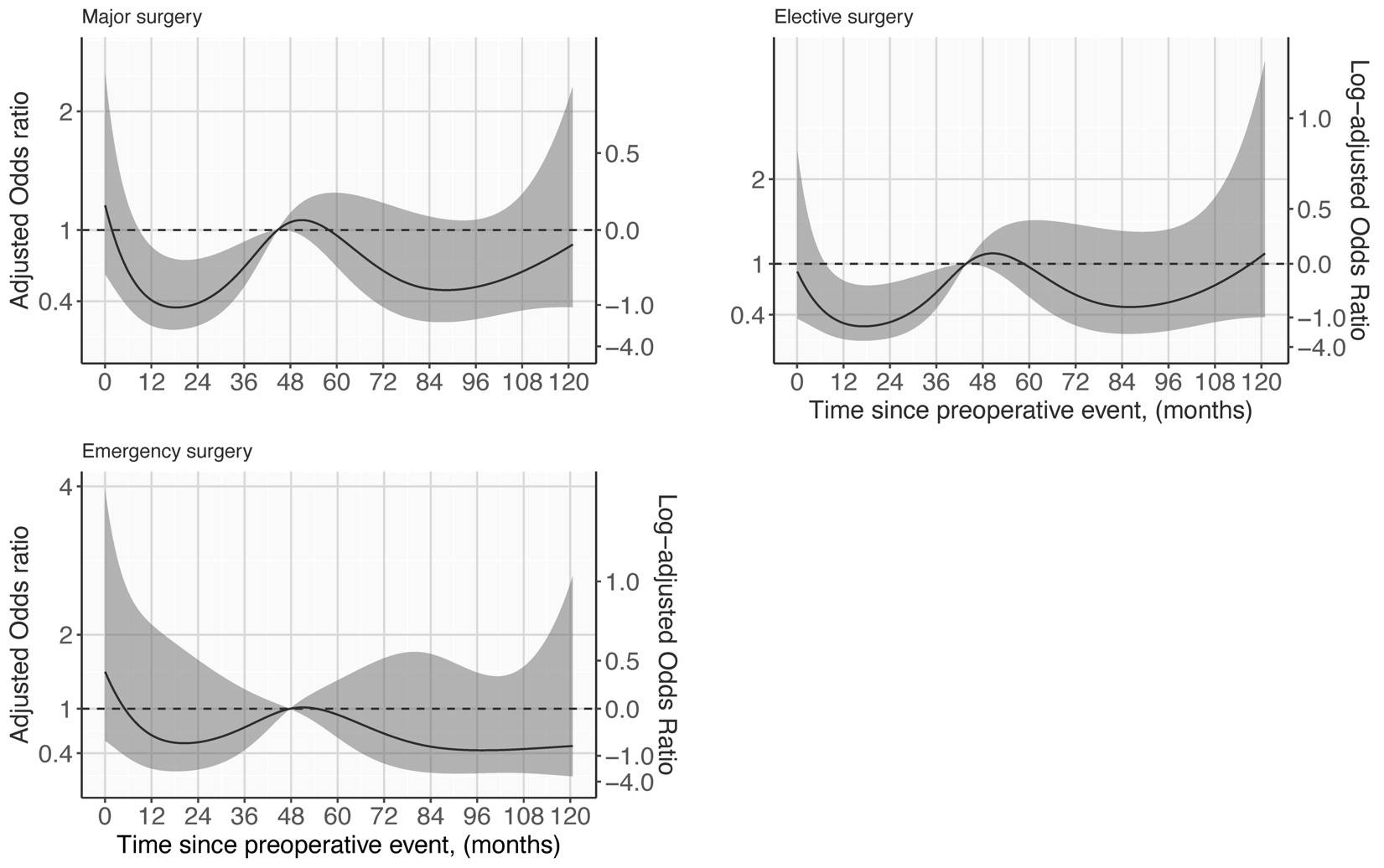


**Figure 28.** Restricted cubic splines logistic regression for 1-year risk of Acute Coronary Syndrome after surgery by the time between the most recent cardiovascular event stratified by surgery invasiveness and urgency. The spline was adjusted for age, sex, index of multiple deprivation, hypertension, atrial fibrillation, stable angina, peripheral vascular disease, valvular heart disease, congestive heart failure, respiratory diseases, diabetes mellitus, renal failure, cancer, liver disease, and dementia. The median time between the event and surgery served as the reference.


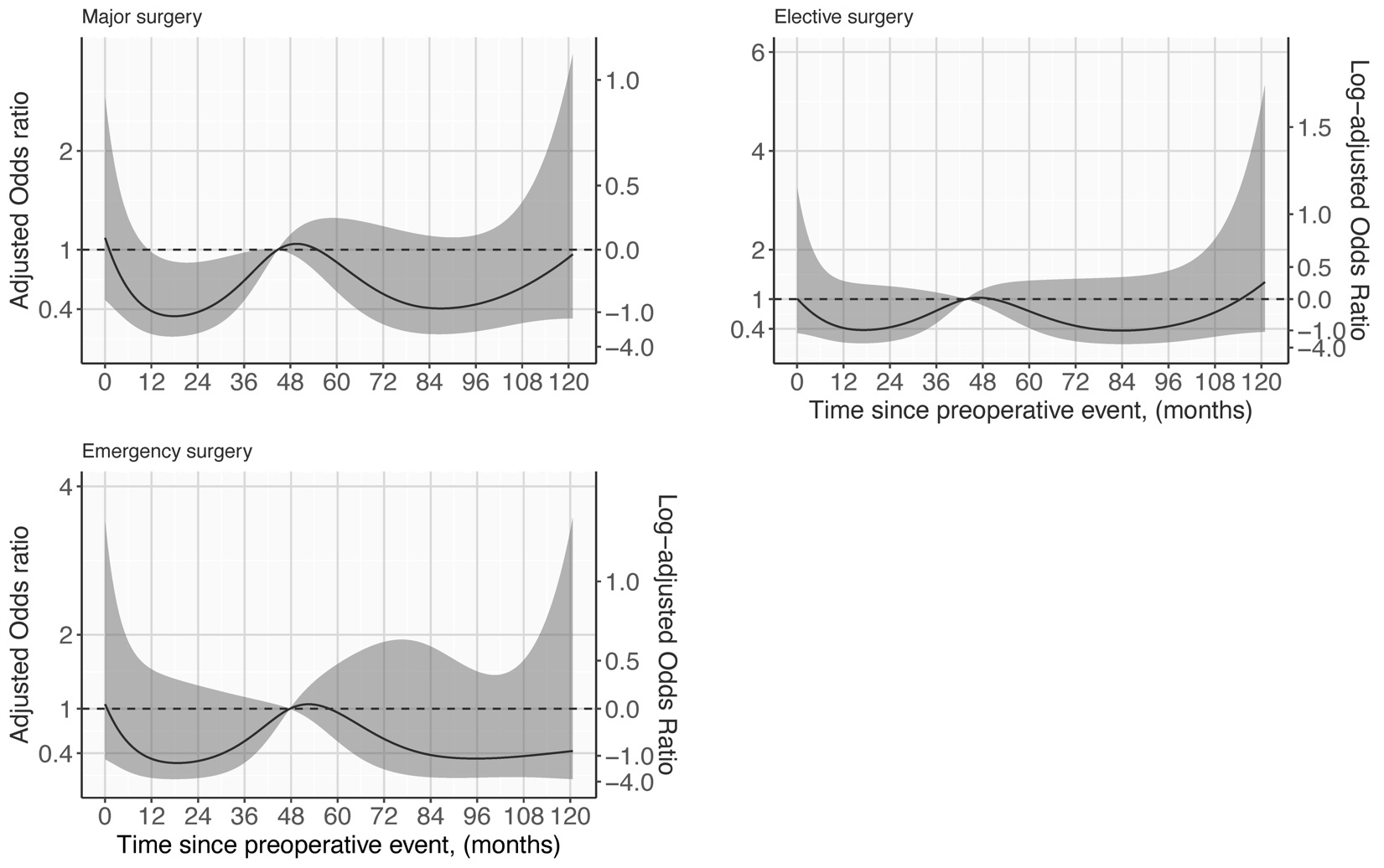


**Figure 29**. Restricted cubic splines logistic regression for 1-year risk of Acute Myocardial infarction after surgery by the time between the most recent cardiovascular event stratified by surgery invasiveness and urgency. The spline was adjusted for age, sex, index of multiple deprivation, hypertension, atrial fibrillation, stable angina, peripheral vascular disease, valvular heart disease, congestive heart failure, respiratory diseases, diabetes mellitus, renal failure, cancer, liver disease, and dementia. The median time between the event and surgery served as the reference.


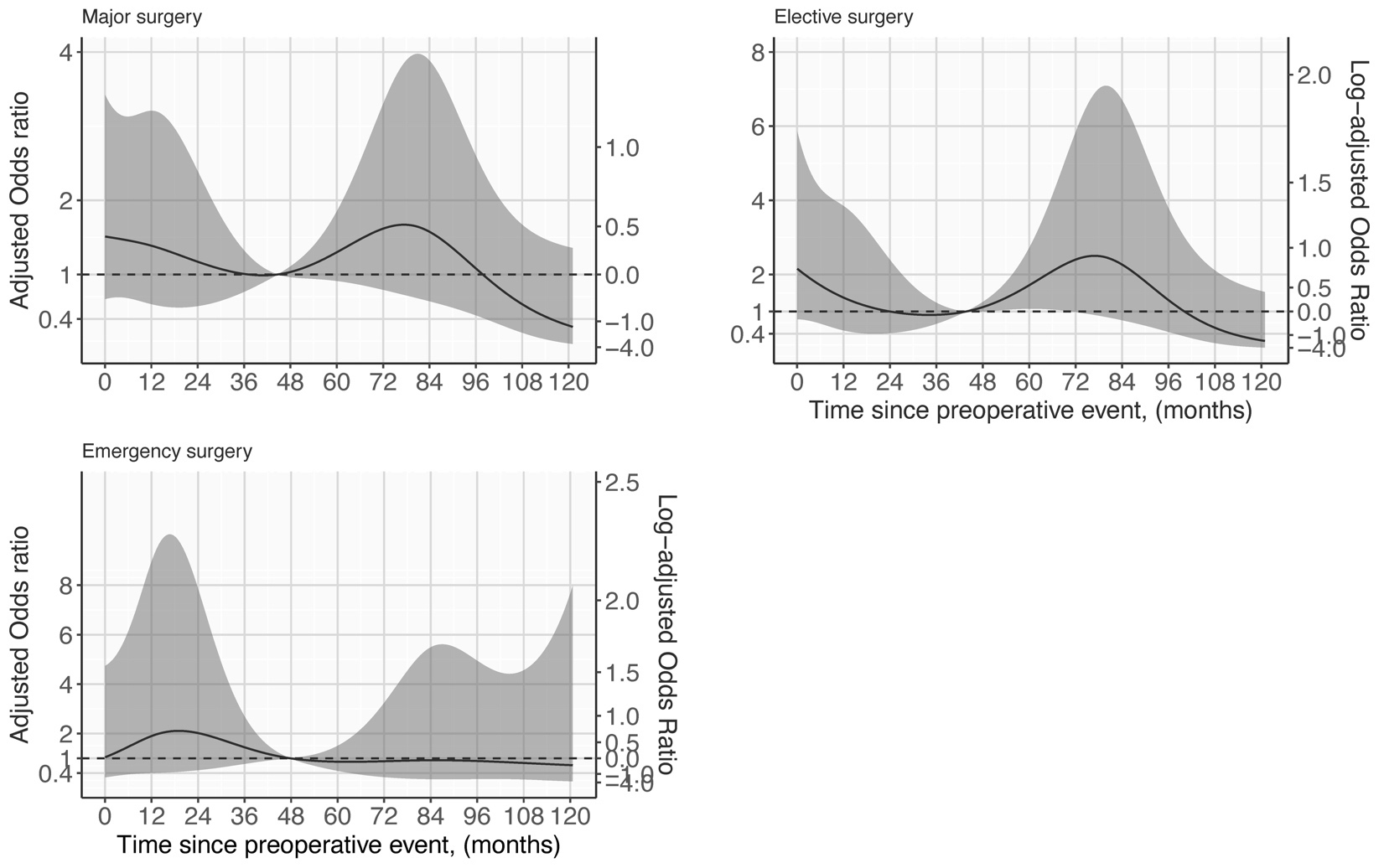


**Figure 30**. Restricted cubic splines logistic regression for 1-year risk of cerebrovascular accident after surgery by the time between the most recent cardiovascular event stratified by surgery invasiveness and urgency. The spline was adjusted for age, sex, index of multiple deprivation, hypertension, atrial fibrillation, stable angina, peripheral vascular disease, valvular heart disease, congestive heart failure, respiratory diseases, diabetes mellitus, renal failure, cancer, liver disease, and dementia. The median time between the event and surgery served as the reference.

##### Lower limb bypass


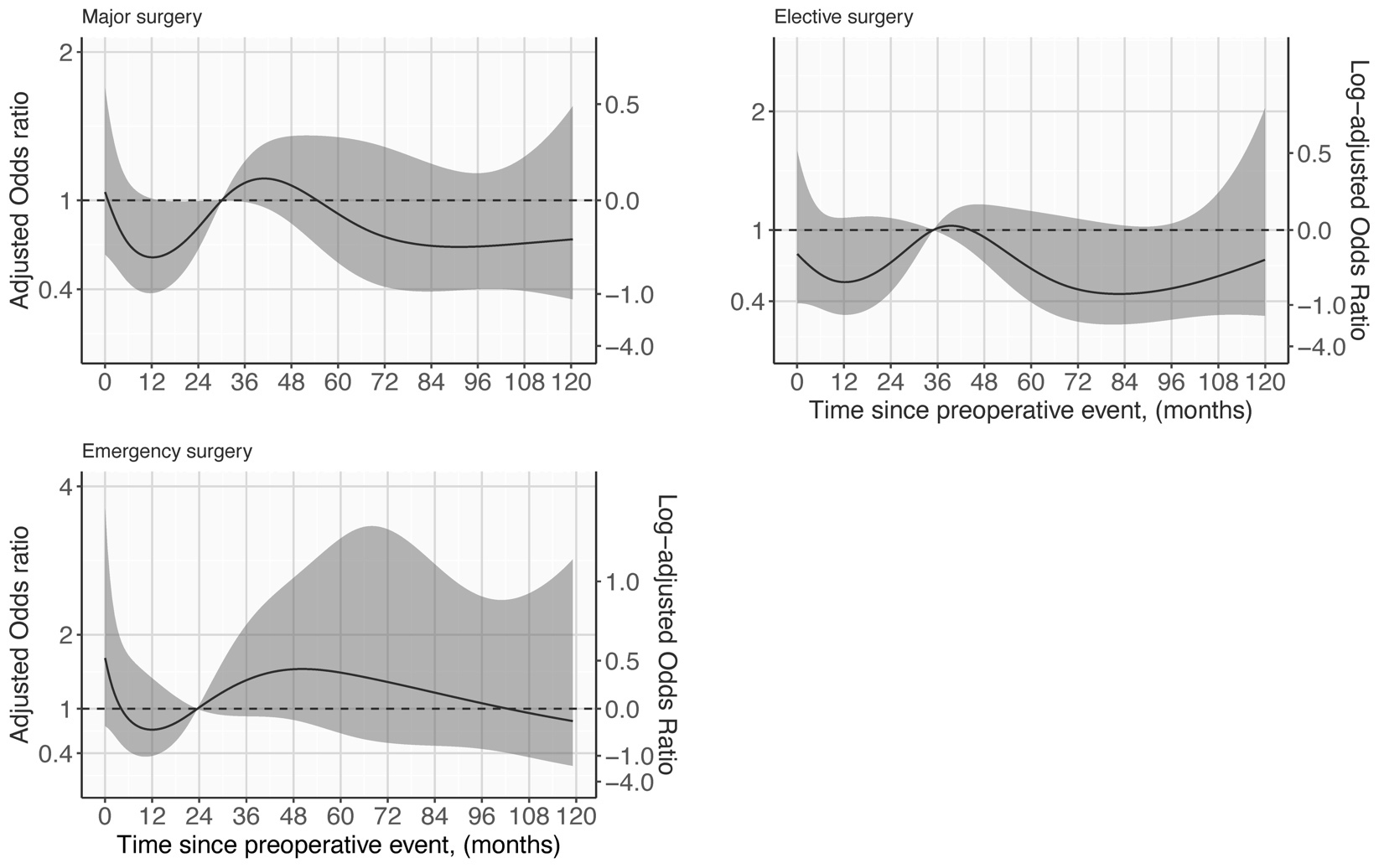


**Figure 31.** Restricted cubic splines logistic regression for 1-year risk of Acute Coronary Syndrome after surgery by the time between the most recent cardiovascular event stratified by surgery invasiveness and urgency. The spline was adjusted for age, sex, index of multiple deprivation, hypertension, atrial fibrillation, stable angina, peripheral vascular disease, valvular heart disease, congestive heart failure, respiratory diseases, diabetes mellitus, renal failure, cancer, liver disease, and dementia. The median time between the event and surgery served as the reference.


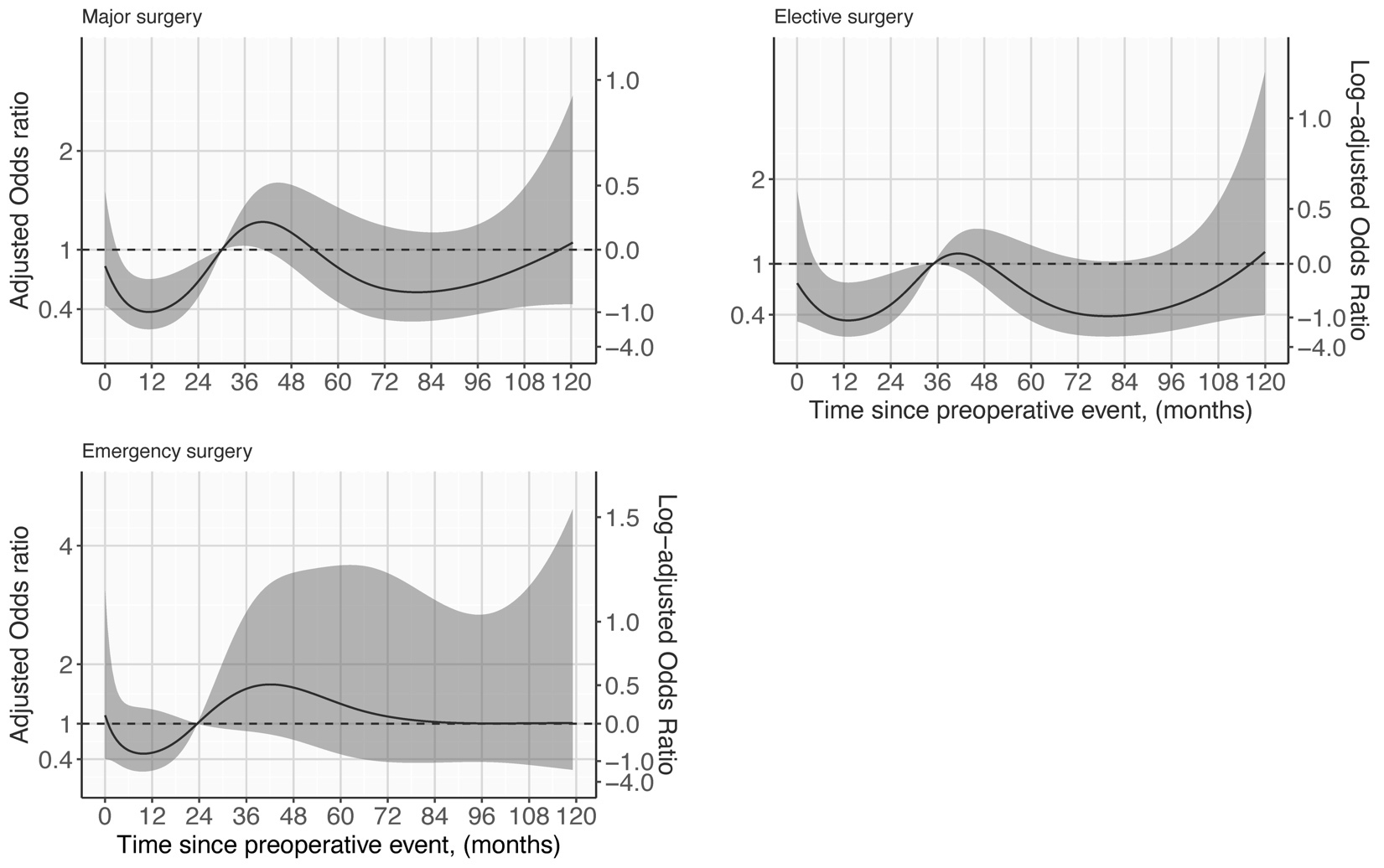


**Figure 32**. Restricted cubic splines logistic regression for 1-year risk of Acute Myocardial infarction after surgery by the time between the most recent cardiovascular event stratified by surgery invasiveness and urgency. The spline was adjusted for age, sex, index of multiple deprivation, hypertension, atrial fibrillation, stable angina, peripheral vascular disease, valvular heart disease, congestive heart failure, respiratory diseases, diabetes mellitus, renal failure, cancer, liver disease, and dementia. The median time between the event and surgery served as the reference.


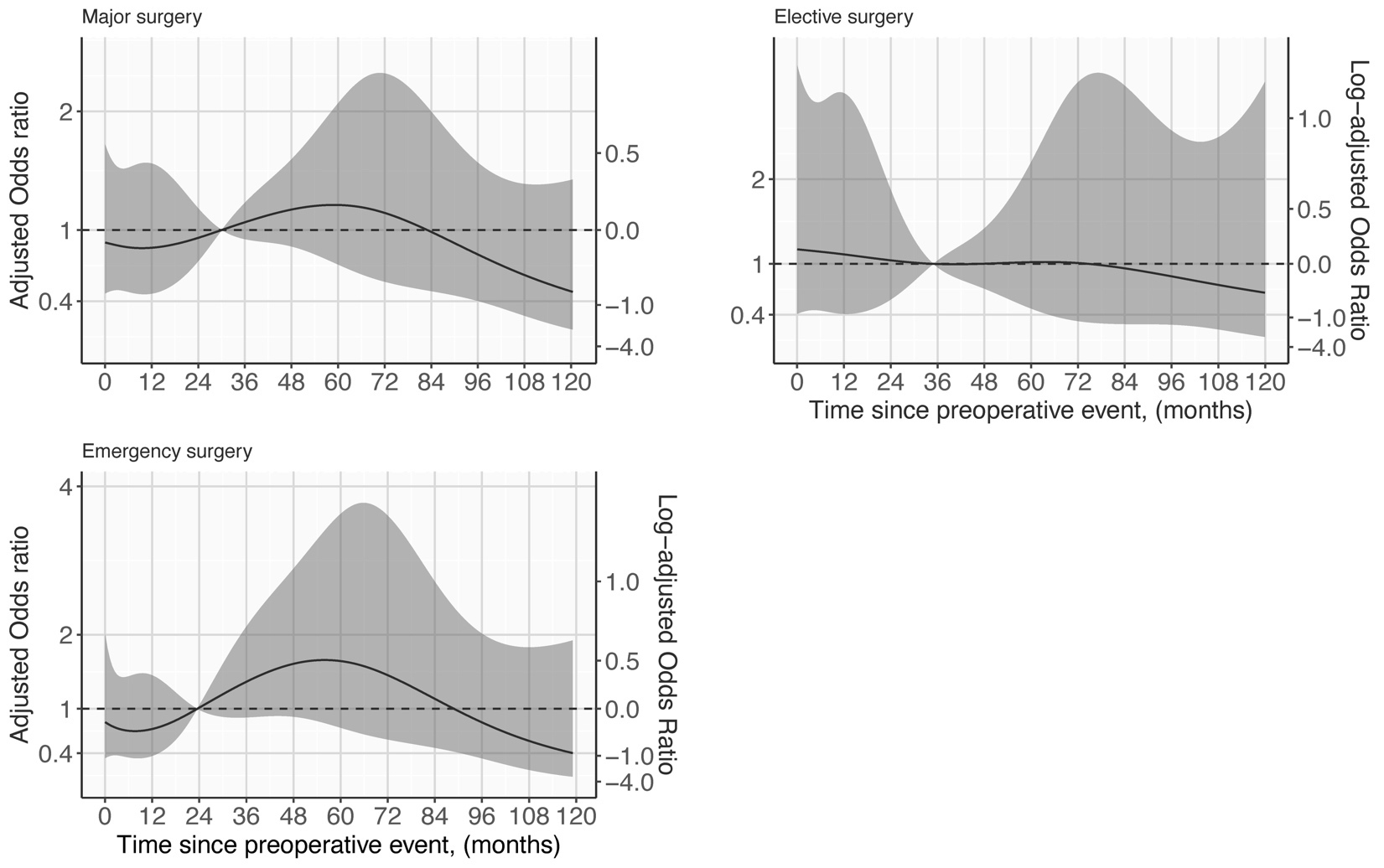


**Figure 33**. Restricted cubic splines logistic regression for 1-year risk of cerebrovascular accident after surgery by the time between the most recent cardiovascular event stratified by surgery invasiveness and urgency. The spline was adjusted for age, sex, index of multiple deprivation, hypertension, atrial fibrillation, stable angina, peripheral vascular disease, valvular heart disease, congestive heart failure, respiratory diseases, diabetes mellitus, renal failure, cancer, liver disease, and dementia. The median time between the event and surgery served as the reference.

### Gastrointestinal surgery


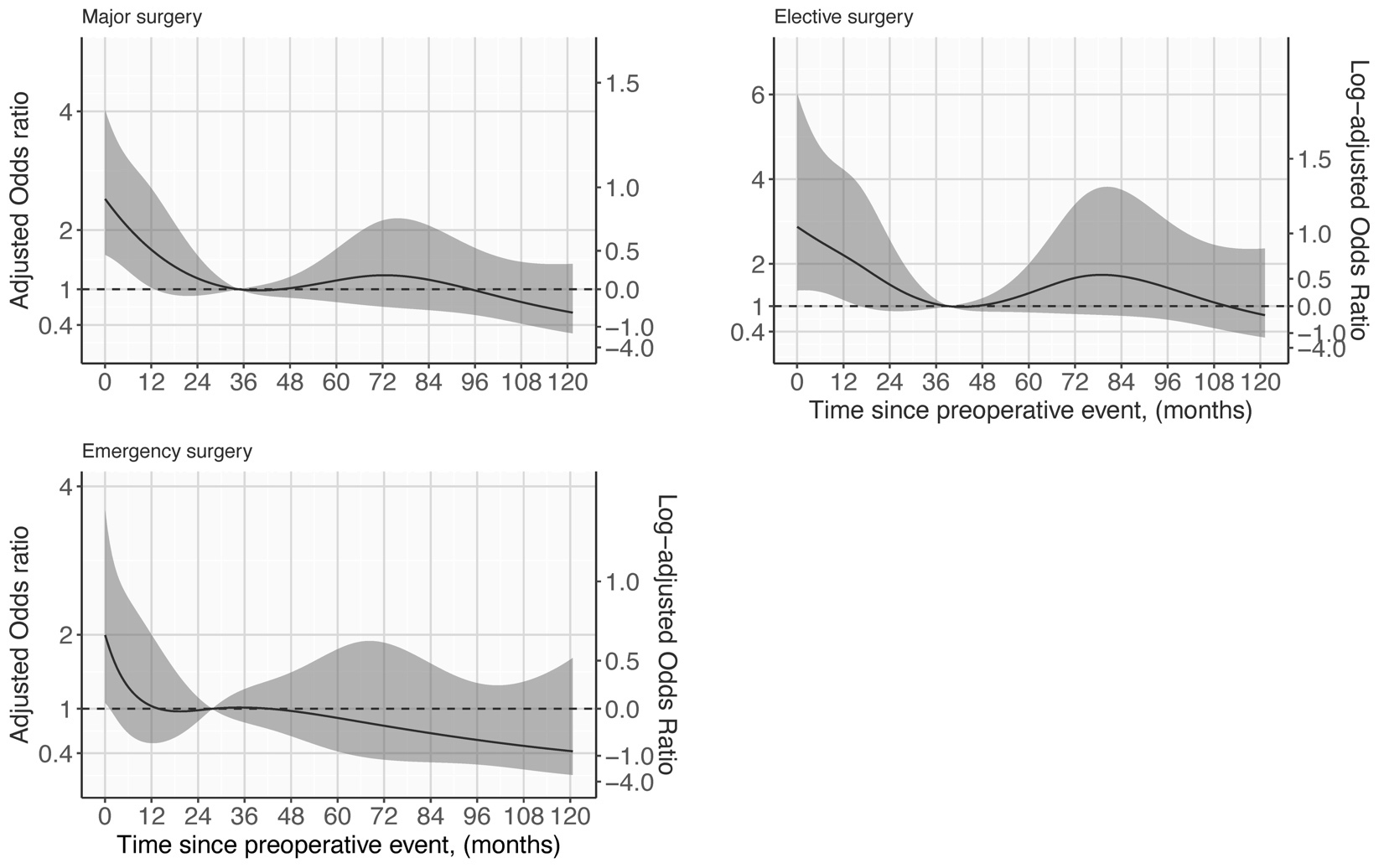


**Figure 34**. Restricted cubic splines logistic regression for 1-year risk of Acute Coronary Syndrome after surgery by the time between the most recent cardiovascular event stratified by surgery invasiveness and urgency. The spline was adjusted for age, sex, index of multiple deprivation, hypertension, atrial fibrillation, stable angina, peripheral vascular disease, valvular heart disease, congestive heart failure, respiratory diseases, diabetes mellitus, renal failure, cancer, liver disease, and dementia. The median time between the event and surgery served as the reference.


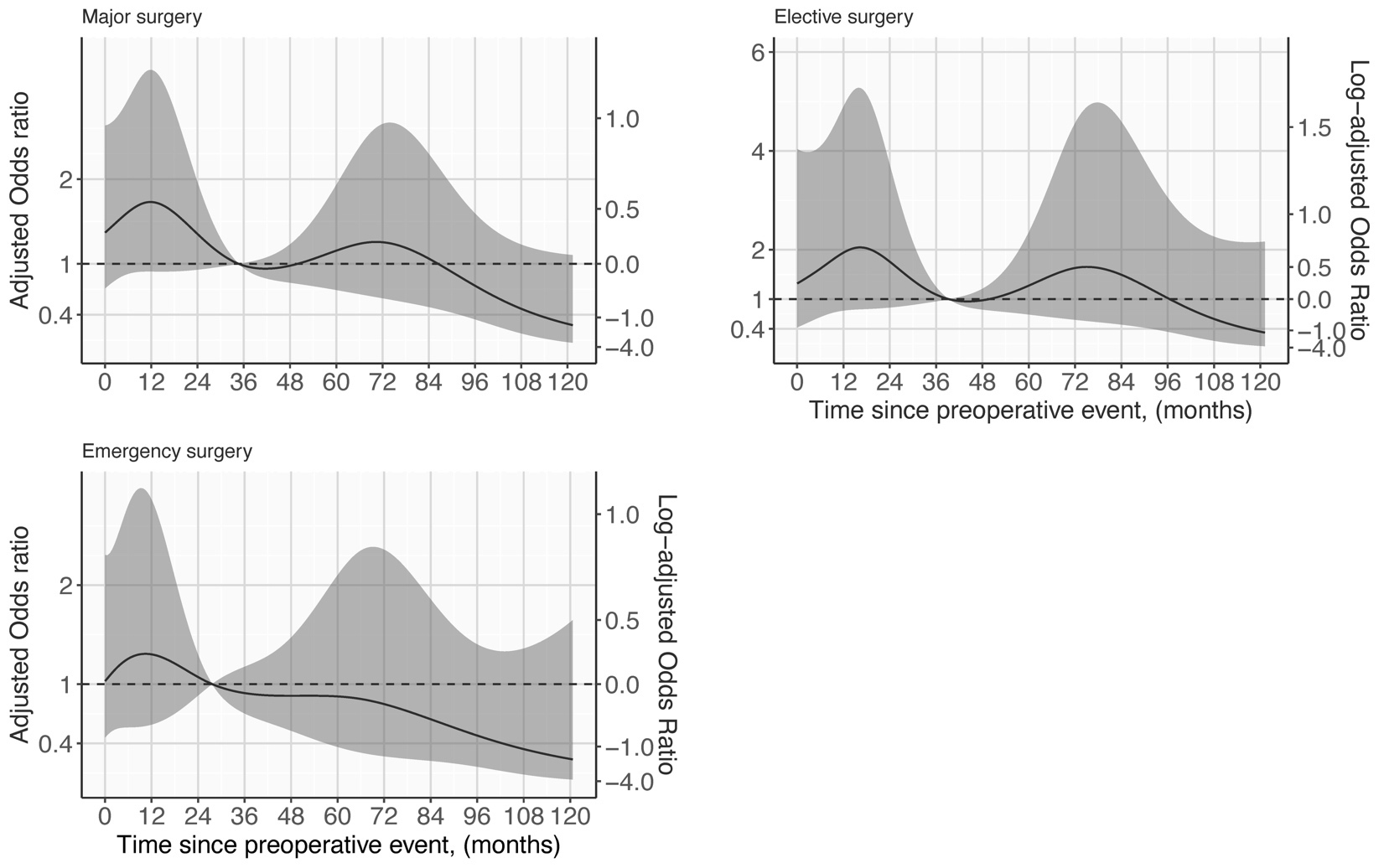


**Figure 35.** Restricted cubic splines logistic regression for 1-year risk of Acute Myocardial infarction after surgery by the time between the most recent cardiovascular event stratified by surgery invasiveness and urgency. The spline was adjusted for age, sex, index of multiple deprivation, hypertension, atrial fibrillation, stable angina, peripheral vascular disease, valvular heart disease, congestive heart failure, respiratory diseases, diabetes mellitus, renal failure, cancer, liver disease, and dementia. The median time between the event and surgery served as the reference.


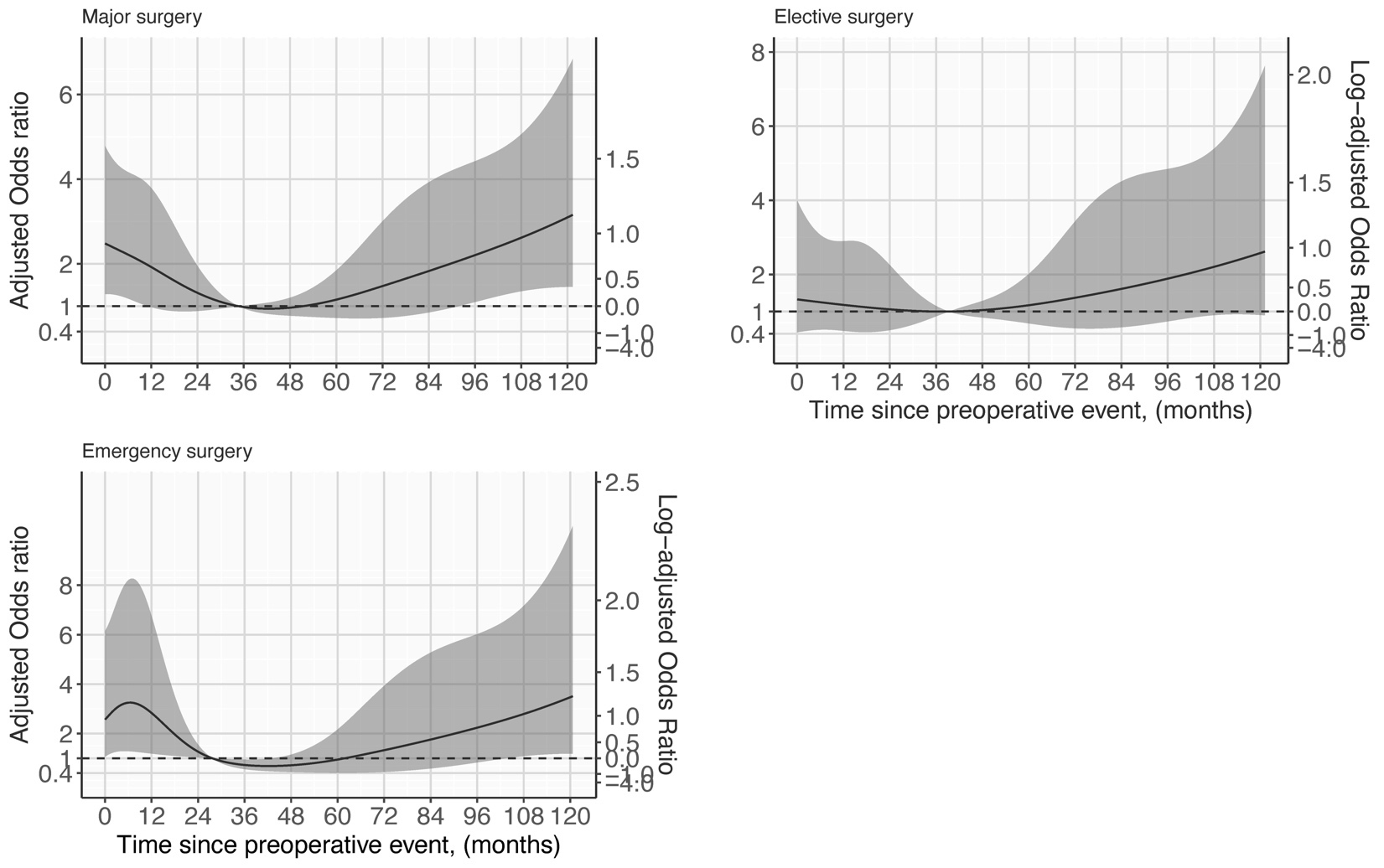


**Figure 36.** Restricted cubic splines logistic regression for 1-year risk of cerebrovascular accident after surgery by the time between the most recent cardiovascular event stratified by surgery invasiveness and urgency. The spline was adjusted for age, sex, index of multiple deprivation, hypertension, atrial fibrillation, stable angina, peripheral vascular disease, valvular heart disease, congestive heart failure, respiratory diseases, diabetes mellitus, renal failure, cancer, liver disease, and dementia. The median time between the event and surgery served as the reference.

#### Adverse outcomes by subtype of gastrointestinal surgery

##### Colorectal major resection


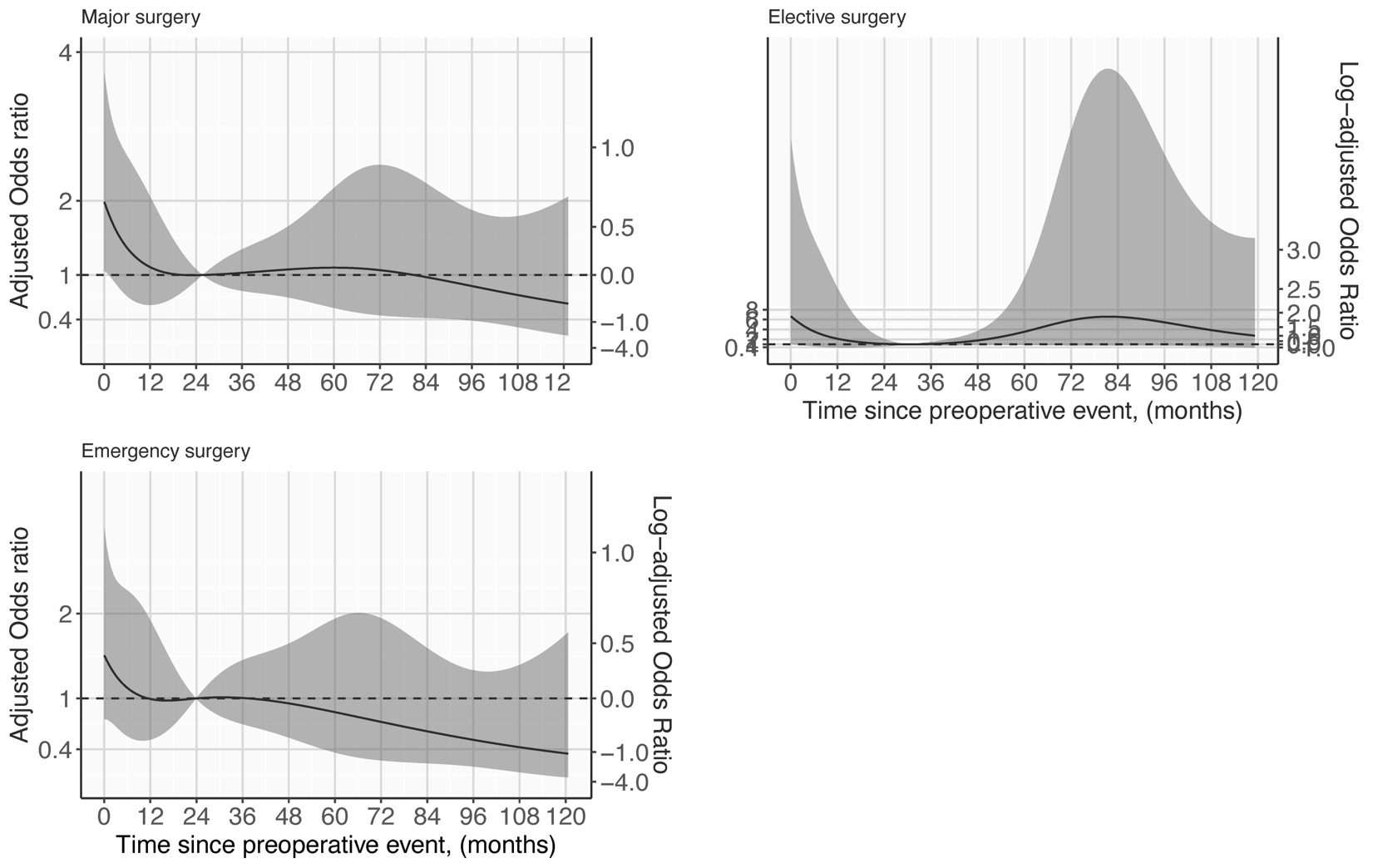


**Figure 37.** Restricted cubic splines logistic regression for 1-year risk of Acute Coronary Syndrome after surgery by the time between the most recent cardiovascular event stratified by surgery invasiveness and urgency. The spline was adjusted for age, sex, index of multiple deprivation, hypertension, atrial fibrillation, stable angina, peripheral vascular disease, valvular heart disease, congestive heart failure, respiratory diseases, diabetes mellitus, renal failure, cancer, liver disease, and dementia. The median time between the event and surgery served as the reference.


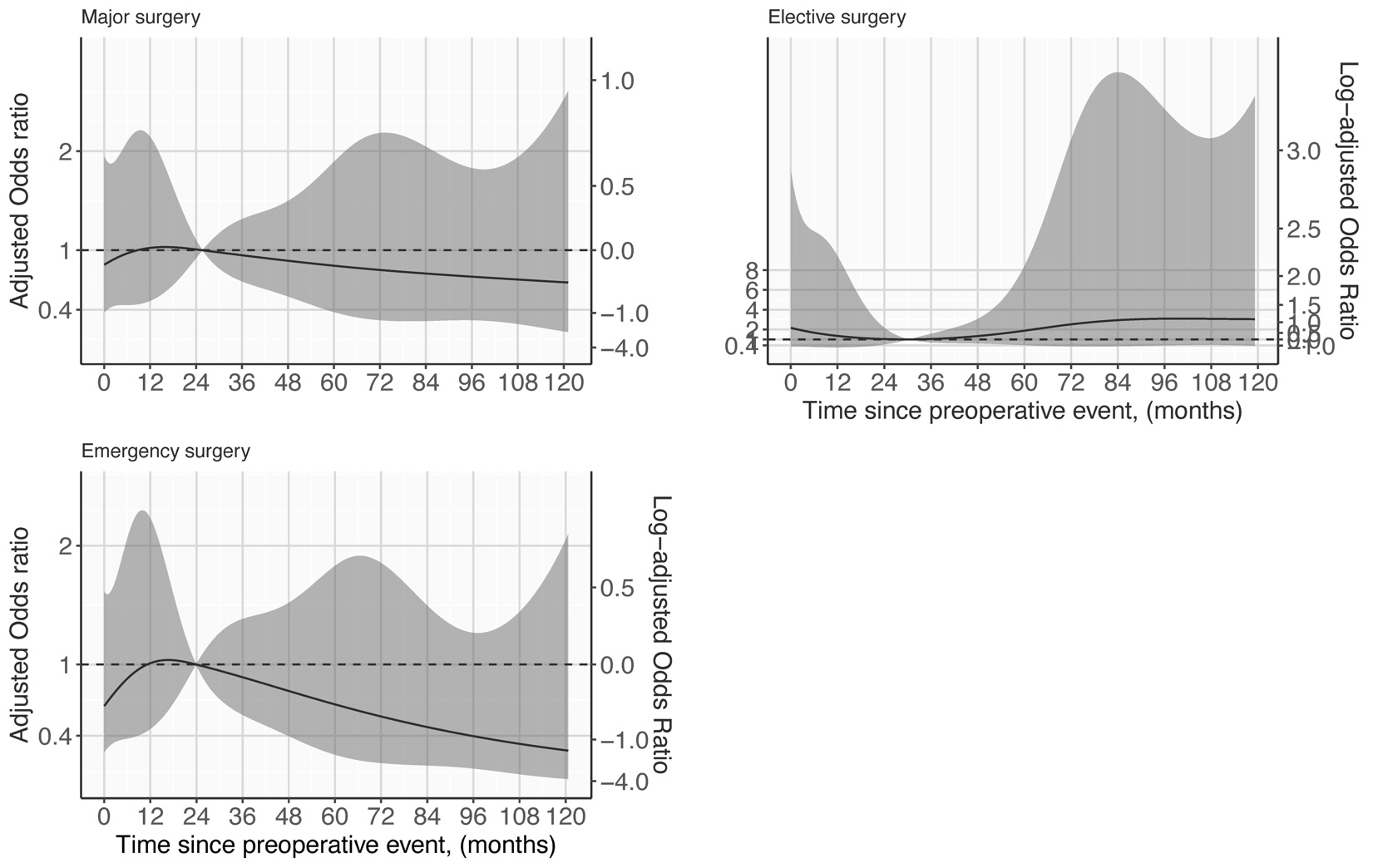


**Figure 38**. Restricted cubic splines logistic regression for 1-year risk of Acute Myocardial infarction after surgery by the time between the most recent cardiovascular event stratified by surgery invasiveness and urgency. The spline was adjusted for age, sex, index of multiple deprivation, hypertension, atrial fibrillation, stable angina, peripheral vascular disease, valvular heart disease, congestive heart failure, respiratory diseases, diabetes mellitus, renal failure, cancer, liver disease, and dementia. The median time between the event and surgery served as the reference.


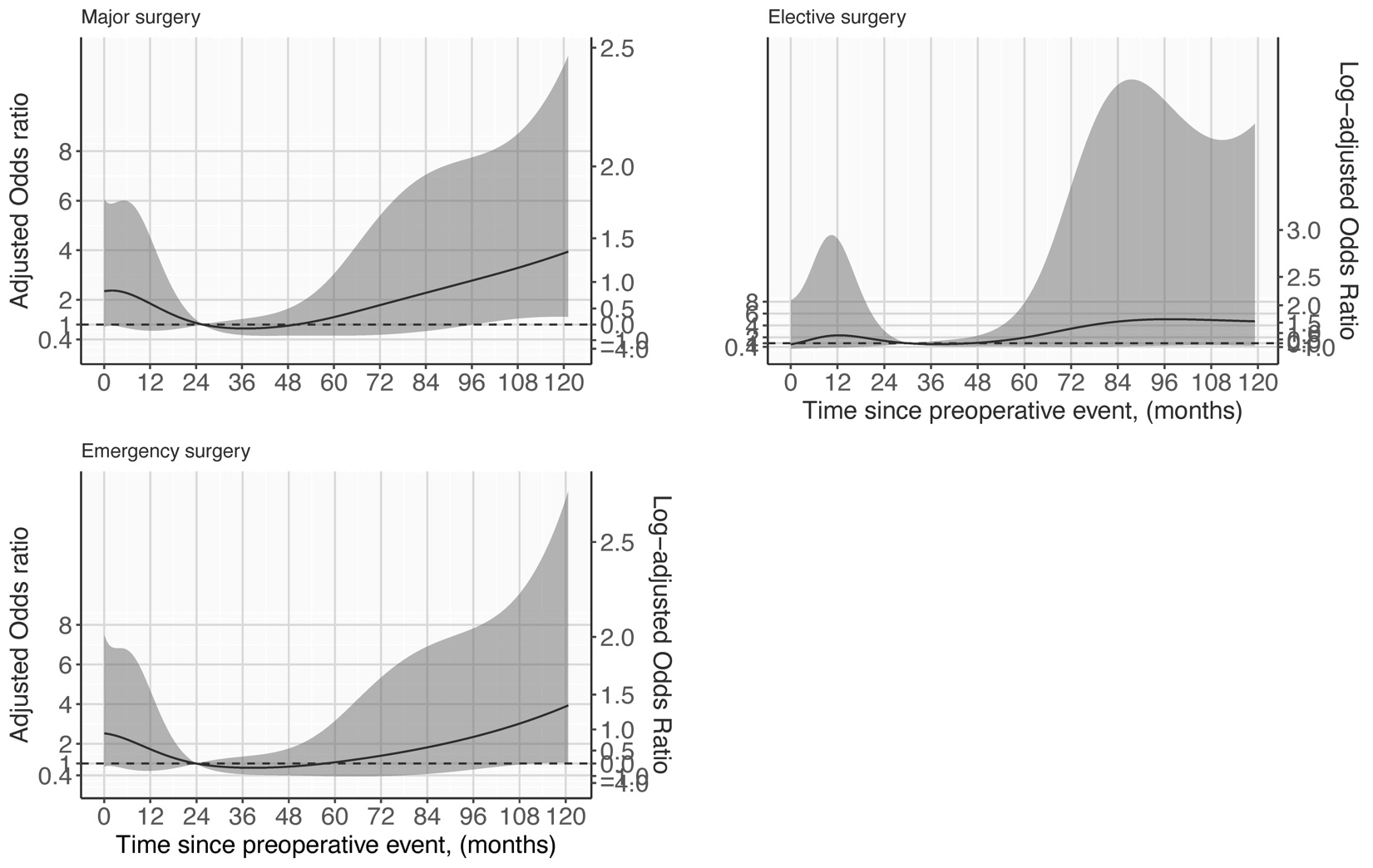


**Figure 39**. Restricted cubic splines logistic regression for 1-year risk of cerebrovascular accident after surgery by the time between the most recent cardiovascular event stratified by surgery invasiveness and urgency. The spline was adjusted for age, sex, index of multiple deprivation, hypertension, atrial fibrillation, stable angina, peripheral vascular disease, valvular heart disease, congestive heart failure, respiratory diseases, diabetes mellitus, renal failure, cancer, liver disease, and dementia. The median time between the event and surgery served as the reference.

##### Cholecystectomy


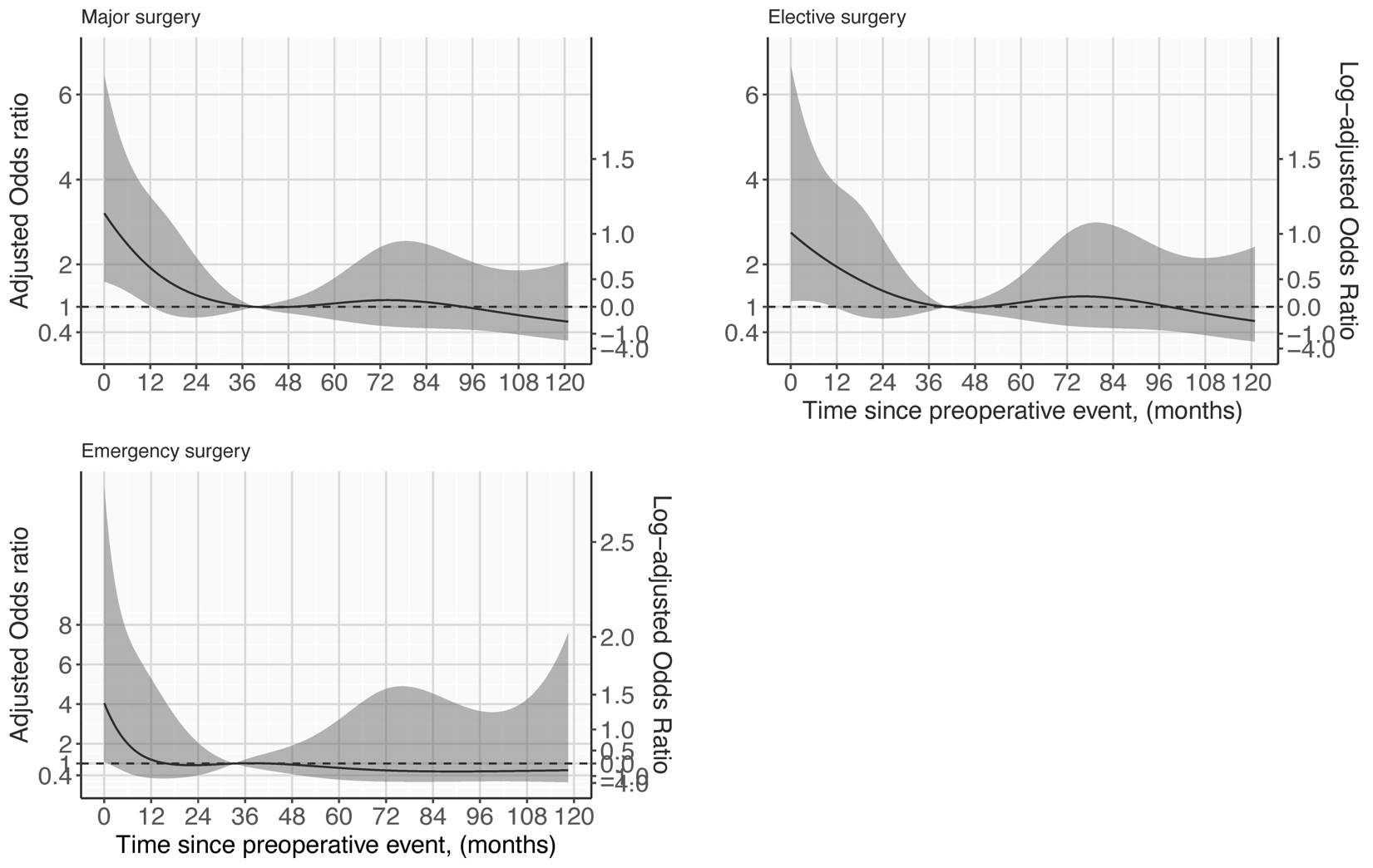


**Figure 40**. Restricted cubic splines logistic regression for 1-year risk of Acute Coronary Syndrome after surgery by the time between the most recent cardiovascular event stratified by surgery invasiveness and urgency. The spline was adjusted for age, sex, index of multiple deprivation, hypertension, atrial fibrillation, stable angina, peripheral vascular disease, valvular heart disease, congestive heart failure, respiratory diseases, diabetes mellitus, renal failure, cancer, liver disease, and dementia. The median time between the event and surgery served as the reference.


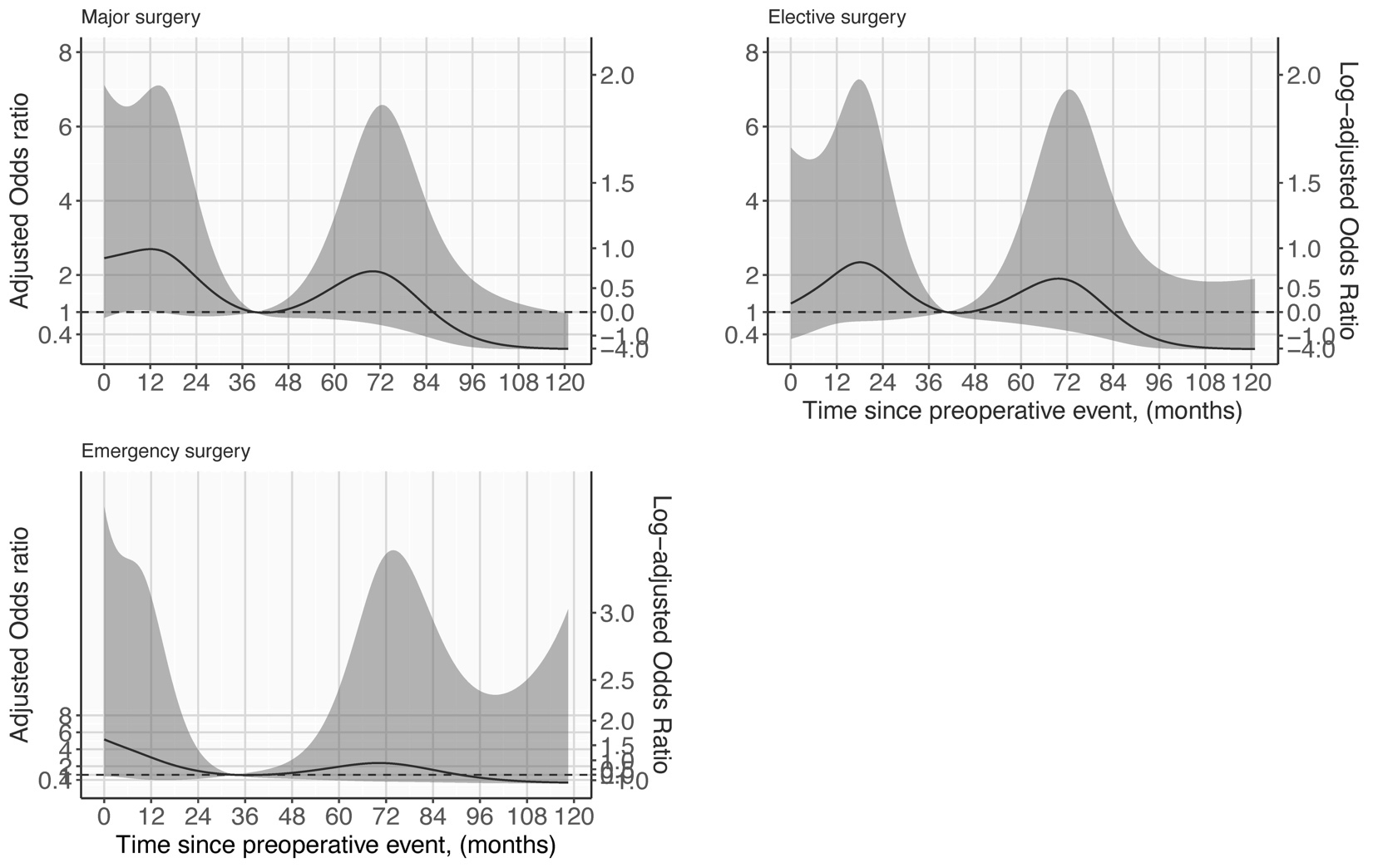


**Figure 41**. Restricted cubic splines logistic regression for 1-year risk of Acute Myocardial infarction after surgery by the time between the most recent cardiovascular event stratified by surgery invasiveness and urgency. The spline was adjusted for age, sex, index of multiple deprivation, hypertension, atrial fibrillation, stable angina, peripheral vascular disease, valvular heart disease, congestive heart failure, respiratory diseases, diabetes mellitus, renal failure, cancer, liver disease, and dementia. The median time between the event and surgery served as the reference.


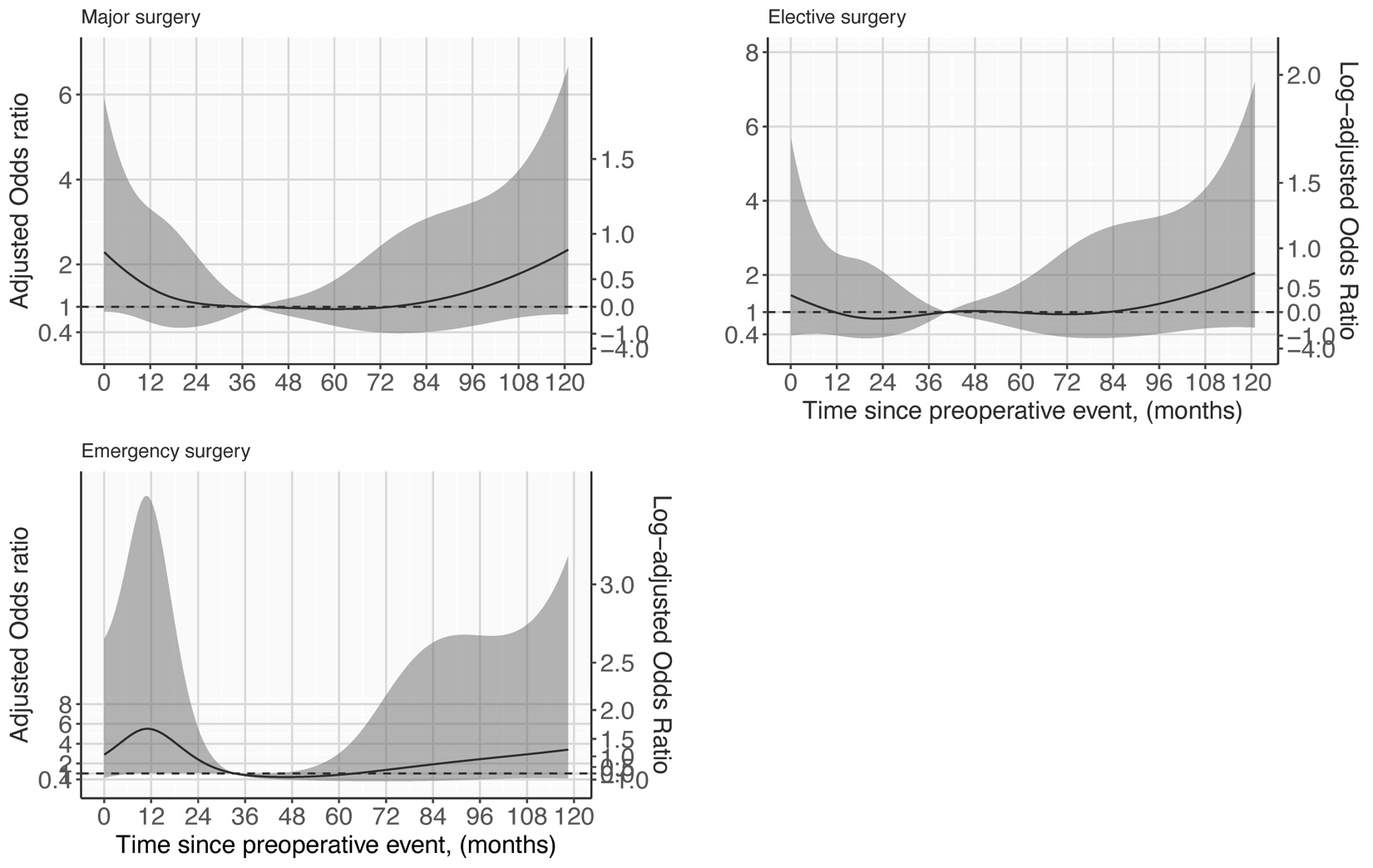


**Figure 42**. Restricted cubic splines logistic regression for 1-year risk of cerebrovascular accident after surgery by the time between the most recent cardiovascular event stratified by surgery invasiveness and urgency. The spline was adjusted for age, sex, index of multiple deprivation, hypertension, atrial fibrillation, stable angina, peripheral vascular disease, valvular heart disease, congestive heart failure, respiratory diseases, diabetes mellitus, renal failure, cancer, liver disease, and dementia. The median time between the event and surgery served as the reference.

## Adverse outcomes by subtype of cardiovascular event

**Table 2**. Crude number of adverse events by subtype of cardiovascular event, surgical invasiveness, and surgical urgency.

|  | **Surgical invasiveness** | | | **Surgical Urgency** | |
| --- | --- | --- | --- | --- | --- |
| **Subtype of cardiovascular event** | **Minor** | **Moderate** | **Major** | **Elective** | **Emergency** |
| **Acute coronary syndrome within 1-year after surgery** | | | | | |
| **Haemorrhagic stroke** | 100 (1.3) | 131 (1.1) | 37 (0.7) | 167 (1.1) | 101 (1) |
| **Ischaemic stroke** | 1275 (2) | 1162 (1.8) | 563 (1.7) | 1774 (1.8) | 1226 (2.1) |
| **Unspecified stroke** | 523 (2.5) | 421 (2) | 191 (1.8) | 694 (2) | 441 (2.5) |
| **Myocardial infarction** | 4651 (3.6) | 4078 (3) | 4659 (2.7) | 6906 (2.9) | 3482 (3.8) |
| **Transient ischaemic attack** | 816 (2.3) | 763 (2) | 334 (1.8) | 1324 (2) | 589 (2.4) |
| **Unstable angina** | 3149 (3.4) | 2627 (3.1) | 1251 (3.1) | 4410 (2.6) | 2617 (5.3) |
| **Acute myocardial infraction within 1-year after surgery** | | | | | |
| **Haemorrhagic stroke** | 80 (1) | 94 (0.8) | 31 (0.6) | 126 (0.8) | 77 (0.8) |
| **Ischaemic stroke** | 876 (1.4) | 871 (1.3) | 428 (1.3) | 1282 (1.3) | 893 (1.5) |
| **Unspecified stroke** | 331 (1.6) | 289 (1.4) | 134 (1.3) | 456 (1.3) | 298 (1.7) |
| **Myocardial infarction** | 1709 (1.3) | 1644 (1.2) | 632 (1) | 2619 (1.1) | 1366 (1.5) |
| **Transient ischaemic attack** | 532 (1.5) | 516 (1.4) | 227 (1.2) | 870 (1.3) | 405 (1.6) |
| **Unstable angina** | 2014 (2.2) | 1807 (2.1) | 902 (2.2) | 2696 (1.6) | 2027 (4.1) |
| **Cerebrovascular accident within 1-year after surgery** | | | | | |
| **Haemorrhagic stroke** | 306 (3.9) | 435 (3.7) | 244 (4.5) | 491 (3.2) | 494 (5.1) |
| **Ischaemic stroke** | 1570 (2.5) | 1546 (2.4) | 844 (2.6) | 2052 (2) | 1908 (3.2) |
| **Unspecified stroke** | 763 (3.6) | 672 (3.2) | 378 (3.6) | 1057 (3) | 756 (4.3) |
| **Myocardial infarction** | 1593 (1.2) | 1652 (1.2) | 771 (1.2) | 2451 (1) | 1565 (1.7) |
| **Transient ischaemic attack** | 1034 (2.9) | 1064 (2.8) | 497 (2.7) | 1630 (2.4) | 965 (3.9) |
| **Unstable angina** | 1139 (1.2) | 1074 (1.3) | 497 (1.2) | 1732 (1) | 978 (2) |

##### Haemorrhagic stroke


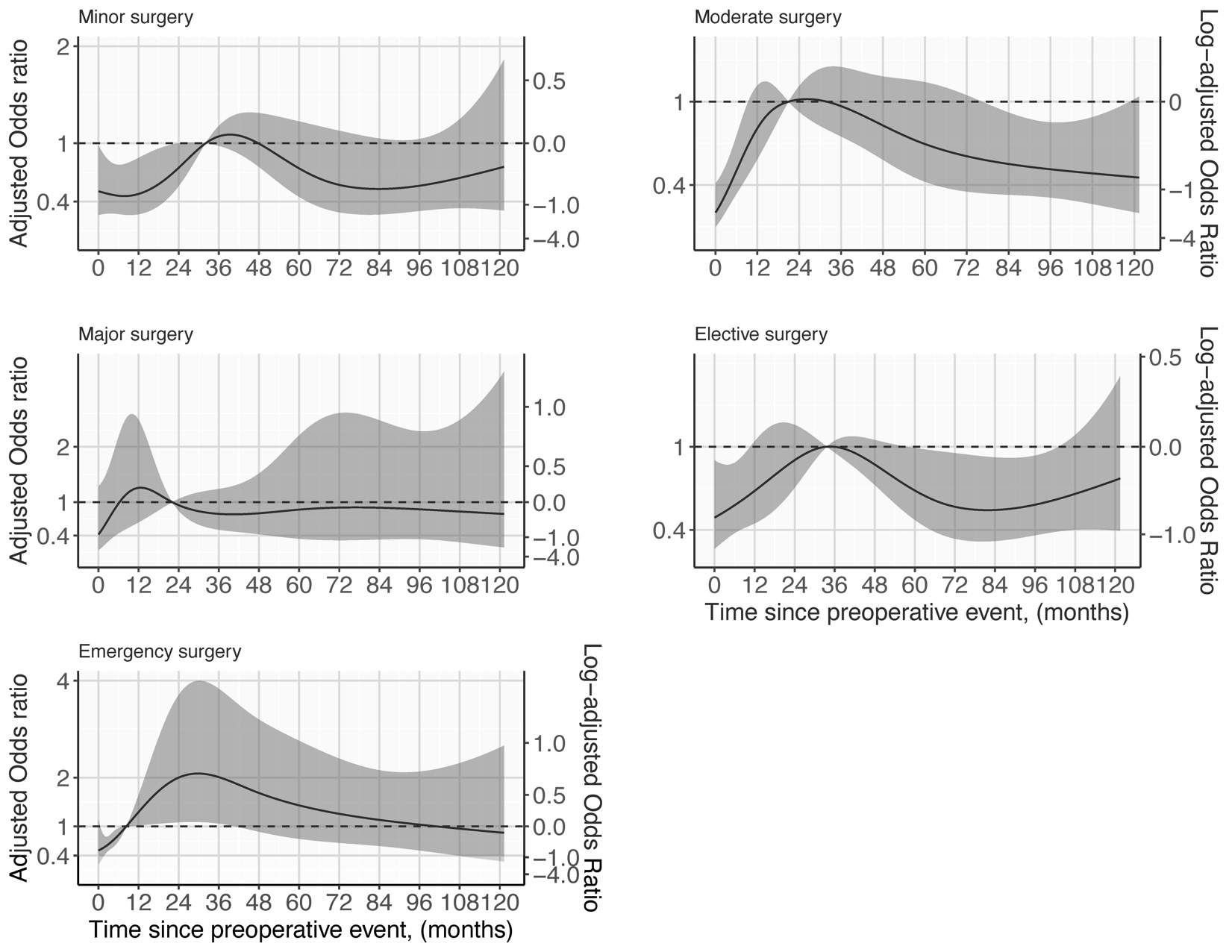


**Figure 43**. Restricted cubic splines logistic regression for 1-year risk of Acute Coronary Syndrome after surgery by the time between the most recent cardiovascular event stratified by surgery invasiveness and urgency. The spline was adjusted for age, sex, index of multiple deprivation, hypertension, atrial fibrillation, stable angina, peripheral vascular disease, valvular heart disease, congestive heart failure, respiratory diseases, diabetes mellitus, renal failure, cancer, liver disease, and dementia. The median time between the event and surgery served as the reference.


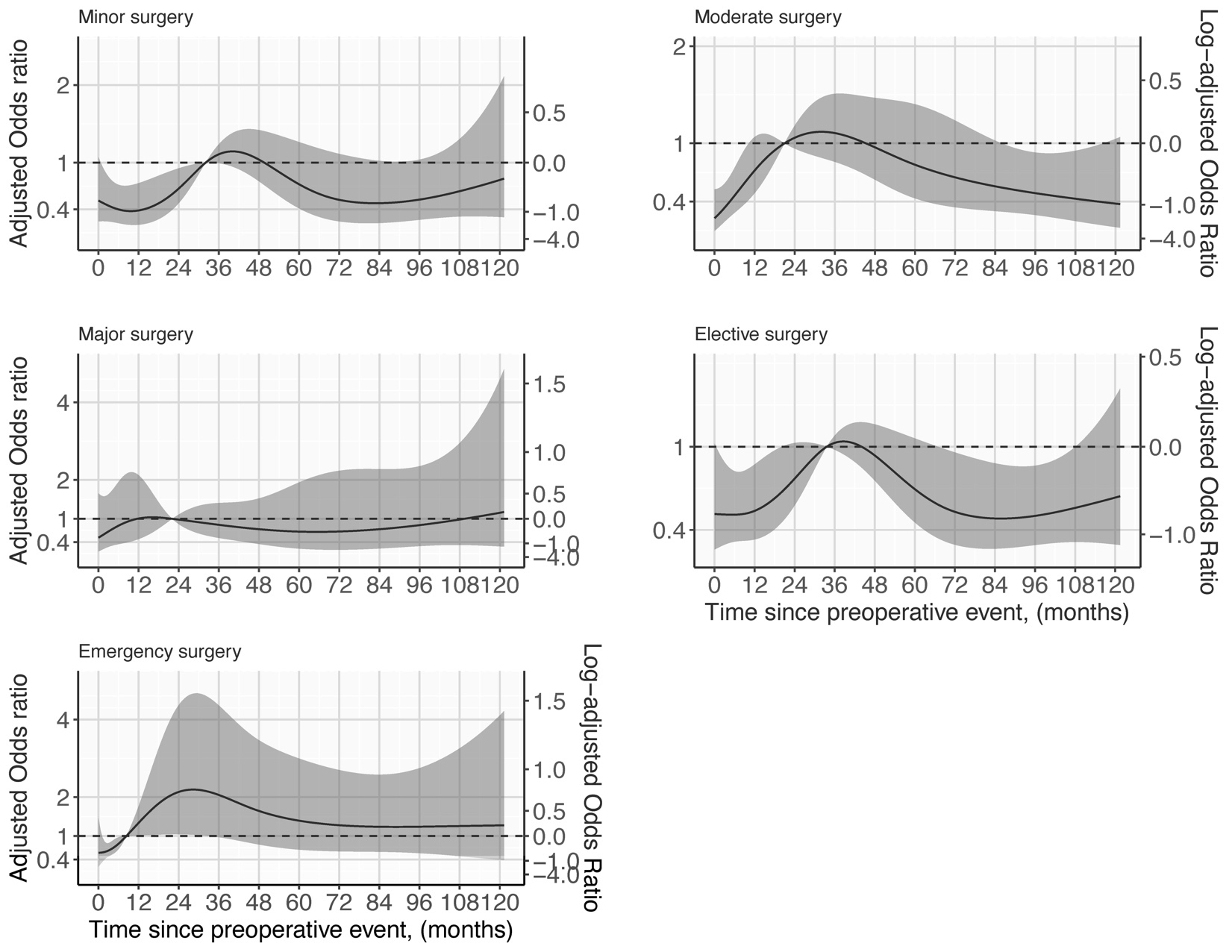


**Figure 44.** Restricted cubic splines logistic regression for 1-year risk of Acute Myocardial infarction after surgery by the time between the most recent cardiovascular event stratified by surgery invasiveness and urgency. The spline was adjusted for age, sex, index of multiple deprivation, hypertension, atrial fibrillation, stable angina, peripheral vascular disease, valvular heart disease, congestive heart failure, respiratory diseases, diabetes mellitus, renal failure, cancer, liver disease, and dementia. The median time between the event and surgery served as the reference.


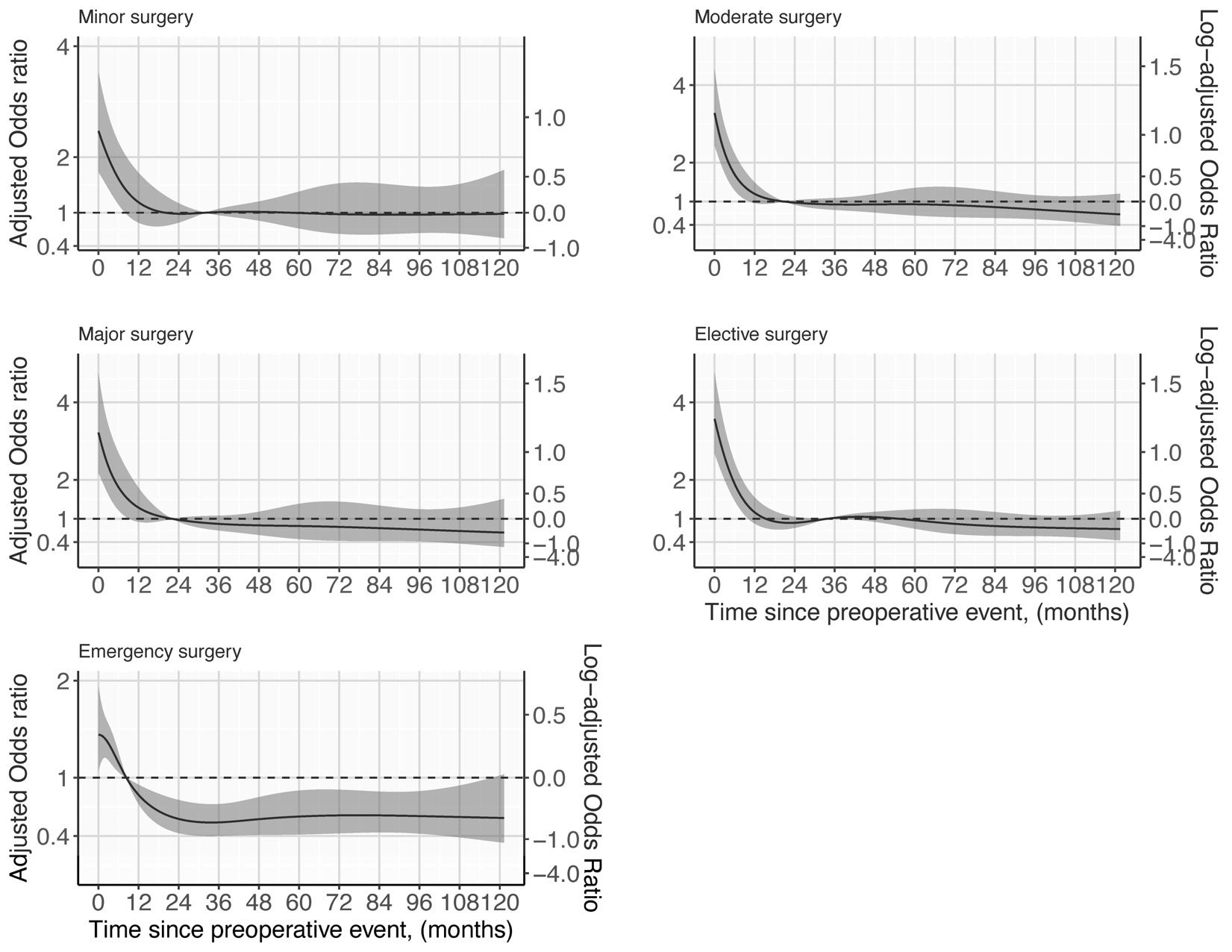


**Figure 45.** Restricted cubic splines logistic regression for 1-year risk of cerebrovascular accident after surgery by the time between the most recent cardiovascular event stratified by surgery invasiveness and urgency. The spline was adjusted for age, sex, index of multiple deprivation, hypertension, atrial fibrillation, stable angina, peripheral vascular disease, valvular heart disease, congestive heart failure, respiratory diseases, diabetes mellitus, renal failure, cancer, liver disease, and dementia. The median time between the event and surgery served as the reference.

##### Ischaemic stroke


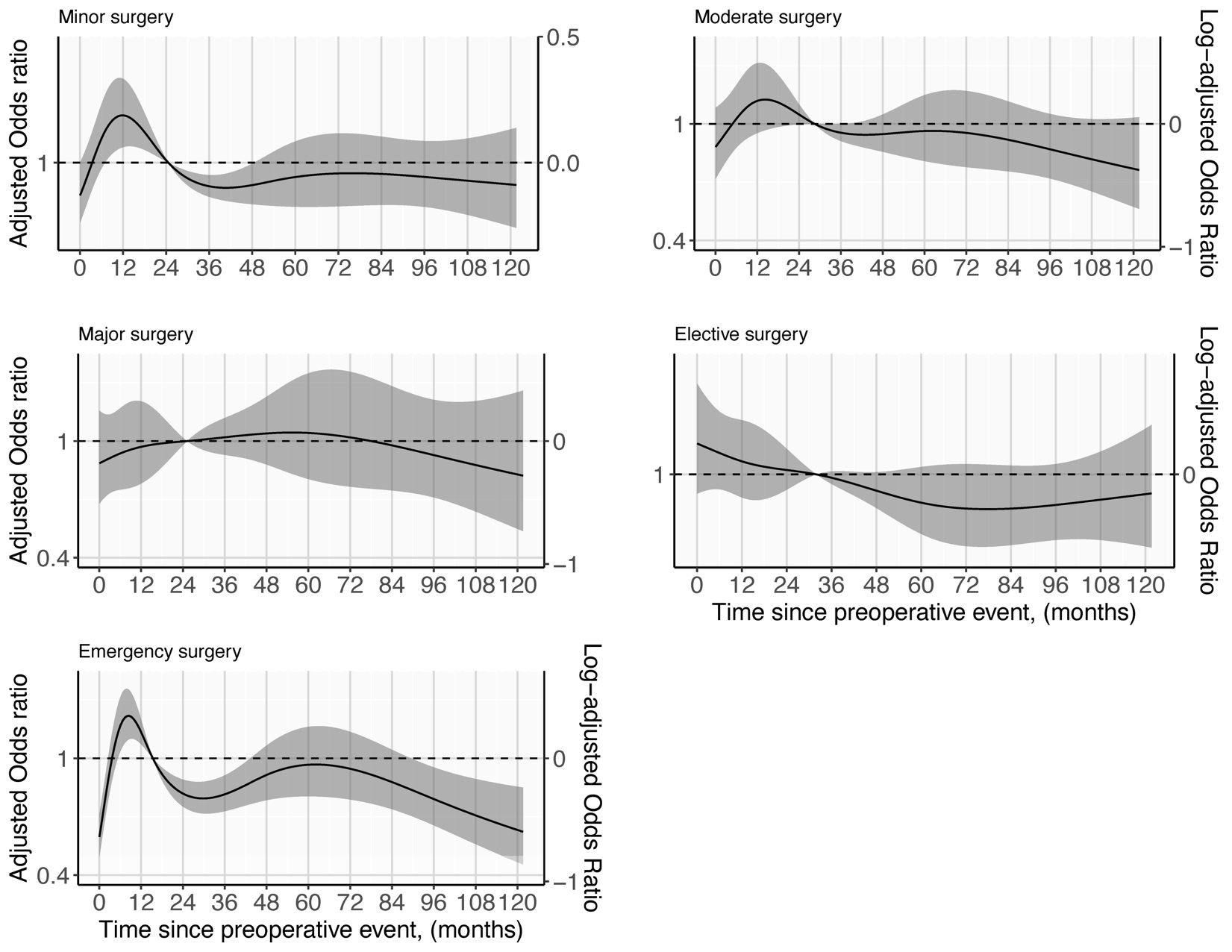


**Figure 46**. Restricted cubic splines logistic regression for 1-year risk of Acute Coronary Syndrome after surgery by the time between the most recent cardiovascular event stratified by surgery invasiveness and urgency. The spline was adjusted for age, sex, index of multiple deprivation, hypertension, atrial fibrillation, stable angina, peripheral vascular disease, valvular heart disease, congestive heart failure, respiratory diseases, diabetes mellitus, renal failure, cancer, liver disease, and dementia. The median time between the event and surgery served as the reference.


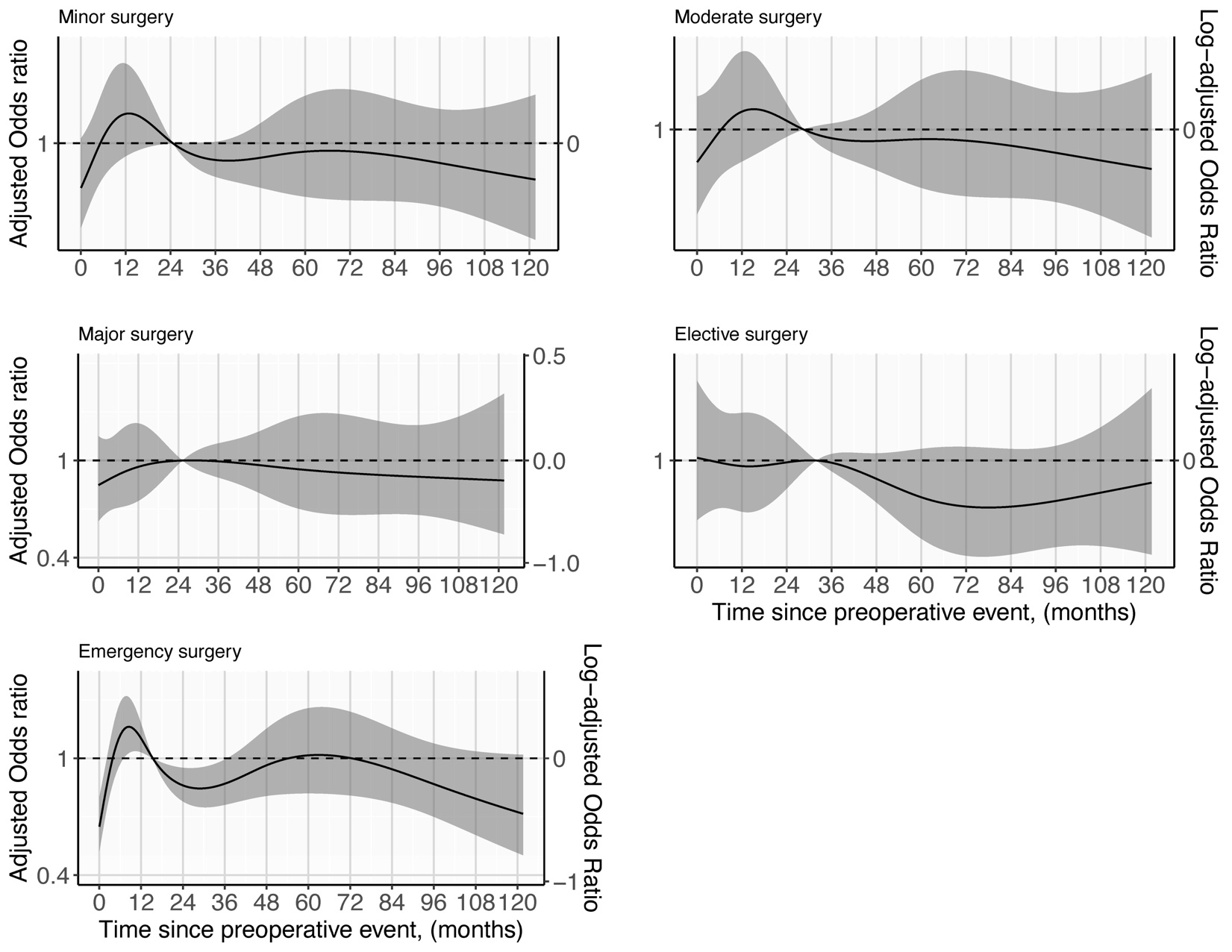


**Figure 47.** Restricted cubic splines logistic regression for 1-year risk of Acute Myocardial infarction after surgery by the time between the most recent cardiovascular event stratified by surgery invasiveness and urgency. The spline was adjusted for age, sex, index of multiple deprivation, hypertension, atrial fibrillation, stable angina, peripheral vascular disease, valvular heart disease, congestive heart failure, respiratory diseases, diabetes mellitus, renal failure, cancer, liver disease, and dementia. The median time between the event and surgery served as the reference.


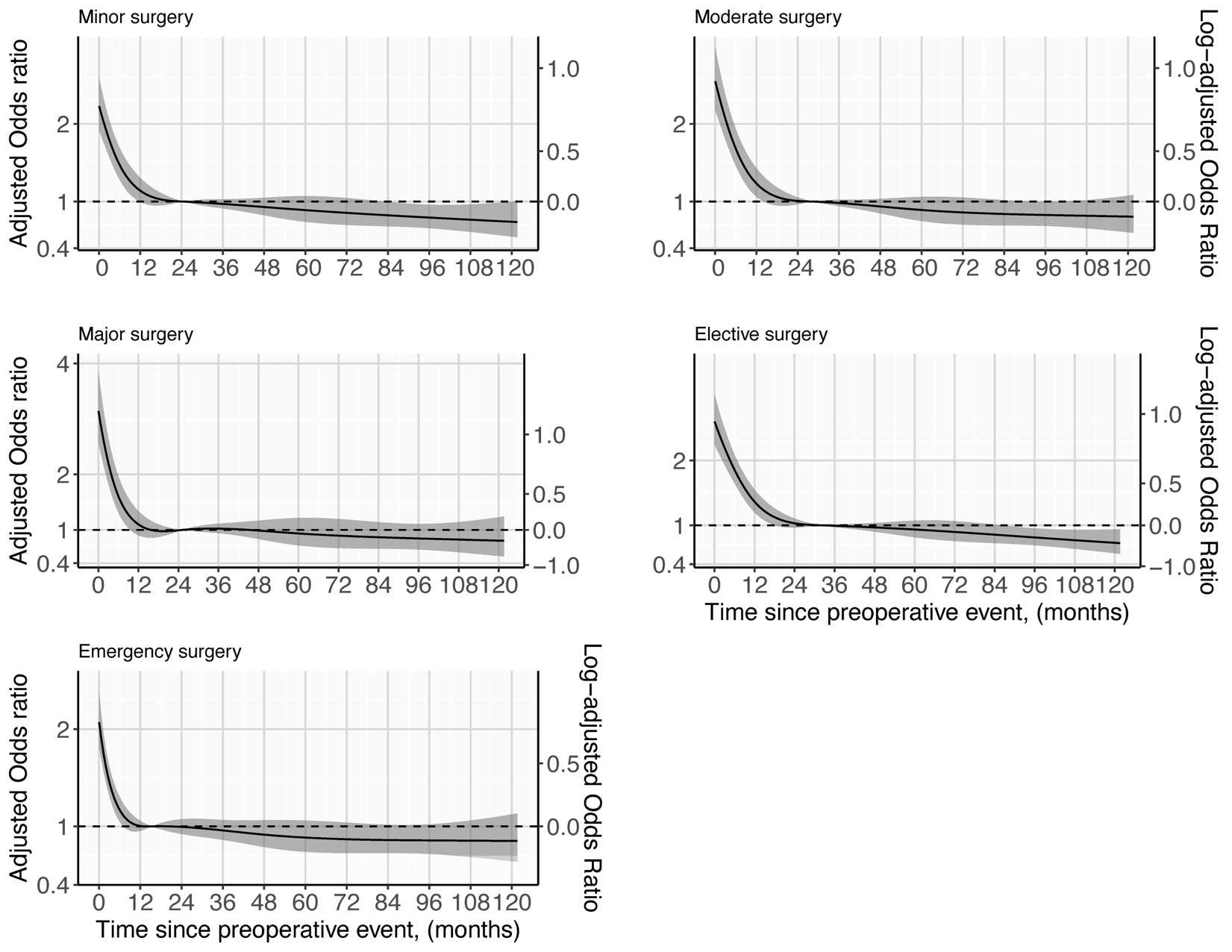


**Figure 48**. Restricted cubic splines logistic regression for 1-year risk of cerebrovascular accident after surgery by the time between the most recent cardiovascular event stratified by surgery invasiveness and urgency. The spline was adjusted for age, sex, index of multiple deprivation, hypertension, atrial fibrillation, stable angina, peripheral vascular disease, valvular heart disease, congestive heart failure, respiratory diseases, diabetes mellitus, renal failure, cancer, liver disease, and dementia. The median time between the event and surgery served as the reference.

##### Unspecified stroke


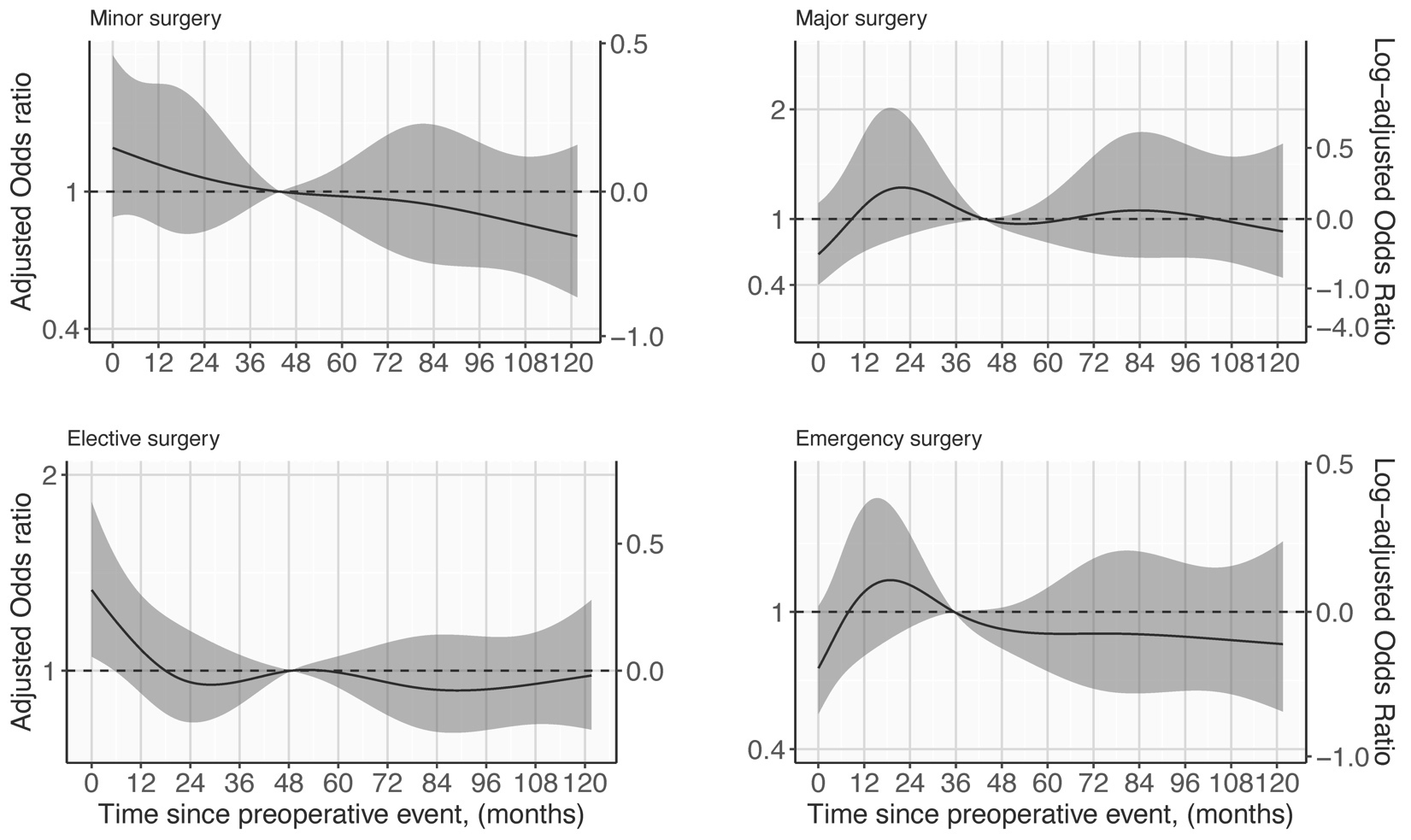


**Figure 49**. Restricted cubic splines logistic regression for 1-year risk of Acute Coronary Syndrome after surgery by the time between the most recent cardiovascular event stratified by surgery invasiveness and urgency. The spline was adjusted for age, sex, index of multiple deprivation, hypertension, atrial fibrillation, stable angina, peripheral vascular disease, valvular heart disease, congestive heart failure, respiratory diseases, diabetes mellitus, renal failure, cancer, liver disease, and dementia. The median time between the event and surgery served as the reference.


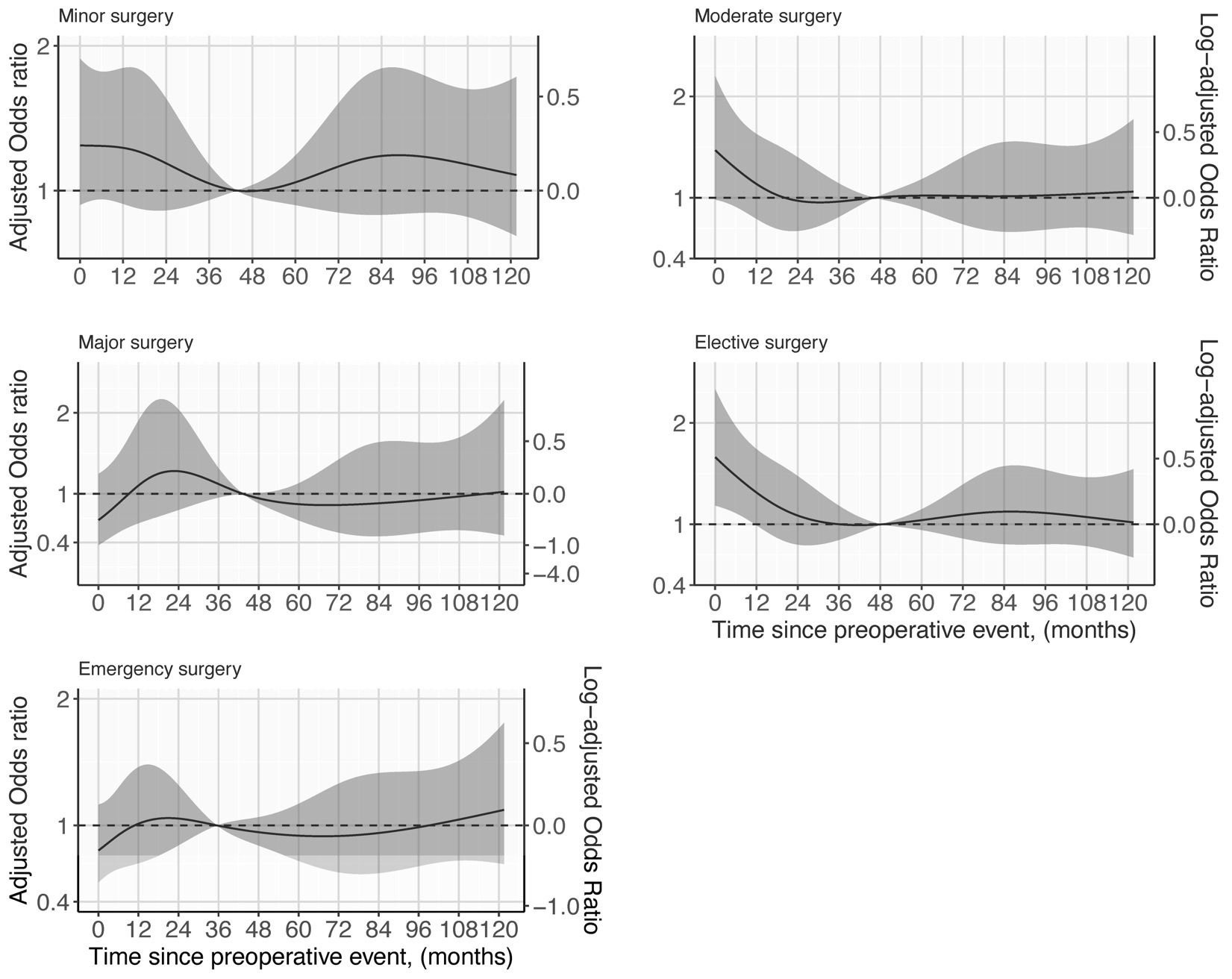


**Figure 50**. Restricted cubic splines logistic regression for 1-year risk of Acute Myocardial infarction after surgery by the time between the most recent cardiovascular event stratified by surgery invasiveness and urgency. The spline was adjusted for age, sex, index of multiple deprivation, hypertension, atrial fibrillation, stable angina, peripheral vascular disease, valvular heart disease, congestive heart failure, respiratory diseases, diabetes mellitus, renal failure, cancer, liver disease, and dementia. The median time between the event and surgery served as the reference.


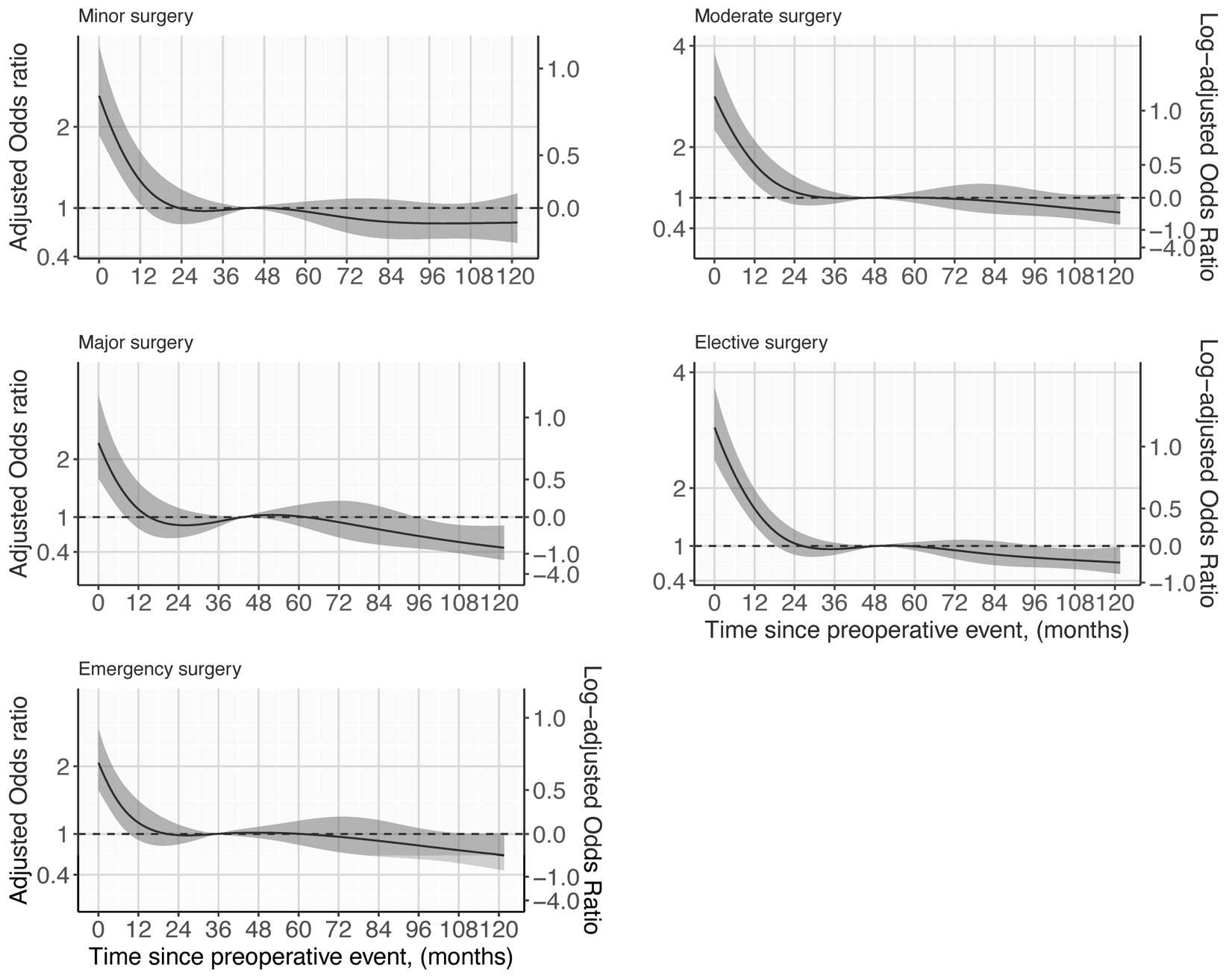


**Figure 51**. Restricted cubic splines logistic regression for 1-year risk of cerebrovascular accident after surgery by the time between the most recent cardiovascular event stratified by surgery invasiveness and urgency. The spline was adjusted for age, sex, index of multiple deprivation, hypertension, atrial fibrillation, stable angina, peripheral vascular disease, valvular heart disease, congestive heart failure, respiratory diseases, diabetes mellitus, renal failure, cancer, liver disease, and dementia. The median time between the event and surgery served as the reference.

##### Myocardial infarction


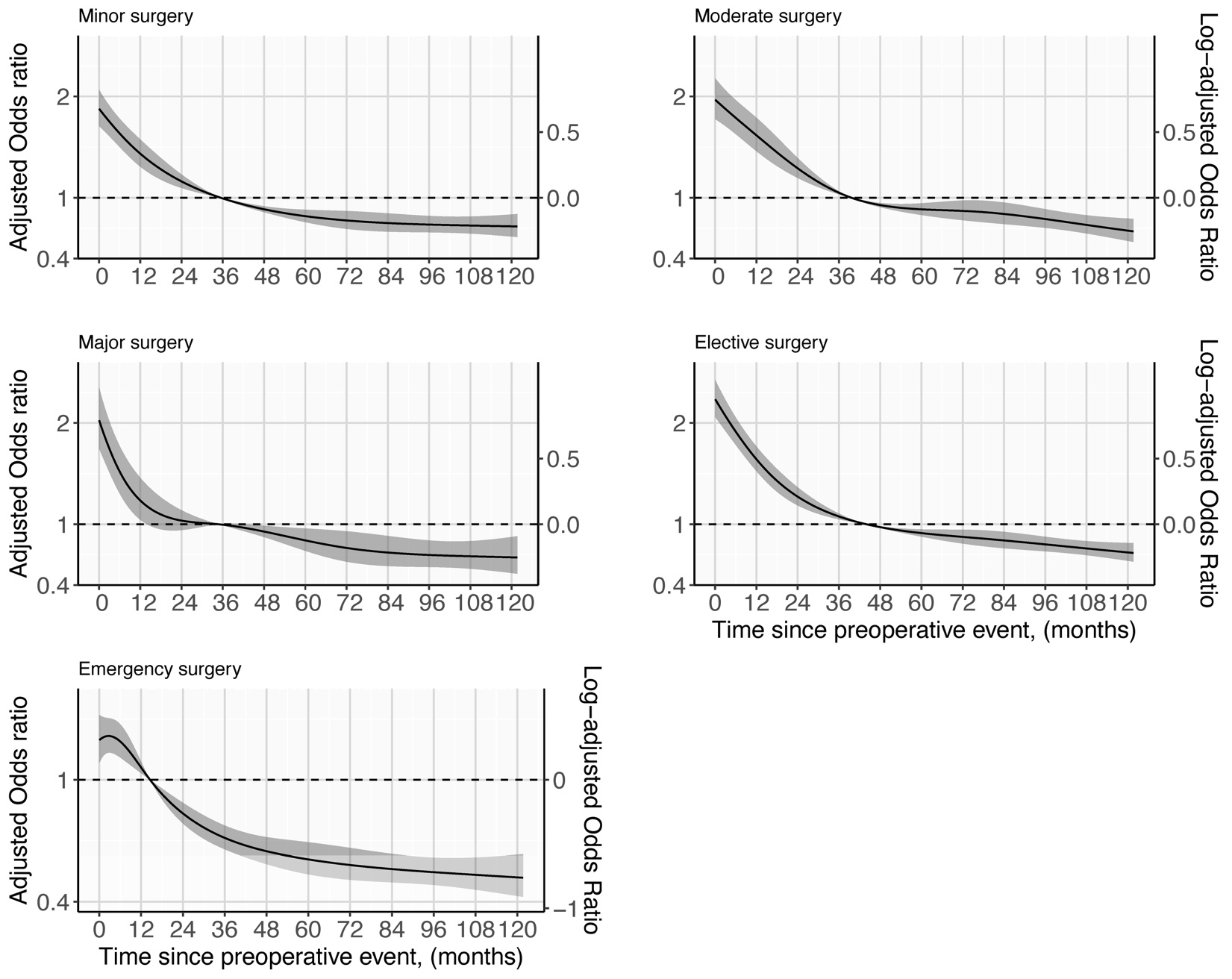


**Figure 52.** Restricted cubic splines logistic regression for 1-year risk of Acute Coronary Syndrome after surgery by the time between the most recent cardiovascular event stratified by surgery invasiveness and urgency. The spline was adjusted for age, sex, index of multiple deprivation, hypertension, atrial fibrillation, stable angina, peripheral vascular disease, valvular heart disease, congestive heart failure, respiratory diseases, diabetes mellitus, renal failure, cancer, liver disease, and dementia. The median time between the event and surgery served as the reference.


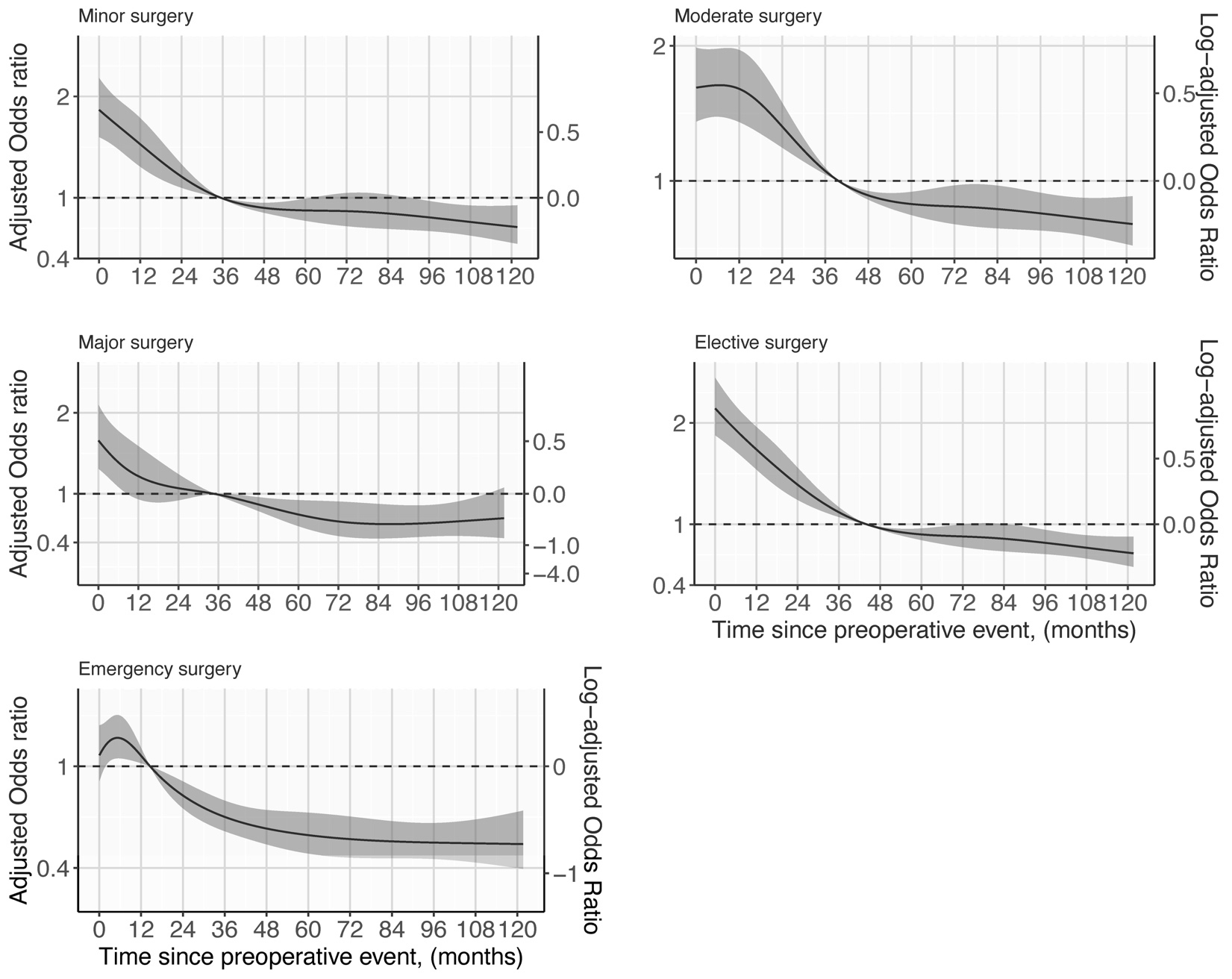


**Figure 53**. Restricted cubic splines logistic regression for 1-year risk of Acute Myocardial infarction after surgery by the time between the most recent cardiovascular event stratified by surgery invasiveness and urgency. The spline was adjusted for age, sex, index of multiple deprivation, hypertension, atrial fibrillation, stable angina, peripheral vascular disease, valvular heart disease, congestive heart failure, respiratory diseases, diabetes mellitus, renal failure, cancer, liver disease, and dementia. The median time between the event and surgery served as the reference.


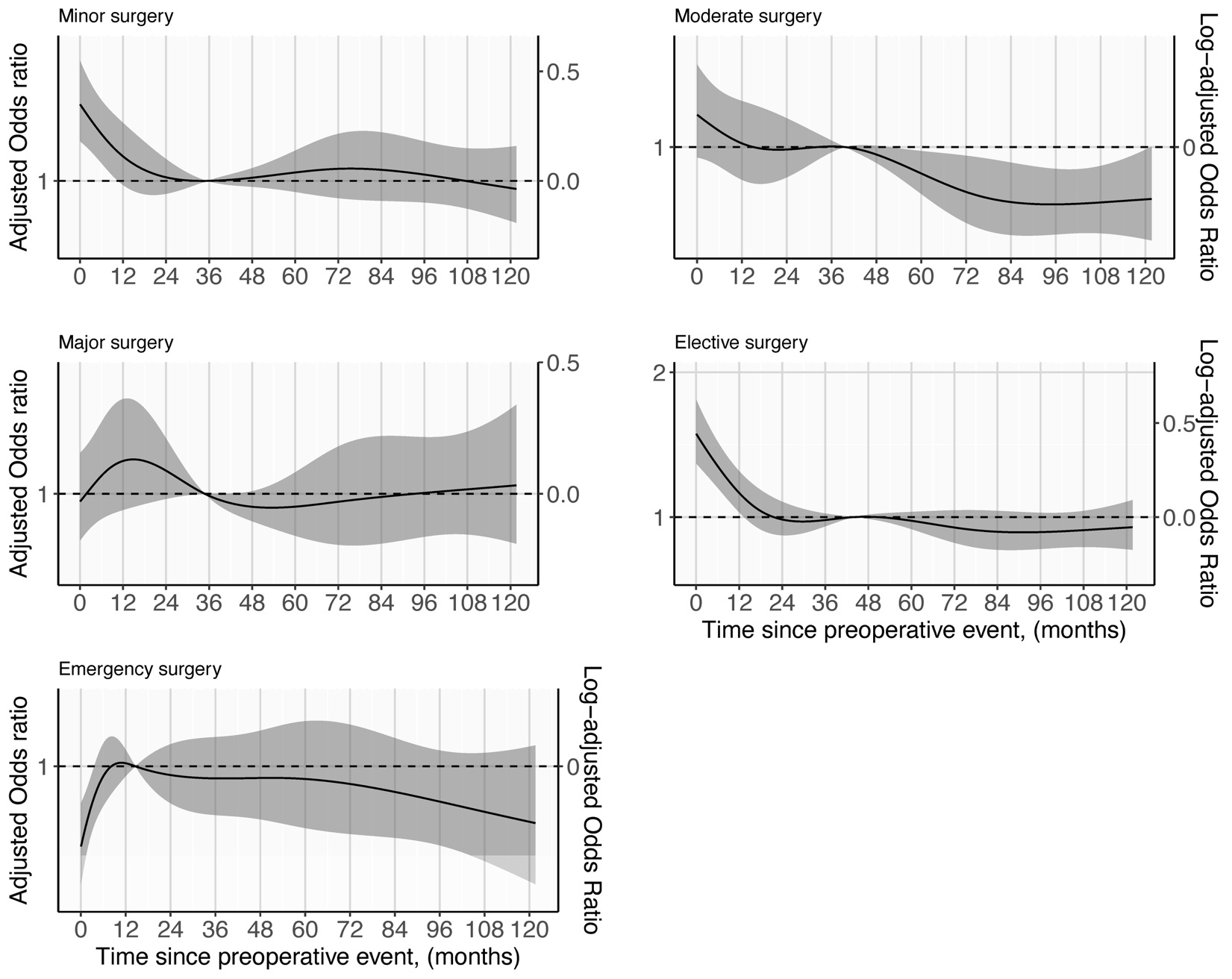


**Figure 54**. Restricted cubic splines logistic regression for 1-year risk of cerebrovascular accident after surgery by the time between the most recent cardiovascular event stratified by surgery invasiveness and urgency. The spline was adjusted for age, sex, index of multiple deprivation, hypertension, atrial fibrillation, stable angina, peripheral vascular disease, valvular heart disease, congestive heart failure, respiratory diseases, diabetes mellitus, renal failure, cancer, liver disease, and dementia. The median time between the event and surgery served as the reference.

##### Transient ischaemic attack


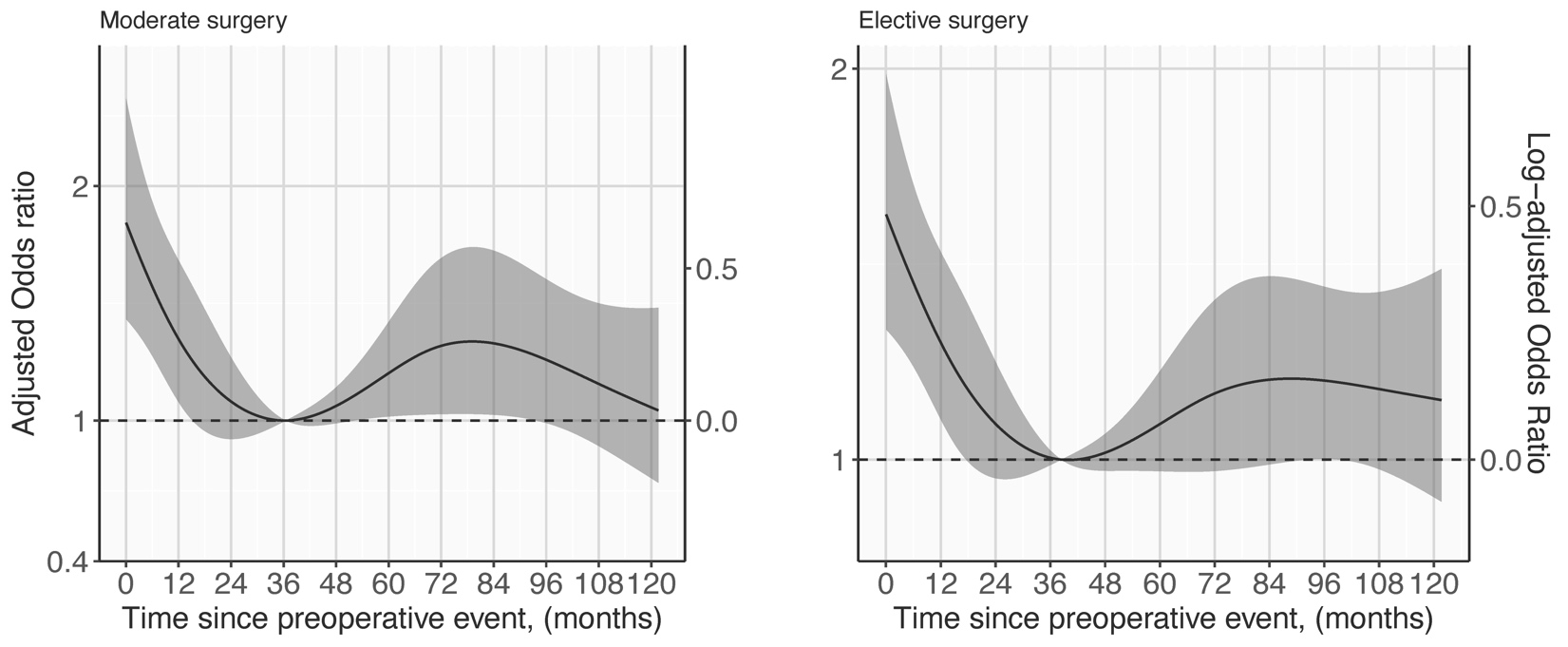


**Figure 55.** Restricted cubic splines logistic regression for 1-year risk of Acute Coronary Syndrome after surgery by the time between the most recent cardiovascular event stratified by surgery invasiveness and urgency. The spline was adjusted for age, sex, index of multiple deprivation, hypertension, atrial fibrillation, stable angina, peripheral vascular disease, valvular heart disease, congestive heart failure, respiratory diseases, diabetes mellitus, renal failure, cancer, liver disease, and dementia. The median time between the event and surgery served as the reference.


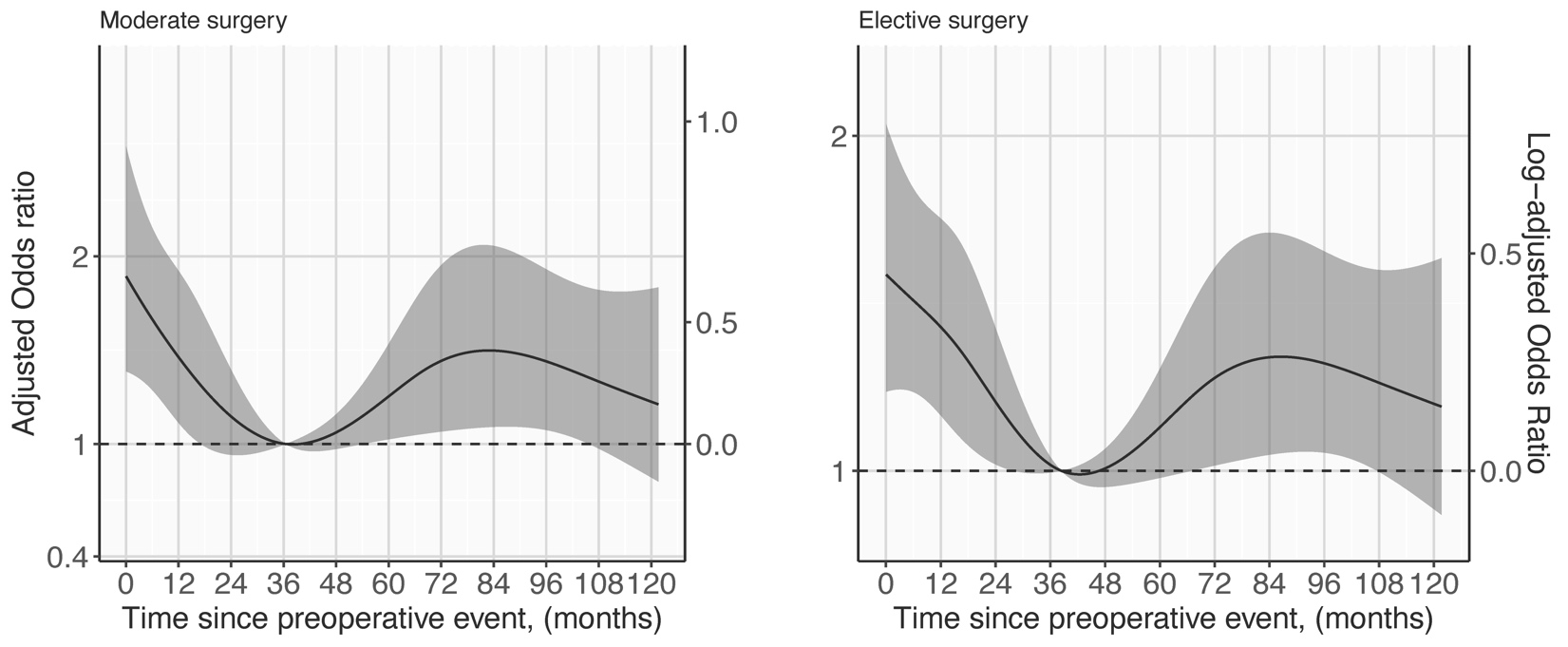


**Figure 56**. Restricted cubic splines logistic regression for 1-year risk of Acute Myocardial infarction after surgery by the time between the most recent cardiovascular event stratified by surgery invasiveness and urgency. The spline was adjusted for age, sex, index of multiple deprivation, hypertension, atrial fibrillation, stable angina, peripheral vascular disease, valvular heart disease, congestive heart failure, respiratory diseases, diabetes mellitus, renal failure, cancer, liver disease, and dementia. The median time between the event and surgery served as the reference.


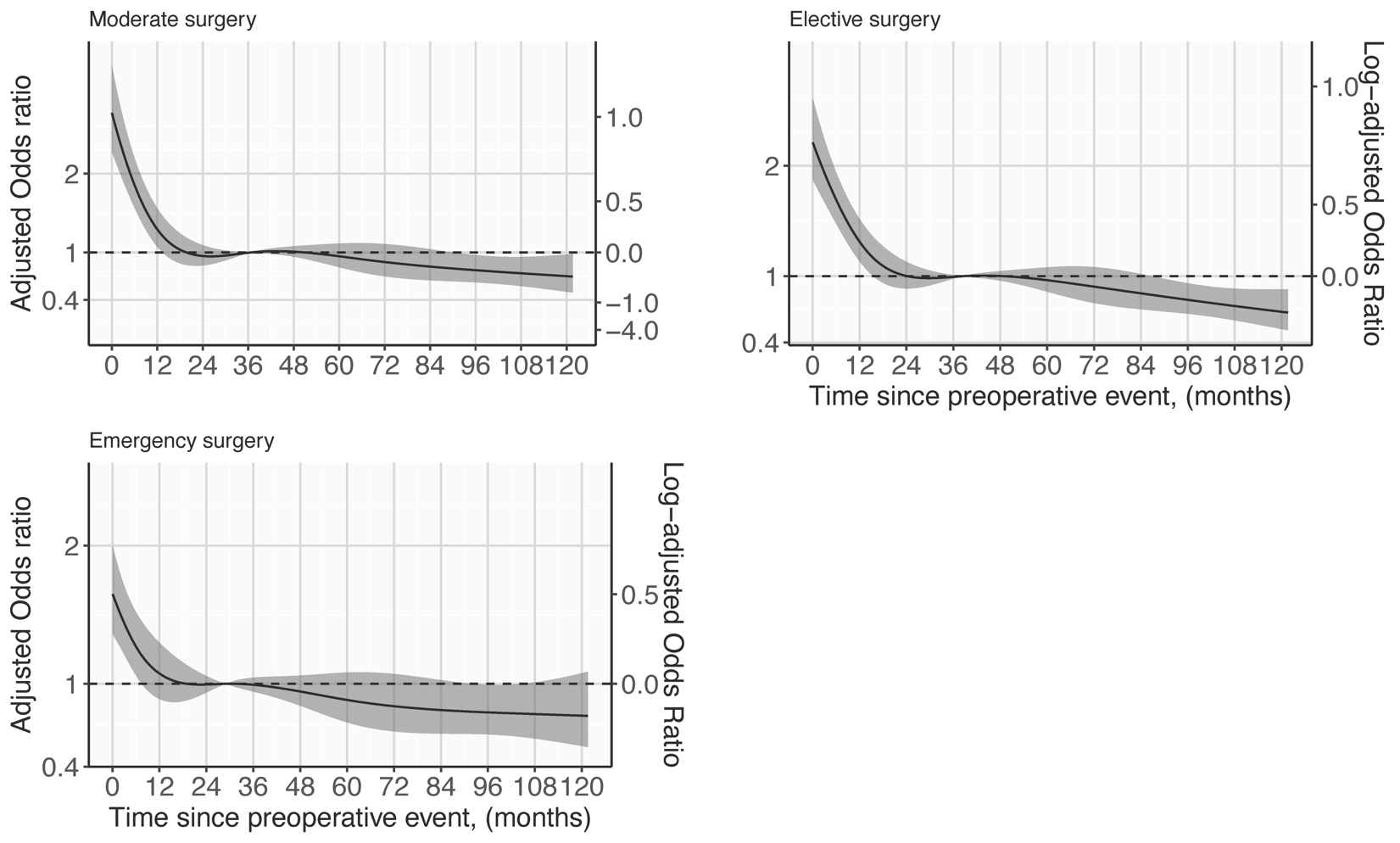


**Figure 57**. Restricted cubic splines logistic regression for 1-year risk of cerebrovascular accident after surgery by the time between the most recent cardiovascular event stratified by surgery invasiveness and urgency. The spline was adjusted for age, sex, index of multiple deprivation, hypertension, atrial fibrillation, stable angina, peripheral vascular disease, valvular heart disease, congestive heart failure, respiratory diseases, diabetes mellitus, renal failure, cancer, liver disease, and dementia. The median time between the event and surgery served as the reference.

##### Unstable angina


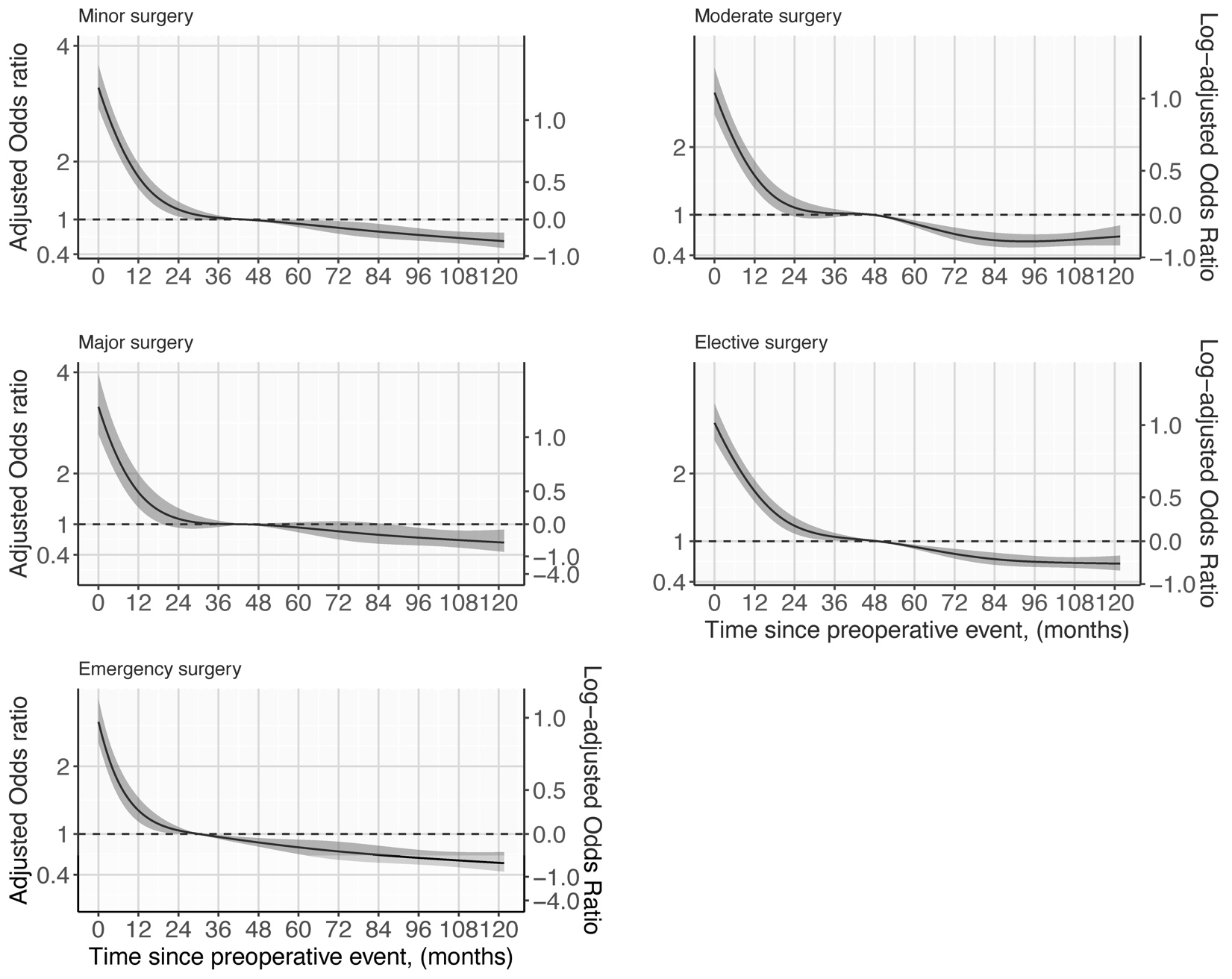


**Figure 58.** Restricted cubic splines logistic regression for 1-year risk of Acute Coronary Syndrome after surgery by the time between the most recent cardiovascular event stratified by surgery invasiveness and urgency. The spline was adjusted for age, sex, index of multiple deprivation, hypertension, atrial fibrillation, stable angina, peripheral vascular disease, valvular heart disease, congestive heart failure, respiratory diseases, diabetes mellitus, renal failure, cancer, liver disease, and dementia. The median time between the event and surgery served as the reference.


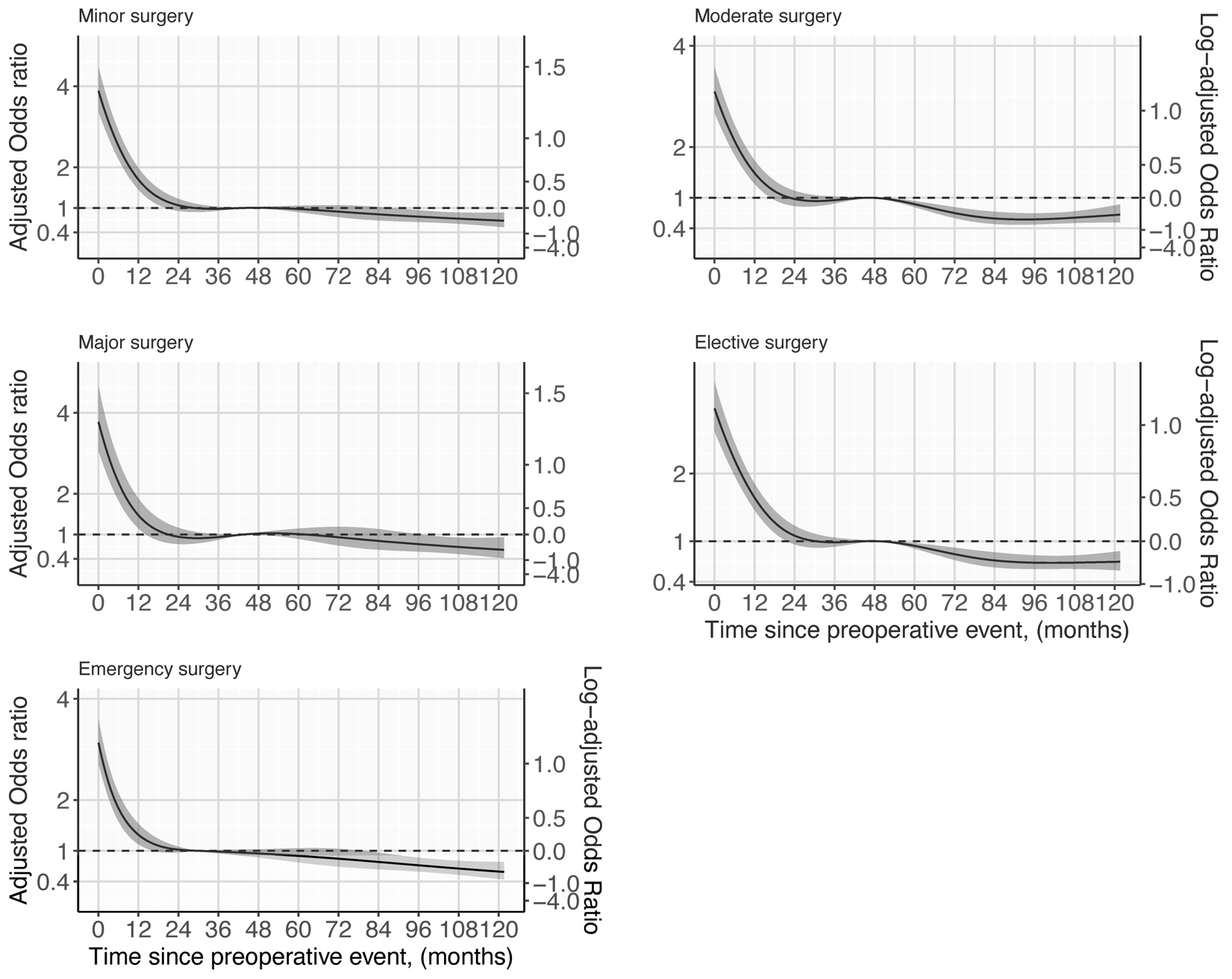


**Figure 59**. Restricted cubic splines logistic regression for 1-year risk of Acute Myocardial infarction after surgery by the time between the most recent cardiovascular event stratified by surgery invasiveness and urgency. The spline was adjusted for age, sex, index of multiple deprivation, hypertension, atrial fibrillation, stable angina, peripheral vascular disease, valvular heart disease, congestive heart failure, respiratory diseases, diabetes mellitus, renal failure, cancer, liver disease, and dementia. The median time between the event and surgery served as the reference.


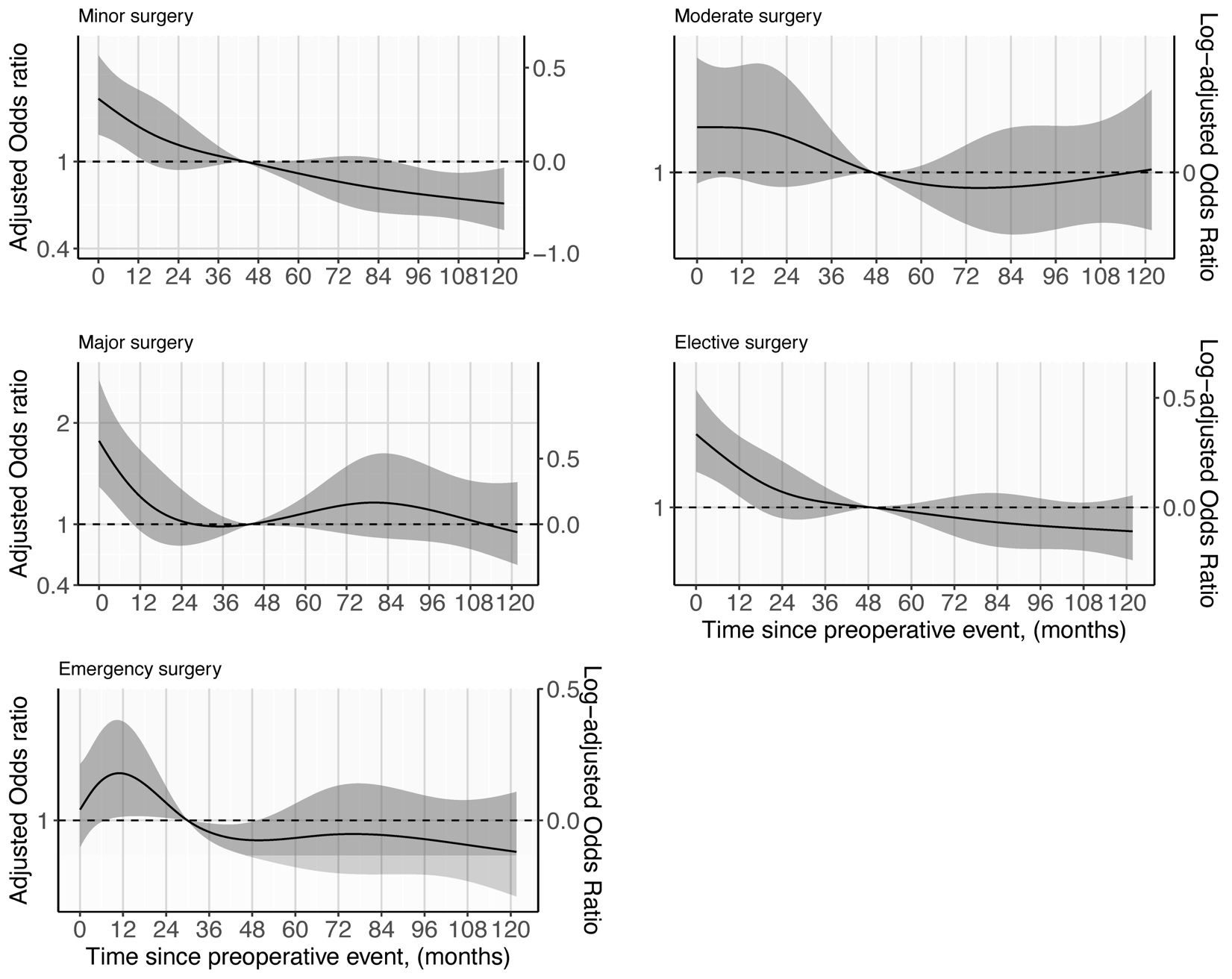


**Figure 60**. Restricted cubic splines logistic regression for 1-year risk of cerebrovascular accident after surgery by the time between the most recent cardiovascular event stratified by surgery invasiveness and urgency. The spline was adjusted for age, sex, index of multiple deprivation, hypertension, atrial fibrillation, stable angina, peripheral vascular disease, valvular heart disease, congestive heart failure, respiratory diseases, diabetes mellitus, renal failure, cancer, liver disease, and dementia. The median time between the event and surgery served as the reference.
